# Supplementary material for: Association of clinical and genetic risk factors with management of dyslipidaemia: analysis of repeated cross-sectional studies in the general population of Lausanne, Switzerland
Source: BMJ Open. 2023 Feb 21;13(2):e065409. doi: 10.1136/bmjopen-2022-065409 (PMC9945309; doi:10.1136/bmjopen-2022-065409)
Supplement: Supplementary data [file bmjopen-2022-065409supp001.pdf]

SUPPLEMENTAL INFORMATION

Supplemental table 1: statins classification regarding generation and potency.

Statin	ATC code	Generation	Potency, no posology	Potency, with posology
lovastatin	C10AA02	First	Low	20 low<br>40 intermediate
pravastatin	C10AA03	First	Low	<40 low<br>40-80 intermediate
fluvastatin	C10AA04	First	Low	10 low<br>40-80 intermediate
simvastatin	C10AA01	Second	Intermediate	20-40 intermediate<br>80 high
pitavastatin	C10AA08	Second	Intermediate	2 intermediate<br>4 high
atorvastatin	C10AA05	Second	High	10-20 intermediate<br>40-80 high
cerivastatin	C10AA06	Third	High	100 intermediate<br>300 high
rosuvastatin	C10AA07	Third	High	5-10 intermediate<br>20 high

Statins were ordered according to their potency. Adapted from [1, 2].

Chekanova V, *et al. BMJ Open* 2023; 13:e065409. doi: 10.1136/bmjopen-2022-065409

Supplemental table 2: LDL-cholesterol thresholds used to define control of dyslipidemia according to the European Society of Cardiology / European Atherosclerosis Society (ESC/EAS) criteria.

**Risk categories according to SCORE**	**Control**
Very high risk	
10–y risk >10%, diabetes or personal history of CVD	<1.8 mmol/L (70 mg/dL)
High risk	
10–y risk 5% – 10%	<2.6 mmol/L (100 mg/dL)
Other categories	
Less than 2 CVD risk factors and 10–y risk <5%	<4.0 mmol/L (155 mg/dL)

ESC/EAS criteria as stated in [3]. Risk categories based on the SCORE equation recalibrated for the Swiss population [4].

Supplemental table 3: LDL-cholesterol thresholds used to define control of dyslipidemia according to the Swiss Group for Lipids and Atherosclerosis.

Risk categories *	Control
High–risk	
10–y risk >20%, diabetes or personal history of CVD	<2.6 mmol/L (100 mg/dL)
Intermediate risk	
At least two CVD risk factors and 10–y risk 10% – 20%	<3.4 mmol/L (130 mg/dL)
Low risk	
At least two CVD risk factors and 10–y risk <10%	<4.2 mmol/L (160 mg/dL)
Very low risk	
Less than 2 CVD risk factors and 10–y risk <10%	<4.2 mmol/L (160 mg/dL)

* Risk calculation as defined in https://www.agla.ch/fr/calculateurs-outils/calculateur-de-risque-du-gsla. According to [5].

Supplemental table 4: list of specific SNPs interacting with the efficacy of statin treatment.

Gene	SNP	Simvastatin	Pravastatin	Atorvastatin	Rosuvastatin	Lovastatin	Fluvastatin
ABCB1	rs1045642	X, ↑		O			
ABCB1	rs1128503	↑					
ABCB1	rs1922242	O MI	O MI low	O MI low			O
ABCB1	rs2032582	↑					
ABCB1	rs3789244	O MI	O MI	O MI			
ABCG2/BCRP	rs2199936				O		
ABCG2/BCRP	rs2231137				X		
ABCG2/BCRP	rs2231142			O	X		X
ABCG8	rs11887534			O			
ACE	rs1799752						↑
APOA1	rs670		O	O			
APOA1	rs5069			O			
APOA5	rs662799	O		O		O	
APOC1	rs4420638	O	O	O	O		
APOE	rs429358	O		↑		↓	
APOE	rs445925			X			
APOE	rs449647			↑			
APOE	rs71352238				O		
APOE	rs7412		↑	↑		↓	
CETP	rs5783961						O
CETP	rs5882	O					
CETP	rs708272	O	O	O			
CLMN	rs8014194	O	O	O			
CYP2C9	rs1057910						X
CYP2D6	rs3892097			X			
CYP3A4	rs2740574			↑			
CYP3A4	rs355599367	X, ↑					
CYP3A4	rs55951658	O					
CYP3A5	rs776746	↓		X, ↓		↓	
CYP7A1	rs3808607			↓			
ESR1	rs2234693			O			
ESR1	rs9340799			-			
HMGCR	rs12916			O			
HMGCR	rs17238540		-				
HMGCR	rs17244841		↓				
HMGCR	rs17671591			X			
HMGCR	rs5908			-			
KIF6	rs20455		O	O			
LDLR	rs5925						↑
LPA	rs10455872			-	O		
LPL	rs328				O		
PCSK9	rs17111584				O		
PON1	rs662		O				
SCAP	rs12487736	↑					
SCCO1B1	rs2306283		↓		X		↑
SLCO1B1	rs11045819						↑
SLCO1B1	rs4149015		X				
SLCO1B1	rs4149056	X, ↓	X, ↓	X, ↓	X		
SOD2	rs4880				↑		
SREBF2	rs4822063			↓			
TLR4	rs4986790		O				

↑, increases effect; ↓, decreases effect; X, safety issues; O, other; MI, myocardial infarction. Data adapted from [6].

Supplemental table 5: data used to compute the genetic risk scores for total, LDL and HDL-cholesterol levels.

Score / Locus	Chr	SNP	Alleles (+/-)	Minor allele	β for weighting
**Total cholesterol**					
ASAP3	1	rs1077514	T/C	C	-0.030
LDLRAP1	1	rs12027135	T/A	A	-1.22
PCSK9	1	rs2479409	G/A	G	1.96
MOSC1	1	rs2807834	G/T	T	-1.38
ANGPTL3	1	rs3850634	T/G	G	-2.60
IRF2BP2	1	rs514230	T/A	A	-1.36
SORT1	1	rs629301	T/G	G	-5.41
EVI5	1	rs7515577	A/C	C	-1.18
INSIG2	2	rs10490626	G/A	A	0.042
UGT1A1	2	rs11563251	T/C	T	0.037
FAM117B	2	rs11694172	G/A	G	0.028
GCKR	2	rs1260326	T/C	T	1.91
APOB	2	rs1367117	A/G	A	4.16
LOC8431	2	rs2030746	T/C	T	0.020
ABCB11	2	rs2287623	G/A	G	0.027
ABCG5/8	2	rs4299376	G/T	G	3.01
RAB3GAP1	2	rs6759321	T/G	T	1.18
PXK	3	rs13315871	G/A	A	-0.036
RAF1	3	rs2290159	G/C	C	-1.42
CMTM6	3	rs7640978	C/T	T	-0.038
LRPAP1	4	rs6831256	G/A	G	-0.022
HMGCR	5	rs12916	C/T	C	2.84
CSNK1G3	5	rs4530754	A/G	G	-0.023
TIMD4	5	rs6882076	C/T	T	-1.98
LPA	6	rs1564348	C/T	C	2.18
HFE	6	rs1800562	G/A	A	-2.16
KCNK17	6	rs2758886	A/G	A	0.023
MYLIP	6	rs3757354	C/T	T	-1.46
HBS1L	6	rs9376090	C/T	T	-0.025
FRK	6	rs9488822	A/T	T	-1.18
GPR146	7	rs1997243	G/A	G	0.033
DNAH11	7	rs2285942	T/C	T	1.70
SOX17	8	rs10102164	A/G	A	0.030
CYP741	8	rs1030431	A/G	A	1.26
PLEC1	8	rs11136341	G/A	G	1.34
NAT2	8	rs1961456	G/A	G	1.07
PPP1R3B	8	rs2126259	C/T	T	-3.14
TRPS1	8	rs2737229	A/C	C	-1.11
TRIB1	8	rs2954022	C/A	A	-2.30
ABCA1	9	rs1883025	C/T	T	-2.24
VLDLR	9	rs3780181	A/G	G	-0.044
TTC39B	9	rs581080	C/G	G	-1.57
ABO	9	rs651007	T/C	T	2.30
VIM-CUBN	10	rs10904908	G/A	G	0.025
GPAM	10	rs2255141	A/G	A	1.14
MARCH8-ALOX5	10	rs970548	C/A	C	-0.026

Chekanova V, *et al. BMJ Open* 2023; 13:e065409. doi: 10.1136/bmjopen-2022-065409

SPTY2D1	11	rs10832963	G/T	T	-1.06
ST3GAL4	11	rs11220463	T/A	T	2.01
PHLDB1	11	rs11603023	T/C	T	0.022
FADS1-2-3	11	rs174550	T/C	C	-1.78
UBASH3B	11	rs7941030	C/T	C	0.97
APOA1	11	rs964184	G/C	G	4.68
PHC1-A2ML1	12	rs4883201	A/G	G	-0.035
LIPC	15	rs1532085	A/G	A	1.54
HPR	16	rs2000999	A/G	A	2.34
CETP	16	rs3764261	A/C	A	1.67
DLG4	17	rs314253	T/C	C	-0.023
OSBPL7	17	rs7206971	A/G	A	1.01
LIPG	18	rs7239867	G/A	A	-1.94
CILP2	19	rs10401969	T/C	C	-4.74
FLJ36070	19	rs492602	G/A	G	1.27
LDLR	19	rs6511720	G/T	T	-7.09
HNF4A	20	rs1800961	C/T	T	-4.73
ERGIC3	20	rs2277862	C/T	T	-1.19
MAFB	20	rs2902940	A/G	G	-1.38
TOP1	20	rs4297946	C/G	C	1.52
TOM1	22	rs138777	A/G	A	0.021
PPARA	22	rs4253772	T/C	T	0.032
**LDL cholesterol**					
LDLRAP1	1	rs12027135	T/A	A	-1.10
PIGV-NR0B2	1	rs12748152	T/C	T	0.05
PCSK9	1	rs2479409	G/A	G	2.01
ANXA9-CERS2	1	rs267733	A/G	G	-0.03
MOSC1	1	rs2807834	G/T	T	-1.09
ANGPTL3	1	rs3850634	T/G	G	-1.59
IRF2BP2	1	rs514230	T/A	A	-1.13
SORT1	1	rs629301	T/G	G	-5.65
INSIG2	2	rs10490626	G/A	A	-0.05
UGT1A1	2	rs11563251	T/C	T	0.03
FN1	2	rs1250229	C/T	T	-0.02
APOB	2	rs1367117	A/G	A	4.05
LOC84931	2	rs2030746	T/C	T	0.02
EHBP1	2	rs2710642	A/G	G	-0.02
ABCG5/8	2	rs4299376	G/T	G	2.75
ACAD11	3	rs17404153	G/T	T	-0.03
CMTM6	3	rs7640978	C/T	T	-0.04
LRPAP1	4	rs6831256	G/A	G	0.02
HMGCR	5	rs12916	C/T	C	2.45
CSNK1G3	5	rs4530754	A/G	G	-0.03
TIMD4	5	rs6882076	C/T	T	-1.67
FRK	6	rs11153594	C/T	T	-0.89
LPA	6	rs1564348	C/T	C	1.95
HFE	6	rs1800562	G/A	A	-2.22
MYLIP	6	rs3757354	C/T	T	-1.43
DNAH11	7	rs12670798	C/T	C	1.26
NPC1L1	7	rs217386	G/A	A	-1.17
SOX17	8	rs10102164	A/G	A	0.03
CYP741	8	rs1030431	A/G	A	0.95

PLEC1	8	rs11136341	G/A	G	1.40
PPP1R3B	8	rs2126259	C/T	T	-3.14
TRIB1	8	rs2954022	C/A	A	-1.84
VLDLR	9	rs3780181	A/G	G	-0.04
ABO	9	rs649129	T/C	T	2.05
GPAM	10	rs1129555	A/G	A	1.08
ST3GAL4	11	rs11220462	A/G	A	1.95
FADS1-2-3	11	rs174583	C/T	T	-1.71
APOA1	11	rs964184	G/C	G	2.85
BRCA2	13	rs4942486	T/C	T	0.02
HPR	16	rs2000999	A/G	A	2.00
CETP	16	rs247616	C/T	T	-1.45
DLG4	17	rs314253	T/C	C	-0.02
OSBPL7	17	rs7206971	A/G	A	1.01
CILP2	19	rs10401969	T/C	C	-3.11
SNX5	20	rs2328223	C/A	C	0.03
MAFB	20	rs2902941	A/G	G	-0.98
SPTLC3	20	rs364585	G/A	A	-0.03
TOP1	20	rs909802	T/C	T	1.41
PPARA	22	rs4253772	T/C	T	0.03
MTMR3	22	rs5763662	T/C	T	0.08
**HDL cholesterol**					
HDGF-PMVK	1	rs12145743	G/T	G	0.200
PIGV-NR0B2	1	rs12748152	C/T	T	-0.051
ZNF648	1	rs1689800	G/A	G	-0.47
ANGPTL1	1	rs4650994	G/A	G	0.021
PABPC4	1	rs4660293	G/A	G	-0.48
GALNT2	1	rs4846914	G/A	G	-0.61
APOB	2	rs1042034	T/C	C	0.90
COBLL1	2	rs12328675	T/C	C	0.68
IRS1	2	rs1515100	A/C	C	0.46
STAB1	3	rs13326165	A/G	A	0.029
ACAD11	3	rs17404153	G/T	T	0.028
RBM5	3	rs2013208	T/C	T	0.025
ATG7	3	rs2606736	C/T	C	0.025
GSK3B	3	rs6805251	T/C	T	0.020
C4orf52	4	rs10019888	A/G	G	-0.027
SLC39A8	4	rs13107325	T/C	T	-0.84
ADH5	4	rs2602836	A/G	A	0.019
FAM13A	4	rs3822072	G/A	A	-0.025
ARL15	5	rs6450176	A/G	A	-0.49
RSPO3	6	rs1936800	C/T	C	0.020
C6orf106	6	rs2814944	A/G	A	-0.49
CITED2	6	rs605066	C/T	C	-0.39
VEGFA	6	rs998584	C/A	A	-0.026
MLXIPL	7	rs17145738	C/T	T	0.57
TMEM176A	7	rs17173637	T/C	C	-0.036
SNX13	7	rs4142995	G/T	T	-0.026
KLF14	7	rs4731702	C/T	T	0.59
IKZF1	7	rs4917014	G/T	G	0.022
DAGLB	7	rs702485	G/A	G	0.024
TRIB1	8	rs10808546	C/T	T	0.61

LPL	8	rs12678919	A/G	G	2.25
TRPS1	8	rs2293889	T/G	T	-0.44
PPP1R3B	8	rs9987289	A/G	A	-1.21
ABCA1	9	rs1883025	T/C	T	-0.94
TTC39B	9	rs643531	C/A	C	-0.72
MARCH8-ALOX5	10	rs970548	C/A	C	0.026
OR4C46	11	rs11246602	C/T	C	0.034
KAT5	11	rs12801636	A/G	A	0.024
FADS1-2-3	11	rs174601	T/C	T	-0.73
AMPD3	11	rs2923084	G/A	G	-0.41
LRP4	11	rs3136441	T/C	C	0.78
MOGAT2-DGAT2	11	rs499974	C/A	A	-0.026
UBASH3B	11	rs7115089	C/G	G	0.31
APOA1	11	rs964184	G/C	G	-1.50
LRP1	12	rs3741414	C/T	T	0.46
SBNO1	12	rs4759375	C/T	T	0.86
ZNF664	12	rs4765127	G/T	T	0.44
PDE3A	12	rs7134375	C/A	A	0.40
MVK	12	rs7134594	C/T	C	-0.44
SCARB1	12	rs838880	T/C	C	0.61
LIPC	15	rs1532085	G/A	A	1.45
LACTB	15	rs2652834	A/G	A	-0.39
FTO	16	rs1121980	G/A	A	-0.020
LCAT	16	rs16942887	G/A	A	1.27
CMIP	16	rs2925979	T/C	T	-0.45
CETP	16	rs3764261	C/A	A	3.39
PGS1	17	rs4082919	G/T	G	-0.40
ABCA8	17	rs4148008	G/C	G	-0.42
STARD3	17	rs881844	C/G	C	-0.51
MC4R	18	rs12967135	A/G	A	-0.42
LIPG	18	rs7241918	G/T	G	-1.31
HAS1	19	rs17695224	G/A	A	-0.029
LILRA3	19	rs386000	G/C	C	0.83
ANGPTL4	19	rs7255436	C/A	C	-0.45
PEPD	19	rs731839	A/G	G	-0.022
LOC55908	19	rs737337	C/T	C	-0.64
HNF4A	20	rs1800961	T/C	T	-1.88
PLTP	20	rs6065906	C/T	C	-0.93
UBE2L3	22	rs181362	T/C	T	-0.46

Chr, chromosome; SNP, single nucleotide polymorphism. Adapted from [7].

Supplemental table 6: bivariate comparisons of socio-economic and clinical characteristics between retained and excluded participants. Data from the baseline (2003-2006), first (2009-2012) and second (2014-2017) follow-ups of the CoLaus|PsyCoLaus study, Lausanne, Switzerland.

	Baseline			First follow-up			Second follow-up		
	**Included**	**Excluded**	**p-value**	**Included**	**Excluded**	**p-value**	**Included**	**Excluded**	**p-value**
N	**617**	**92**		**844**	**212**		**798**	**353**	
Age (years)	61.6 ± 8.5	58.1 ± 9.6	<0.001	64.5 ± 8.8	61.7 ± 10.4	<0.001	68.1 ± 9.2	67.5 ± 10.3	0.309
Women (%)	263 (42.6)	28 (30.4)	0.027	409 (48.5)	90 (42.5)	0.117	401 (50.3)	166 (47.0)	0.313
Swiss national (%)	430 (69.7)	59 (64.1)	0.282	600 (71.1)	116 (54.7)	<0.001	563 (70.6)	191 (54.1)	<0.001
Education (%)			0.301			0.027			0.714
High	66 (10.7)	5 (5.5)		123 (14.6)	47 (22.2)		120 (15.0)	54 (15.3)	
Middle	132 (21.4)	20 (22.0)		185 (21.9)	43 (20.3)		180 (22.6)	72 (20.4)	
Low	419 (67.9)	66 (72.5)		536 (63.5)	122 (57.6)		498 (62.4)	227 (64.3)	
Married/couple (%)	428 (69.4)	65 (71.4)	0.690	505 (59.8)	121 (57.1)	0.465	448 (56.1)	166 (53.9)	0.501
BMI (kg/m^2)	28.0 ± 4.5	28.1 ± 4.8	0.792	27.6 ± 4.8	28.0 ± 5.0	0.398			
BMI categories (%)			0.979			0.986			0.197
Normal	155 (25.1)	24 (26.1)		243 (28.8)	53 (28.2)		254 (31.8)	59 (26.6)	
Overweight	277 (44.9)	41 (44.6)		378 (44.8)	85 (45.2)		327 (41.0)	91 (41.0)	
Obese	185 (30.0)	27 (29.4)		223 (26.4)	50 (26.6)		217 (27.2)	72 (32.4)	
Smoking (%)			0.064			0.099			0.067
Never	210 (34.0)	39 (42.9)		295 (35.0)	73 (34.8)		290 (36.3)	103 (38.7)	
Former	255 (41.3)	26 (28.6)		385 (45.6)	83 (39.5)		365 (45.7)	102 (38.4)	
Current	152 (24.6)	26 (28.6)		164 (19.4)	54 (25.7)		143 (17.9)	61 (22.9)	
Alcohol drinker (%)	456 (73.9)	65 (70.7)	0.510	632 (74.9)	147 (69.3)	0.101	525 (72.3)	149 (62.3)	0.004
Treatment for									
Hypertension	303 (49.1)	49 (53.3)	0.457	436 (51.7)	101 (47.6)	0.296	425 (53.3)	190 (53.8)	0.859
Diabetes	95 (15.4)	24 (26.1)	0.010	148 (17.5)	40 (18.9)	0.650	157 (19.7)	69 (19.6)	0.960
Parental history (%)	164 (26.6)	24 (26.1)	0.920	218 (25.8)	52 (24.5)	0.698	211 (26.4)	64 (18.1)	0.002
CVD risk (%)			0.551			0.717			<0.001

Low	267 (43.3)	36 (39.1)	367 (43.5)	89 (42.0)	356 (44.6)	103 (29.2)
Intermediate	182 (29.5)	26 (28.3)	203 (24.1)	48 (22.6)	165 (20.7)	121 (34.3)
High	168 (27.2)	30 (32.6)	274 (32.5)	75 (35.4)	277 (34.7)	129 (36.5)

BMI, body mass index; CVD, cardio vascular disease. Results are expressed as number of participants (column %) for categorical variables and as average ± standard deviation for continuous variables. Between-groups comparisons performed using chi-square for categorical variables and student's t-test for continuous variables.

Supplemental table 7: bivariate comparison of the statin generation and potency among participants treated for dyslipidemia, according to controlled and uncontrolled status as per European Society of Cardiology / European Atherosclerosis Society (ESC/EAS) criteria. Data from the first (2009-2012) and second (2014-2017) follow-ups of the CoLaus|PsyCoLaus study, Lausanne, Switzerland. Analysis restricted to participants taking statins.

	**First follow-up**			**Second follow-up**		
	**Uncontrolled**	**Controlled**	**p-value**	**Uncontrolled**	**Controlled**	**p-value**
N	**373**	**288**		**428**	**370**	
Generation			0.004			0.212
First	99 (26.5)	50 (17.4)		54 (16.5)	32 (12.2)	
Second	249 (66.8)	204 (70.8)		233 (71.0)	188 (71.8)	
Third	25 (6.7)	34 (11.8)		41 (12.5)	42 (16.0)	
Potency §			0.018			0.018
Low	99 (26.5)	50 (17.4)		54 (16.5)	32 (12.2)	
Intermediate	130 (34.9)	109 (37.9)		107 (32.6)	66 (25.2)	
High	144 (38.6)	129 (44.8)		167 (50.9)	164 (62.6)	
N	**348**	**274**		**328**	**262**	
Potency †			0.911			0.067
Low	166 (47.7)	126 (46.0)		117 (40.6)	72 (31.2)	
Intermediate	153 (44.0)	124 (45.3)		125 (43.4)	111 (48.0)	
High	29 (8.3)	24 (8.7)		46 (16.0)	48 (20.8)	

§, not considering posology; †, considering posology. Results are expressed as number of participants (column %). Between-groups comparisons performed using chi-square.

Chekanova V, *et al. BMJ Open* 2023; 13:e065409. doi: 10.1136/bmjopen-2022-065409

Supplemental table 8: multivariable analysis of the factors associated with dyslipidemia control as per European Society of Cardiology / European Atherosclerosis Society criteria. Data from the first (2009-2012) and second (2014-2017) follow-ups of the CoLaus|PsyCoLaus study, Lausanne, Switzerland. Analysis taking into account statin potency, but not considering posology.

	**First follow-up**		**Second follow-up**	
	**OR (95% CI)**	**p-value**	**OR (95% CI)**	**p-value**
Age (per 10 years increase)	0.31 (0.23 - 0.41)	<0.001	0.36 (0.27 - 0.47)	<0.001
Man vs. woman	0.59 (0.38 - 0.90)	0.014	1.36 (0.88 - 2.11)	0.171
Swiss vs. Non-Swiss	1.16 (0.76 - 1.78)	0.495	1.17 (0.75 - 1.81)	0.487
Education				
High	1 (ref.)		1 (ref.)	
Middle	0.76 (0.39 - 1.48)	0.415	1.04 (0.52 - 2.08)	0.908
Low	0.87 (0.48 - 1.57)	0.649	1.02 (0.56 - 1.87)	0.937
p-value for trend	0.649		0.937	
Married vs. not married	1.23 (0.83 - 1.84)	0.299	1.20 (0.80 - 1.79)	0.386
Body mass index categories				
Normal	1 (ref.)		1 (ref.)	
Overweight	0.86 (0.53 - 1.38)	0.523	0.57 (0.35 - 0.93)	0.025
Obese	0.93 (0.54 - 1.61)	0.807	0.53 (0.30 - 0.94)	0.031
p-value for trend	0.807		0.031	
Smoking categories				
Never	1 (ref.)		1 (ref.)	
Former	1.13 (0.72 - 1.77)	0.590	1.19 (0.77 - 1.85)	0.428
Current	0.86 (0.48 - 1.55)	0.614	0.90 (0.49 - 1.65)	0.729
p-value for trend	0.614		0.729	
Alcohol drinker (yes vs. no)	0.81 (0.51 - 1.27)	0.360	0.68 (0.43 - 1.08)	0.101
AntiHTA ttt (yes vs. no)	0.95 (0.62 - 1.47)	0.821	0.80 (0.51 - 1.25)	0.327
Parental history (yes vs. no)	1.25 (0.79 - 1.99)	0.338	0.75 (0.47 - 1.21)	0.240
CVD risk				
Other	1 (ref.)		1 (ref.)	
High	0.63 (0.39 - 1.02)	0.060	1.37 (0.78 - 2.40)	0.275
Very high	0.08 (0.05 - 0.14)	<0.001	0.35 (0.21 - 0.58)	<0.001
p-value for trend	<0.001		<0.001	
LDL genetic risk score quartiles				
First	1 (ref.)		1 (ref.)	
Second	0.89 (0.52 - 1.53)	0.680	1.63 (0.92 - 2.86)	0.092
Third	0.92 (0.54 - 1.57)	0.757	1.76 (1.02 - 3.03)	0.041
Fourth	1.11 (0.65 - 1.91)	0.702	1.58 (0.90 - 2.77)	0.112
p-value for trend	0.694		0.109	
Number of drugs (per 1 unit)	1.14 (1.05 - 1.25)	0.002	1.06 (0.99 - 1.15)	0.099
Statin potency, no posology				
Low	1 (ref.)		1 (ref.)	
Intermediate	1.98 (1.18 - 3.34)	0.010	1.50 (0.80 - 2.82)	0.209

Chekanova V, *et al. BMJ Open* 2023; 13:e065409. doi: 10.1136/bmjopen-2022-065409

High	2.08 (1.25 - 3.46)	0.005	2.25 (1.26 - 4.01)	0.006
p-value for trend	0.005		0.006	
Fibrates	NC		2.16 (0.16 - 29.1)	0.560
Other hypolipidemic drugs	0.95 (0.41 - 2.22)	0.905	1.07 (0.48 - 2.37)	0.867

OR, odds ratio; CI, confidence interval; antiHTA ttt, antihypertensive drug treatment; NC, not computable. Results are expressed as odds ratio and (95% confidence interval). Statistical analysis using logistic regression.

Chekanova V, *et al. BMJ Open* 2023; 13:e065409. doi: 10.1136/bmjopen-2022-065409

Supplemental table 9: multivariable analysis of the factors associated with dyslipidemia control as per European Society of Cardiology / European Atherosclerosis Society criteria. Data from the first (2009-2012) and second (2014-2017) follow-ups of the CoLaus|PsyCoLaus study, Lausanne, Switzerland. Analysis taking into account statin potency and posology.

	**First follow-up**		**Second follow-up**	
	**OR (95% CI)**	**p-value**	**OR (95% CI)**	**p-value**
Age (per 10 years increase)	0.31 (0.24 - 0.42)	<0.001	0.37 (0.27 - 0.50)	<0.001
Man vs. woman	0.56 (0.36 - 0.86)	0.008	1.45 (0.91 - 2.31)	0.120
Swiss vs. Non-Swiss	1.14 (0.74 - 1.78)	0.548	1.11 (0.69 - 1.76)	0.672
Education				
High	1 (ref.)		1 (ref.)	
Middle	0.74 (0.37 - 1.48)	0.396	0.95 (0.46 - 1.95)	0.882
Low	0.86 (0.47 - 1.56)	0.619	0.94 (0.50 - 1.76)	0.835
p-value for trend	0.619		0.835	
Married vs. not married	1.25 (0.83 - 1.88)	0.29	1.17 (0.76 - 1.80)	0.481
Body mass index categories				
Normal	1 (ref.)		1 (ref.)	
Overweight	0.87 (0.54 - 1.42)	0.586	0.62 (0.37 - 1.05)	0.073
Obese	0.89 (0.50 - 1.56)	0.677	0.56 (0.31 - 1.02)	0.058
p-value for trend	0.677		0.058	
Smoking categories				
Never	1 (ref.)		1 (ref.)	
Former	1.12 (0.71 - 1.77)	0.629	1.12 (0.7 - 1.78)	0.636
Current	0.91 (0.50 - 1.65)	0.747	0.82 (0.42 - 1.58)	0.545
p-value for trend	0.747		0.545	
Alcohol drinker (yes vs. no)	0.73 (0.45 - 1.16)	0.183	0.58 (0.35 - 0.95)	0.031
AntiHTA ttt (yes vs. no)	0.99 (0.64 - 1.55)	0.977	0.89 (0.55 - 1.42)	0.618
Parental history (yes vs. no)	1.24 (0.77 - 1.98)	0.382	0.85 (0.51 - 1.41)	0.529
CVD risk				
Other	1 (ref.)		1 (ref.)	
High	0.64 (0.39 - 1.04)	0.074	1.29 (0.71 - 2.33)	0.400
Very high	0.08 (0.05 - 0.15)	<0.001	0.35 (0.20 - 0.61)	<0.001
p-value for trend	<0.001		<0.001	
LDL genetic risk score quartiles				
First	1 (ref.)		1 (ref.)	
Second	1.01 (0.58 - 1.75)	0.979	1.63 (0.89 - 2.96)	0.111
Third	1.15 (0.66 - 1.99)	0.623	1.89 (1.06 - 3.36)	0.031
Fourth	1.24 (0.71 - 2.16)	0.452	1.68 (0.93 - 3.02)	0.085
p-value for trend	0.392		0.073	
Number of drugs (per 1 unit)	1.13 (1.03 - 1.23)	0.008	1.04 (0.96 - 1.13)	0.366
Statin potency, with posology				
Low	1 (ref.)		1 (ref.)	
Intermediate	1.10 (0.73 - 1.65)	0.656	1.37 (0.86 - 2.17)	0.183

High	1.35 (0.63 - 2.88)	0.437	1.69 (0.89 - 3.21)	0.110
p-value for trend	0.437		0.110	
Fibrates	NC		2.20 (0.16 - 31.2)	0.559
Other hypolipidemic drugs	0.91 (0.38 - 2.21)	0.839	1.06 (0.45 - 2.47)	0.898

OR, odds ratio; CI, confidence interval; antiHTA ttt, antihypertensive drug treatment; NC, not computable. Results are expressed as odds ratio and (95% confidence interval). Statistical analysis using logistic regression.

Supplemental table 10: bivariate comparisons of socio-economic and clinical characteristics among participants treated for dyslipidemia, according to controlled and uncontrolled status as per the Swiss Group for Lipids and Atherosclerosis criteria. Data from the baseline (2003-2006), first (2009-2012) and second (2014-2017) follow-ups of the CoLaus|PsyCoLaus study, Lausanne, Switzerland.

	**Baseline**			**First follow-up**			**Second follow-up**		
	**Uncontrolled**	**Controlled**	**p-value**	**Uncontrolled**	**Controlled**	**p-value**	**Uncontrolled**	**Controlled**	**p-value**
N	**184**	**433**		**266**	**578**		**139**	**659**	
Age (years)	61.5 ± 8.5	61.7 ± 8.5	0.728	64.5 ± 8.6	64.5 ± 8.9	0.947	67.1 ± 9.0	68.4 ± 9.3	0.139
Women (%)	81 (44.0)	182 (42.0)	0.648	119 (44.7)	290 (50.2)	0.142	72 (51.8)	329 (49.9)	0.688
Swiss national (%)	128 (70.0)	302 (69.6)	0.929	183 (69.1)	417 (72.0)	0.378	105 (74.5)	458 (69.7)	0.261
Education (%)			0.474			0.707			0.354
High	47 (10.9)	66 (10.7)		16 (6.0)	38 (6.6)		10 (7.2)	50 (7.6)	
Middle	98 (22.6)	132 (21.4)		39 (14.7)	89 (15.4)		17 (12.2)	53 (8.0)	
Low	288 (66.5)	419 (67.9)		50 (18.8)	125 (21.6)		23 (16.6)	95 (14.4)	
Married/couple (%)	127 (69.0)	301 (69.5)	0.903	353 (61.1)	505 (59.8)	0.279	74 (53.2)	374 (56.8)	0.448
BMI (kg/m^2)	28.4 ± 4.4	27.8 ± 4.5	0.150	28.2 ± 4.9	27.4 ± 4.7	0.038	28.6 ± 4.8	27.4 ± 4.9	0.008
BMI categories (%)			0.354			0.125			0.033
Normal	40 (21.7)	115 (26.6)		69 (25.9)	174 (30.1)		33 (23.7)	221 (33.5)	
Overweight	83 (45.1)	194 (44.8)		115 (43.2)	263 (45.5)		58 (41.7)	269 (40.8)	
Obese	61 (33.2)	124 (28.6)		82 (30.8)	141 (24.4)		48 (34.5)	169 (25.6)	
Smoking (%)			0.285			0.019			0.110
Never	152 (35.1)	210 (34.0)		218 (37.7)	295 (35.0)		235 (35.7)	290 (36.3)	
Former	182 (42.0)	255 (41.3)		259 (44.8)	385 (45.6)		312 (47.3)	365 (45.7)	
Current	99 (22.9)	152 (24.6)		101 (17.5)	164 (19.4)		112 (17.0)	143 (17.9)	
Alcohol drinker (%)	148 (80.4)	308 (71.1)	0.016	207 (77.8)	425 (73.5)	0.182	93 (74.4)	432 (71.9)	0.567
Treatment for									
Hypertension	98 (53.3)	205 (47.3)	0.179	41 (44.1)	395 (52.6)	0.121	79 (56.8)	346 (52.5)	0.352
Diabetes	36 (19.6)	59 (13.6)	0.061	13 (14.0)	135 (18.0)	0.339	34 (24.5)	123 (18.7)	0.118
Parental history (%)	134 (72.8)	319 (73.7)	0.828	69 (25.9)	149 (25.8)	0.960	94 (67.6)	493 (74.8)	0.081

Chekanova V, *et al. BMJ Open* 2023; 13:e065409. doi: 10.1136/bmjopen-2022-065409

CVD risk (%)			<0.001			<0.001			<0.001
Low	32 (17.4)	234 (54.0)		47 (17.7)	319 (55.2)		28 (20.1)	329 (49.9)	
Intermediate	70 (38.0)	114 (26.3)		74 (27.8)	130 (22.5)		36 (25.9)	131 (19.9)	
High	82 (44.6)	85 (19.6)		145 (54.5)	129 (22.3)		75 (54.0)	199 (30.2)	
Statins (%)	161 (87.5)	417 (96.3)	<0.001	200 (75.2)	461 (79.8)	0.134	88 (63.3)	504 (76.5)	0.001
Fibrates (%)	28 (15.2)	17 (3.9)	<0.001	17 (6.4)	16 (2.8)	0.012	7 (5.0)	25 (3.8)	0.497
Other (%)	7 (3.8)	15 (3.5)	0.835	55 (20.7)	96 (16.6)	0.152	46 (33.1)	123 (18.7)	<0.001
Number of drugs									
Including OTC	-	-		4 [3 - 6]	4 [2 - 6]	0.229 §	5 [3 - 7]	5 [3 - 7]	0.842 §
Excluding OTC	-	-		4 [2 - 6]	3 [2 - 5]	0.246 §	4 [2 - 6]	4 [2 - 6]	0.642 §
Genetic risk scores									
Total cholesterol	-2.7 ± 9.4	-3.7 ± 9.6	0.254	-3.7 ± 9.5	-3.5 ± 8.9	0.763	-3.3 ± 9.4	-3.8 ± 8.9	0.562
LDL-cholesterol	-2.4 ± 7.5	-2.9 ± 7.5	0.461	-2.5 ± 7.6	-2.4 ± 7.2	0.847	-2.5 ± 7.3	-2.9 ± 7.0	0.467
HDL-cholesterol	-6.5 ± 3.4	-6.7 ± 3.6	0.649	0.2 ± 7.4	-6.6 ± 3.5	0.312	-6.9 ± 3.8	-6.7 ± 3.6	0.539

BMI, body mass index; CVD, cardio vascular disease; OTC, over the counter. Results are expressed as number of participants (column %) for categorical variables and as average ± standard deviation or as median [interquartile range] for continuous variables. Between-groups comparisons performed using chi-square for categorical variables and student's t-test or Kruskal-Wallis nonparametric test (§) for continuous variables.

Supplemental table 11: multivariable analysis of the factors associated with dyslipidemia control as per the Swiss Group for Lipids and Atherosclerosis criteria. Data from the baseline (2003-2006), first (2009-2012) and second (2014-2017) follow-ups of the CoLaus|PsyCoLaus study, Lausanne, Switzerland.

	**Baseline**		**First follow-up**		**Second follow-up**	
	**OR (95% CI)**	**p-value**	**OR (95% CI)**	**p-value**	**OR (95% CI)**	**p-value**
Age (per 10 years increase)	1.17 (0.90 - 1.51)	0.231	1.05 (0.85 - 1.30)	0.627	1.18 (0.90 - 1.54)	0.221
Man vs. woman	1.70 (1.08 - 2.69)	0.022	1.15 (0.80 - 1.66)	0.458	1.59 (1.00 - 2.53)	0.050
Swiss vs. Non-Swiss	1.26 (0.81 - 1.96)	0.298	1.30 (0.90 - 1.86)	0.159	0.74 (0.45 - 1.21)	0.232
Education						
High	1 (ref.)		1 (ref.)		1 (ref.)	
Middle	1.23 (0.58 - 2.62)	0.586	1.12 (0.64 - 1.96)	0.690	0.44 (0.21 - 0.92)	0.030
Low	1.07 (0.55 - 2.05)	0.848	0.81 (0.49 - 1.32)	0.396	0.61 (0.30 - 1.22)	0.163
p-value for trend	0.848		0.396		0.163	
Married vs. not married	1.11 (0.71 - 1.72)	0.652	1.13 (0.81 - 1.59)	0.466	0.81 (0.52 - 1.26)	0.349
Body mass index categories						
Normal	1 (ref.)		1 (ref.)		1 (ref.)	
Overweight	0.75 (0.45 - 1.25)	0.268	0.92 (0.60 - 1.40)	0.691	0.57 (0.33 - 0.98)	0.043
Obese	1.05 (0.60 - 1.86)	0.854	0.96 (0.59 - 1.54)	0.854	0.58 (0.31 - 1.09)	0.091
p-value for trend	0.854		0.691		0.091	
Smoking categories						
Never	1 (ref.)		1 (ref.)		1 (ref.)	
Former	1.28 (0.78 - 2.08)	0.328	0.91 (0.62 - 1.35)	0.646	1.96 (1.19 - 3.23)	0.008
Current	1.29 (0.73 - 2.26)	0.380	0.91 (0.56 - 1.47)	0.692	1.76 (0.93 - 3.32)	0.081
p-value for trend	0.380		0.692		0.081	
Alcohol drinker (yes vs. no)	0.46 (0.28 - 0.77)	0.003	0.75 (0.50 - 1.12)	0.155	0.74 (0.45 - 1.23)	0.246
Antihypertensive treatment (yes vs. no)	1.50 (0.97 - 2.29)	0.065	1.48 (1.03 - 2.12)	0.036	1.04 (0.64 - 1.69)	0.862
Parental history (yes vs. no)	0.99 (0.61 - 1.59)	0.964	0.96 (0.65 - 1.42)	0.847	0.65 (0.40 - 1.06)	0.086
CVD risk (GSLA criterion)						
Low	1 (ref.)		1 (ref.)		1 (ref.)	

Intermediate	0.16 (0.09 - 0.28)	<0.001	0.19 (0.12 - 0.31)	<0.001	0.22 (0.11 - 0.42)	<0.001
High	0.08 (0.05 - 0.14)	<0.001	0.08 (0.05 - 0.13)	<0.001	0.100 (0.06 - 0.20)	<0.001
p-value for trend	<0.001		<0.001		<0.001	
LDL genetic risk score quartiles						
First	1 (ref.)		1 (ref.)		1 (ref.)	
Second	0.89 (0.50 - 1.58)	0.681	1.07 (0.67 - 1.69)	0.784	1.99 (1.06 - 3.74)	0.033
Third	0.54 (0.31 - 0.96)	0.034	1.11 (0.70 - 1.77)	0.645	0.93 (0.51 - 1.70)	0.815
Fourth	0.74 (0.42 - 1.30)	0.293	1.12 (0.70 - 1.77)	0.641	0.77 (0.43 - 1.38)	0.388
p-value for trend	0.125		0.617		0.108	
Hypolipidemic drug treatment						
Statins	0.35 (0.03 - 4.26)	0.411	1.19 (0.66 - 2.15)	0.570	2.21 (1.14 - 4.28)	0.019
Fibrates	0.06 (0.01 - 0.63)	0.019	0.29 (0.11 - 0.76)	0.012	0.61 (0.2 - 1.86)	0.389
Other	0.80 (0.28 - 2.27)	0.674	0.45 (0.25 - 0.81)	0.008	0.47 (0.25 - 0.89)	0.020

OR, odds ratio; CI, confidence interval. Results are expressed as odds ratio and (95% confidence interval). Statistical analysis using logistic regression.

Supplemental table 12: bivariate comparisons of the statin generation and potency among participants treated for dyslipidemia, according to controlled and uncontrolled status as per the Swiss Group for Lipids and Atherosclerosis criteria. Data from the first (2009-2012) and second (2014-2017) follow-ups of the CoLaus|PsyCoLaus study, Lausanne, Switzerland. Analysis restricted to participants taking statins.

	First follow-up			Second follow-up		
	**Uncontrolled**	**Controlled**	**p-value**	**Uncontrolled**	**Controlled**	**p-value**
N	**200**	**461**		**88**	**502**	
Generation			0.003			0.026
First	62 (31.0)	87 (18.9)		21 (23.9)	65 (13.0)	
Second	122 (61.0)	331 (71.8)		55 (62.5)	366 (72.9)	
Third	16 (8.0)	43 (9.3)		12 (13.6)	71 (14.1)	
Potency §			0.002			0.005
Low	62 (31.0)	87 (18.9)		21 (23.9)	65 (13.0)	
Intermediate	70 (35.0)	169 (36.7)		30 (34.1)	143 (28.5)	
High	68 (34.0)	205 (44.5)		37 (42.1)	294 (58.6)	
N	**186**	**436**		**77**	**442**	
Potency §			0.386			0.199
Low	94 (50.5)	198 (45.4)		34 (44.2)	155 (35.1)	
Intermediate	75 (40.3)	202 (46.3)		28 (36.4)	208 (47.1)	
High	17 (9.1)	36 (8.3)		15 (19.5)	79 (17.9)	

§, not considering posology; †, considering posology. Results are expressed as number of participants (column %). Between-groups comparisons performed using chi-square.

Chekanova V, *et al. BMJ Open* 2023; 13:e065409. doi: 10.1136/bmjopen-2022-065409

Supplemental table 13: multivariable analysis of the factors associated with dyslipidemia control as per the Swiss Group for Lipids and Atherosclerosis criteria. Data from the first (2009-2012) and second (2014-2017) follow-ups of the CoLaus|PsyCoLaus study, Lausanne, Switzerland. Analysis taking into account statin generation.

	**First follow-up**		**Second follow-up**	
	**OR (95% CI)**	**p-value**	**OR (95% CI)**	**p-value**
Age (per 10 years increase)	1.20 (0.93 - 1.56)	0.159	1.04 (0.73 - 1.48)	0.823
Man vs. woman	1.59 (1.03 - 2.45)	0.037	2.23 (1.23 - 4.02)	0.008
Swiss vs. Non-Swiss	1.25 (0.81 - 1.92)	0.315	0.80 (0.43 - 1.47)	0.468
Education				
High	1 (ref.)		1 (ref.)	
Middle	1.09 (0.54 - 2.19)	0.805	0.85 (0.32 - 2.22)	0.733
Low	0.83 (0.46 - 1.52)	0.551	1.13 (0.46 - 2.74)	0.791
p-value for trend	0.551		0.791	
Married vs. not married	1.24 (0.84 - 1.84)	0.287	0.97 (0.55 - 1.71)	0.917
Body mass index categories				
Normal	1 (ref.)		1 (ref.)	
Overweight	0.87 (0.53 - 1.45)	0.603	0.61 (0.29 - 1.28)	0.191
Obese	0.86 (0.49 - 1.50)	0.591	0.47 (0.21 - 1.06)	0.068
p-value for trend	0.591		0.068	
Smoking categories				
Never	1 (ref.)		1 (ref.)	
Former	0.91 (0.58 - 1.44)	0.688	1.79 (0.96 - 3.36)	0.068
Current	0.96 (0.54 - 1.72)	0.891	1.22 (0.56 - 2.66)	0.625
p-value for trend				
Alcohol drinker (yes vs. no)	0.67 (0.41 - 1.08)	0.099	0.95 (0.51 - 1.78)	0.882
AntiHTA ttt (yes vs. no)	1.19 (0.77 - 1.84)	0.438	1.11 (0.60 - 2.05)	0.733
Parental history (yes vs. no)	0.98 (0.61 - 1.58)	0.945	0.54 (0.28 - 1.03)	0.060
CVD risk (GSLA criterion)				
Low	1 (ref.)		1 (ref.)	
Intermediate	0.18 (0.10 - 0.32)	<0.001	0.19 (0.07 - 0.49)	0.001
High	0.05 (0.03 - 0.10)	<0.001	0.06 (0.02 - 0.14)	<0.001
p-value for trend	<0.001		<0.001	
LDL genetic risk score quartiles				
First	1 (ref.)		1 (ref.)	
Second	1.04 (0.61 - 1.76)	0.888	2.95 (1.29 - 6.72)	0.010
Third	1.21 (0.70 - 2.08)	0.492	1.06 (0.51 - 2.19)	0.881
Fourth	1.29 (0.75 - 2.20)	0.359	1.18 (0.57 - 2.47)	0.653
p-value for trend	0.298		0.666	
Number of drugs (per 1 unit)	1.14 (1.05 - 1.25)	0.003	1.16 (1.04 - 1.29)	0.008
Statins generation				
First	1 (ref.)		1 (ref.)	
Second	2.54 (1.61 - 4.01)	<0.001	4.17 (2.07 - 8.42)	<0.001
Third	2.59 (1.19 - 5.61)	0.016	3.44 (1.31 - 8.99)	0.012

Chekanova V, *et al. BMJ Open* 2023; 13:e065409. doi: 10.1136/bmjopen-2022-065409

p-value for trend	0.016		0.012	
Fibrates	NC		NC	
Other	0.62 (0.27 - 1.43)	0.263	1.09 (0.36 - 3.3)	0.880

OR, odds ratio; CI, confidence interval; antiHTA ttt, antihypertensive drug treatment; NC, not computable. Results are expressed as odds ratio and (95% confidence interval). Statistical analysis by logistic regression.

Supplemental table 14: multivariable analysis of the factors associated with dyslipidemia control as per the Swiss Group for Lipids and Atherosclerosis criteria. Data from the first (2009-2012) and second (2014-2017) follow-ups of the CoLaus|PsyCoLaus study, Lausanne, Switzerland. Analysis taking into account statin potency, but not considering posology.

	First follow-up		Second follow-up	
	**OR (95% CI)**	**p-value**	**OR (95% CI)**	**p-value**
Age (per 10 years increase)	1.22 (0.94 - 1.59)	0.127	1.06 (0.74 - 1.51)	0.748
Man vs. woman	1.57 (1.02 - 2.43)	0.040	2.13 (1.17 - 3.87)	0.013
Swiss vs. Non-Swiss	1.24 (0.81 - 1.92)	0.322	0.82 (0.45 - 1.52)	0.533
Education				
High	1 (ref.)		1 (ref.)	
Middle	1.07 (0.53 - 2.14)	0.856	0.77 (0.29 - 2.04)	0.601
Low	0.83 (0.45 - 1.50)	0.531	1.05 (0.43 - 2.58)	0.914
p-value for trend	0.531		0.914	
Married vs. not married	1.26 (0.85 - 1.86)	0.259	1.00 (0.57 - 1.76)	0.995
Body mass index categories				
Normal	1 (ref.)		1 (ref.)	
Overweight	0.89 (0.54 - 1.47)	0.637	0.64 (0.31 - 1.35)	0.245
Obese	0.85 (0.49 - 1.49)	0.570	0.49 (0.22 - 1.10)	0.085
p-value for trend	0.570		0.085	
Smoking categories				
Never	1 (ref.)		1 (ref.)	
Former	0.90 (0.57 - 1.42)	0.653	1.83 (0.97 - 3.46)	0.061
Current	0.96 (0.54 - 1.72)	0.895	1.18 (0.53 - 2.58)	0.688
p-value for trend	0.895		0.688	
Alcohol drinker (yes vs. no)	0.67 (0.42 - 1.09)	0.106	0.90 (0.48 - 1.70)	0.750
AntiHTA ttt (yes vs. no)	1.20 (0.77 - 1.85)	0.417	1.09 (0.59 - 2.02)	0.781
Parental history (yes vs. no)	0.98 (0.61 - 1.58)	0.942	0.52 (0.27 - 0.99)	0.049
CVD risk				
Low	1 (ref.)		1 (ref.)	
Intermediate	0.17 (0.10 - 0.31)	<0.001	0.20 (0.08 - 0.51)	0.001
High	0.05 (0.03 - 0.10)	<0.001	0.06 (0.02 - 0.15)	<0.001
p-value for trend	<0.001		<0.001	
LDL genetic risk score quartiles				
First	1 (ref.)		1 (ref.)	
Second	1.05 (0.62 - 1.79)	0.852	2.74 (1.20 - 6.24)	0.016
Third	1.19 (0.70 - 2.05)	0.519	1.01 (0.49 - 2.10)	0.982
Fourth	1.27 (0.74 - 2.18)	0.380	1.12 (0.53 - 2.35)	0.763
p-value for trend	0.329		0.586	
Number of drugs (per 1 unit)	1.14 (1.04 - 1.25)	0.003	1.15 (1.03 - 1.28)	0.012
Statins potency, no posology				
Low	1 (ref.)		1 (ref.)	
Intermediate	2.14 (1.30 - 3.54)	0.003	2.90 (1.35 - 6.24)	0.007
High	3.01 (1.82 - 4.98)	<0.001	5.13 (2.43 - 10.8)	<0.001

p-value for trend	<0.001		<0.001	
Fibrates	NC		NC	
Other	0.58 (0.25 - 1.34)	0.202	0.91 (0.30 - 2.76)	0.871

OR, odds ratio; CI, confidence interval; antiHTA ttt, antihypertensive drug treatment; NC, not computable. Results are expressed as odds ratio and (95% confidence interval). Statistical analysis

Supplemental table 15: multivariable analysis of the factors associated with dyslipidemia control as per the Swiss Group for Lipids and Atherosclerosis criteria. Data from the first (2009-2012) and second (2014-2017) follow-ups of the CoLaus|PsyCoLaus study, Lausanne, Switzerland. Analysis taking into account statin potency and posology

	**First follow-up**		**Second follow-up**	
	**OR (95% CI)**	**p-value**	**OR (95% CI)**	**p-value**
Age (per 10 years increase)	1.25 (0.96 - 1.63)	0.102	1.10 (0.76 - 1.60)	0.619
Man vs. woman	1.61 (1.04 - 2.51)	0.034	1.82 (0.97 - 3.42)	0.062
Swiss vs. Non-Swiss	1.25 (0.80 - 1.94)	0.321	0.81 (0.42 - 1.54)	0.515
Education				
High	1 (ref.)		1 (ref.)	
Middle	1.08 (0.53 - 2.19)	0.839	0.47 (0.16 - 1.40)	0.175
Low	0.80 (0.43 - 1.46)	0.465	0.65 (0.23 - 1.79)	0.402
p-value for trend	0.465		0.402	
Married vs. not married	1.33 (0.89 - 2.00)	0.163	1.06 (0.59 - 1.92)	0.842
Body mass index categories				
Normal	1 (ref.)		1 (ref.)	
Overweight	0.86 (0.51 - 1.43)	0.554	0.66 (0.31 - 1.40)	0.283
Obese	0.87 (0.49 - 1.55)	0.641	0.65 (0.28 - 1.48)	0.302
p-value for trend	0.641		0.302	
Smoking categories				
Never	1 (ref.)		1 (ref.)	
Former	0.95 (0.60 - 1.52)	0.836	1.72 (0.89 - 3.34)	0.109
Current	1.05 (0.58 - 1.89)	0.868	1.20 (0.52 - 2.78)	0.672
p-value for trend	0.868		0.672	
Alcohol drinker (yes vs. no)	0.67 (0.41 - 1.10)	0.113	0.93 (0.48 - 1.8)	0.831
AntiHTA ttt (yes vs. no)	1.35 (0.86 - 2.11)	0.187	1.20 (0.63 - 2.28)	0.575
Parental history (yes vs. no)	0.94 (0.58 - 1.52)	0.805	0.67 (0.34 - 1.33)	0.251
CVD risk				
Low	1 (ref.)		1 (ref.)	
Intermediate	0.19 (0.10 - 0.34)	<0.001	0.23 (0.09 - 0.59)	0.002
High	0.06 (0.03 - 0.11)	<0.001	0.07 (0.03 - 0.18)	<0.001
p-value for trend	<0.001		<0.001	
LDL genetic score quartiles				
First	1 (ref.)		1 (ref.)	
Second	1.07 (0.62 - 1.85)	0.800	1.89 (0.81 - 4.39)	0.141
Third	1.34 (0.77 - 2.34)	0.299	0.83 (0.38 - 1.79)	0.634
Fourth	1.25 (0.72 - 2.17)	0.422	1.00 (0.46 - 2.17)	0.997
p-value for trend	0.313		0.514	
Number of drugs (per 1 unit)	1.11 (1.01 - 1.21)	0.028	1.16 (1.02 - 1.30)	0.019
Statins potency, posology				
Low	1 (ref.)		1 (ref.)	
Intermediate	1.36 (0.91 - 2.06)	0.137	1.66 (0.88 - 3.11)	0.115
High	1.67 (0.81 - 3.45)	0.168	2.07 (0.89 - 4.82)	0.092

p-value for trend	0.168		0.092	
Fibrates	NC		NC	
Other	0.75 (0.31 - 1.84)	0.529	1.64 (0.43 - 6.28)	0.473

OR, odds ratio; CI, confidence interval; antiHTA ttt, antihypertensive drug treatment; NC, not computable. Results are expressed as odds ratio and (95% confidence interval). Statistical analysis by logistic regression.

Chekanova V, *et al. BMJ Open* 2023; 13:e065409. doi: 10.1136/bmjopen-2022-065409

Supplemental table 16: p-values for the different SNPs associated with statin effect regarding dyslipidemia control, according to the European Society of Cardiology / European Atherosclerosis Society criteria, and the Swiss Group for Lipids and Atherosclerosis strict and near control criteria. Data from the baseline (2003-2006), first (2009-2012) and second (2014-2017) follow-ups of the CoLaus|PsyCoLaus study, Lausanne, Switzerland.

			ESC/EAS			GSLA strict control			GSLA near control	
**Gene**	**SNP**	**Baseline**	**1st FU**	**2nd FU**	**Baseline**	**First FU**	**2nd FU**	**Baseline**	**First FU**	**2nd FU**
ABCB1	rs1128503	0.728	0.896	**0.040**	0.969	0.794	0.167	0.481	0.539	0.513
ABCB1	rs1922242	0.910	0.369	0.147	0.648	0.360	**0.026**	0.902	0.598	0.474
ABCB1	rs2032582	0.835	0.492	0.156	0.887	0.673	0.150	0.387	0.553	0.454
ABCB1	rs3789244	0.728	0.901	**0.040**	0.985	0.779	0.167	0.481	0.539	0.513
ABCG2/BCRP	rs2199936	0.485	0.861	0.444	0.649	0.381	0.141	0.452	0.295	0.507
ABCG2/BCRP	rs2231137	0.094	0.966	0.292	1.000	0.817	0.906	0.671	0.897	0.584
ABCG2/BCRP	rs2231142	0.411	0.894	0.444	0.592	0.310	0.141	0.452	0.329	0.507
ABCG8	rs11887534	0.541	0.671	**0.002**	0.945	0.210	0.196	1.000	**0.031**	0.603
APOA1	rs5069	0.232	0.858	1.000	0.072	0.245	1.000	0.501	0.219	0.680
APOA1	rs670	0.793	0.314	0.125	0.735	0.378	0.386	0.456	0.372	0.833
APOA5	rs662799	0.238	0.052	0.092	0.712	0.792	0.125	0.396	0.437	1.000
APOC1	rs4420638	0.847	0.089	0.567	0.514	0.697	0.085	0.092	0.208	0.503
APOE	rs429358	1.000	0.077	0.502	0.308	0.576	0.784	0.105	1.000	1.000
APOE	rs445925	0.718	0.623	0.788	1.000	0.141	0.856	0.414	0.606	1.000
APOE	rs449647	0.461	0.888	0.207	0.919	0.870	0.617	0.708	0.642	1.000
APOE	rs71352238	0.856	0.269	0.489	0.358	0.952	0.642	0.056	0.761	0.791
APOE	rs7412	0.246	0.154	0.145	0.792	0.694	0.803	0.702	0.347	1.000
CETP	rs5882	0.683	0.749	0.932	0.446	0.773	0.211	0.371	0.105	0.319
CETP	rs708272	0.088	0.841	0.459	0.593	0.580	0.247	0.785	0.182	0.453
CLMN	rs8014194	0.304	0.477	0.382	0.339	0.543	0.636	0.138	0.154	0.527
CYP2C9	rs1057910	0.798	0.064	0.060	0.392	0.060	0.163	0.393	0.064	0.302
CYP2D6	rs3892097	0.781	0.540	0.800	0.328	0.641	0.601	0.619	0.798	0.242
CYP3A4	rs2740574	0.394	0.305	0.430	0.805	0.729	0.220	0.165	0.473	0.703

CYP3A4	rs55951658	NC	NC	NC	NC	NC	NC	NC	NC	NC
CYP7A1	rs3808607	**0.043**	0.573	0.267	**0.017**	0.153	0.601	0.647	0.149	0.766
ESR1	rs2234693	**0.026**	0.938	1.000	0.613	0.868	0.119	0.816	0.797	0.757
ESR1	rs9340799	0.139	0.899	0.569	0.420	0.778	0.156	**0.036**	0.390	0.619
HMGCR	rs12916	0.598	0.406	0.626	0.584	0.912	0.651	0.802	0.139	0.413
HMGCR	rs17238540	0.833	0.552	0.713	0.153	0.901	0.537	1.000	0.450	0.430
HMGCR	rs17244841	0.666	0.498	0.705	0.151	1.000	0.536	1.000	0.442	0.424
HMGCR	rs17671591	0.171	0.947	0.158	0.337	0.388	0.634	0.590	0.124	0.737
HMGCR	rs5908	0.102	0.507	0.632	1.000	1.000	0.466	0.271	1.000	1.000
KIF6	rs20455	0.307	**0.029**	0.209	0.602	0.323	0.609	0.094	0.124	0.058
LDLR	rs5925	0.408	0.311	**0.015**	0.206	0.281	0.348	0.553	0.889	0.935
LPA	rs10455872	0.266	0.659	0.332	**0.021**	0.340	1.000	1.000	0.461	0.452
LPL	rs328	0.372	0.397	0.674	0.186	0.654	0.835	0.087	1.000	0.878
PCSK9	rs17111584	0.462	0.359	0.054	1.000	0.197	1.000	0.380	**0.027**	1.000
PON1	rs662	0.069	0.637	0.911	0.130	0.407	0.684	0.730	1.000	0.749
SCAP	rs12487736	**0.031**	0.145	0.312	0.226	0.525	0.840	**0.032**	0.711	0.413
SCCO1B1	rs2306283	0.076	0.429	0.384	0.533	0.404	0.902	0.719	0.557	0.126
SLCO1B1	rs11045819	0.914	0.768	0.068	**0.020**	0.841	0.250	0.466	**0.044**	0.728
SLCO1B1	rs4149015	0.572	0.819	0.808	0.895	0.084	0.303	0.758	0.438	**0.039**
SLCO1B1	rs4149056	0.112	0.888	**0.011**	0.675	0.574	**0.027**	0.856	**0.045**	0.356
SOD2	rs4880	0.255	0.611	0.460	0.182	0.740	0.837	0.228	0.925	0.214
TLR4	rs4986790	0.654	0.873	**0.013**	0.727	0.124	0.662	1.000	0.166	1.000
ABCB1	rs1045642	0.789	0.500	0.619	0.877	0.160	0.350	0.461	0.578	0.269

NC, not computable as all participants were homozygous. FU, follow-up. Statistical analysis performed using Fisher's exact test. Values <0.05 are indicated in bold.

Chekanova V, *et al. BMJ Open* 2023; 13:e065409. doi: 10.1136/bmjopen-2022-065409

References

1. Maji D, Shaikh S, Solanki D, et al. Safety of statins. *Indian J Endocrinol Metab* 2013; 17: 636-646. 2013/08/21. DOI: 10.4103/2230-8210.113754.
2. Stone NJ, Robinson JG, Lichtenstein AH, et al. 2013 ACC/AHA guideline on the treatment of blood cholesterol to reduce atherosclerotic cardiovascular risk in adults: a report of the American College of Cardiology/American Heart Association Task Force on Practice Guidelines. *J Am Coll Cardiol* 2014; 63: 2889-2934. 2013/11/19. DOI: 10.1016/j.jacc.2013.11.002.
3. Catapano AL, Graham I, De Backer G, et al. 2016 ESC/EAS Guidelines for the Management of Dyslipidaemias. *Eur Heart J* 2016; 37: 2999-3058. 2016/08/28. DOI: 10.1093/eurheartj/ehw272.
4. Marques-Vidal P, Rodondi N, Bochud M, et al. Predictive accuracy and usefulness of calibration of the ESC SCORE in Switzerland. *Eur J Cardiovasc Prev Rehabil* 2008; 15: 402-408. 2008/08/05. DOI: 10.1097/HJR.0b013e3282fb040f.
5. Firmann M, Marques-Vidal P, Paccaud F, et al. Prevalence, treatment and control of dyslipidaemia in Switzerland: still a long way to go. *Eur J Cardiovasc Prev Rehabil* 2010; 17: 682-687. 2010/08/12. DOI: 10.1097/HJR.0b013e32833a09ab.
6. Maxwell WD, Ramsey LB, Johnson SG, et al. Impact of pharmacogenetics on efficacy and safety of statin therapy for dyslipidemia. *Pharmacotherapy* 2017; 37: 1172-1190. 2017/07/04. DOI: 10.1002/phar.1981.
7. Spracklen CN, Saftlas AF, Triche EW, et al. Genetic Predisposition to Dyslipidemia and Risk of Preeclampsia. *Am J Hypertens* 2015; 28: 915-923. 2014/12/20. DOI: 10.1093/ajh/hpu242.

Chekanova V, *et al. BMJ Open* 2023; 13:e065409. doi: 10.1136/bmjopen-2022-065409
