# Supplementary material for: Association of clinical and genetic risk factors with management of dyslipidaemia: analysis of repeated cross-sectional studies in the general population of Lausanne, Switzerland
Source: BMJ Open. 2023 Feb 21;13(2):e065409. doi: 10.1136/bmjopen-2022-065409 (PMC9945309; doi:10.1136/bmjopen-2022-065409)

BMJ Publishing Group Limited (BMJ) disclaims all liability and responsibility arising from any reliance placed on this supplemental material which has been supplied by the author(s)

Supplemental figure 2: distribution of hypolipidemic drug classes, baseline (2003-2006), first (2009-2012) and second (2014-2017) follow-ups of the CoLaus|PsyCoLaus study, Lausanne, Switzerland

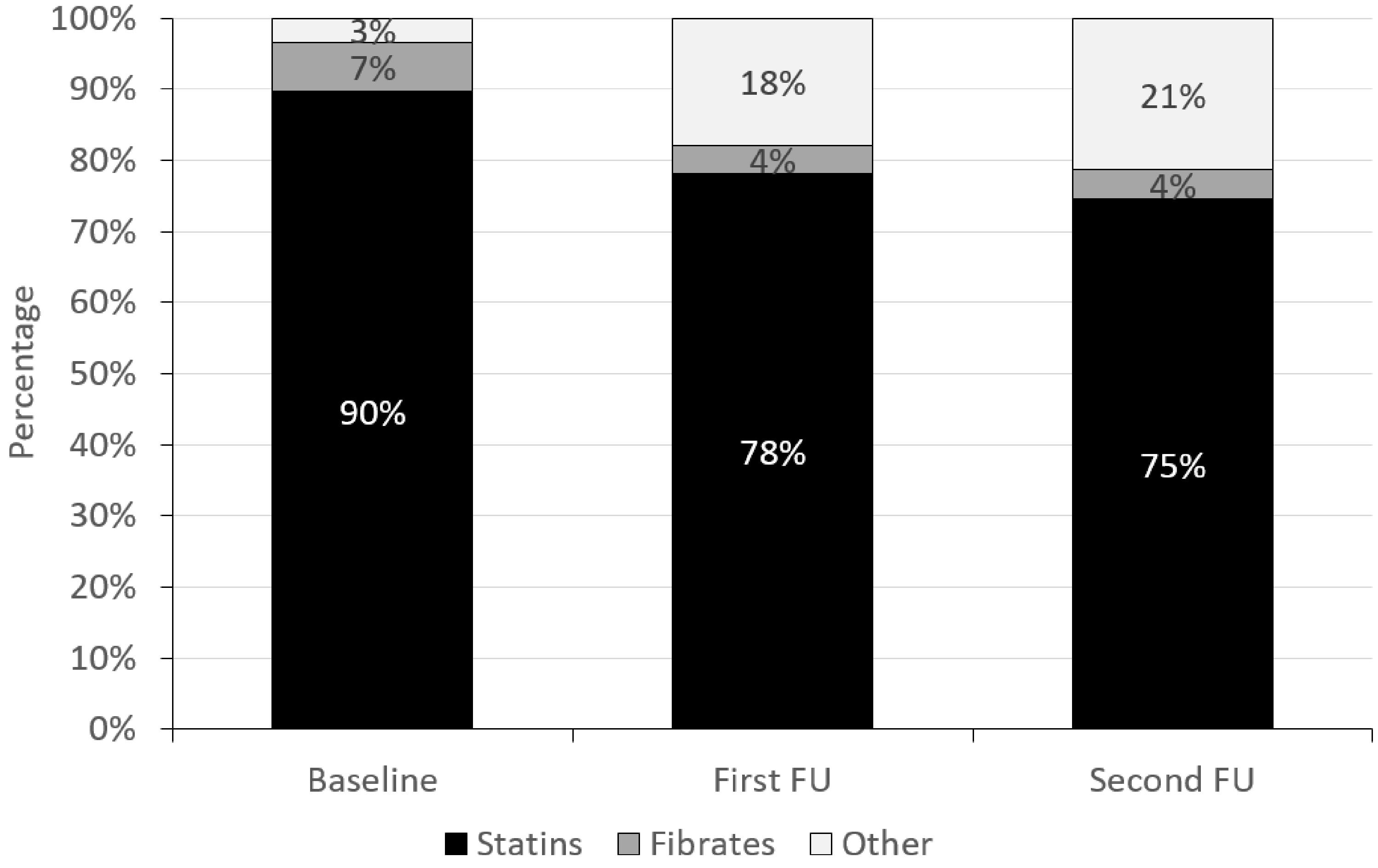

Supplement: Supplementary data [file bmjopen-2022-065409supp003.pdf]
