# Supplementary material for: Association of clinical and genetic risk factors with management of dyslipidaemia: analysis of repeated cross-sectional studies in the general population of Lausanne, Switzerland
Source: BMJ Open. 2023 Feb 21;13(2):e065409. doi: 10.1136/bmjopen-2022-065409 (PMC9945309; doi:10.1136/bmjopen-2022-065409)

Supplemental figure 3: Generation and potency of statin drugs, first (2009-2012) and second (2014-2017) follow-ups of the CoLaus|PsyCoLaus study, Lausanne, Switzerland. Black: first generation or low potency; dark gray, second generation or intermediate potency; light gray, third generation or high potency.

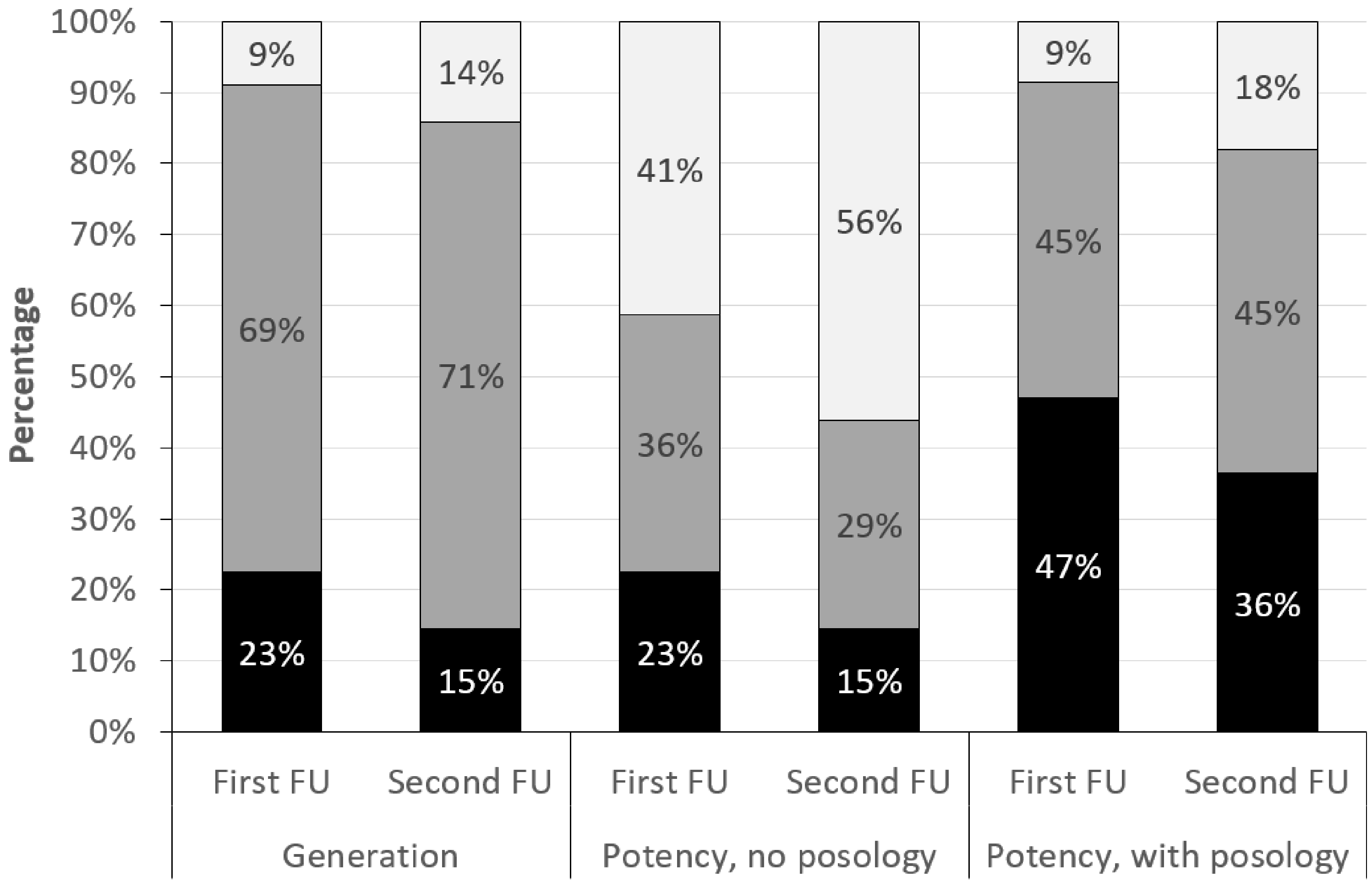

Supplement: Supplementary data [file bmjopen-2022-065409supp004.pdf]
