# Supplementary material for: Association of clinical and genetic risk factors with management of dyslipidaemia: analysis of repeated cross-sectional studies in the general population of Lausanne, Switzerland
Source: BMJ Open. 2023 Feb 21;13(2):e065409. doi: 10.1136/bmjopen-2022-065409 (PMC9945309; doi:10.1136/bmjopen-2022-065409)
Supplement: Supplementary data [file bmjopen-2022-065409supp005.pdf]

Supplemental figure 4: comparison of statin potency considering or not posology, first (2009-2012) and second (2014-2017) follow-ups of the CoLaus|PsyCoLaus study, Lausanne, Switzerland.

First follow-up

		With posology Low	Intermediate	High
	Low	85	87	0
Without posology	Intermediate	283	7	0
	High	0	256	70

Second follow-up

		With posology Low	Intermediate	High
	Low	67	43	0
Without posology	Intermediate	197	8	0
	High	0	282	136
