# Supplementary material for: Association of clinical and genetic risk factors with management of dyslipidaemia: analysis of repeated cross-sectional studies in the general population of Lausanne, Switzerland
Source: BMJ Open. 2023 Feb 21;13(2):e065409. doi: 10.1136/bmjopen-2022-065409 (PMC9945309; doi:10.1136/bmjopen-2022-065409)
Supplement: Author's manuscript [file bmjopen-2022-065409.draft_revisions.pdf]

BMJ Open

BMJ Open is committed to open peer review. As part of this commitment we make the peer review history of every article we publish publicly available.

When an article is published we post the peer reviewers' comments and the authors' responses online. We also post the versions of the paper that were used during peer review. These are the versions that the peer review comments apply to.

The versions of the paper that follow are the versions that were submitted during the peer review process. They are not the versions of record or the final published versions. They should not be cited or distributed as the published version of this manuscript.

BMJ Open is an open access journal and the full, final, typeset and author-corrected version of record of the manuscript is available on our site with no access controls, subscription charges or pay-per-view fees (http://bmjopen.bmj.com).

If you have any questions on BMJ Open's open peer review process please email info.bmjopen@bmj.com

BMJ Open

Genetic factors are not associated with management of dyslipidemia in the general population

Journal:	*BMJ Open*
Manuscript ID	bmjopen-2022-065409
Article Type:	Original research
Date Submitted by the Author:	07-Jun-2022

Complete List of Authors:	Chekanova, Valeriya; National Medical Research Center of Cardiology; University of Lausanne, Department of Medicine, Internal medicine<br>Abolhassani, Nazanin; University of Lausanne, Department of Medicine, Internal medicine; Institute of Primary health care<br>Vaucher, Julien; CHUV, Medicine, Internal medicine<br>Marques-Vidal, Pedro; Centre hospitalier universitaire vaudois Departement de medecine, Department of Medicine, Internal Medicine

Keywords:	Lipid disorders < DIABETES & ENDOCRINOLOGY, EPIDEMIOLOGY, GENETICS, CARDIOLOGY

SCHOLARONE™
Manuscripts

I, the Submitting Author has the right to grant and does grant on behalf of all authors of the Work (as defined in the below author licence), an exclusive licence and/or a non-exclusive licence for contributions from authors who are: i) UK Crown employees; ii) where BMJ has agreed a CC-BY licence shall apply, and/or iii) in accordance with the terms applicable for US Federal Government officers or employees acting as part of their official duties; on a worldwide, perpetual, irrevocable, royalty-free basis to BMJ Publishing Group Ltd ("BMJ") its licensees and where the relevant Journal is co-owned by BMJ to the co-owners of the Journal, to publish the Work in this journal and any other BMJ products and to exploit all rights, as set out in our licence.

The Submitting Author accepts and understands that any supply made under these terms is made by BMJ to the Submitting Author unless you are acting as an employee on behalf of your employer or a postgraduate student of an affiliated institution which is paying any applicable article publishing charge ("APC") for Open Access articles. Where the Submitting Author wishes to make the Work available on an Open Access basis (and intends to pay the relevant APC), the terms of reuse of such Open Access shall be governed by a Creative Commons licence – details of these licences and which Creative Commons licence will apply to this Work are set out in our licence referred to above.

Other than as permitted in any relevant BMJ Author's Self Archiving Policies, I confirm this Work has not been accepted for publication elsewhere, is not being considered for publication elsewhere and does not duplicate material already published. I confirm all authors consent to publication of this Work and authorise the granting of this licence.

For peer review only

Genetic factors are not associated with management of dyslipidemia in the general population

Short title: no effect of genetic risk scores on dyslipidemia control

Valeriya Chekanova, MD [1, 2]; Nazanin Abolhassani, PhD [2, 3]; Julien Vaucher, MD, PhD [2] and Pedro Marques-Vidal, MD, PhD [2].

[1] National Medical Research Center of Cardiology, Moscow, Russia; [2] Department of Medicine, Internal medicine, Lausanne university hospital and university of Lausanne, Lausanne, Switzerland; [3] Institute of Primary health care (BIHAM), Bern, Switzerland.

Authors' emails

Valeriya Chekanova: 2466107@mail.ru

Nazanin Abolhassani: Nazanin.Abolhassani@biham.unibe.ch

Julien Vaucher: Julien.Vaucher@chuv.ch

Pedro Marques-Vidal: Pedro.marquesvidal@gmail.com

Address for correspondence and reprints

Pedro Marques-Vidal

Office BH10-642

Internal Medicine, Lausanne University Hospital (CHUV)

Rue du Bugnon 46

1011 Lausanne

Switzerland

Phone: +41 21 314 09 34

Email: Pedro-Manuel.Marques-Vidal@chuv.ch or Pedro.marquesvidal@gmail.com

For peer review only

Tweet text: when managing dyslipidemia, focus should be on clinical data and statin potency, not on genetic scores.

Word count: abstract 230, main text 2980, 5 tables, 29 references

For peer review only - http://bmjopen.bmj.com/site/about/guidelines.xhtml

Abstract

Objective: To assess the importance of clinical and genetic factors in management of dyslipidemia in the general population.

Methods: Data from baseline (2003-2006), first (2009-2012) and second (2014-2017) follow-ups of a population-based, prospective study conducted in Lausanne, Switzerland. Management of dyslipidemia was assessed according to European or Swiss guidelines. GRS for lipid levels were computed based on the existing literature.

Results: 617, 844 and 798 participants of the baseline, first and second follow-ups were included. Prevalence of adequately controlled dyslipidemia was 52%, 45% and 46% at baseline, first and second follow-ups, respectively. On multivariable analysis, when compared to intermediate or low-risk individuals, participants at very high cardiovascular risk had odds ratio (OR) for dyslipidemia control of 0.11 (95% confidence interval, 0.06-0.18), 0.12 (0.08-0.19) and 0.38 (0.25-0.59) at baseline, first and second follow-ups, respectively. Use of newer generation or higher potency statins was associated with better control: OR 1.90 (1.18-3.05) and 3.62 (1.65-7.92) for second and third generations compared to first in the first follow-up, the corresponding values in the second follow-up being 1.90 (1.08-3.36) and 2.18 (1.05-4.51). No differences in GRS were found between controlled and inadequately controlled subjects. Similar findings were obtained using Swiss guidelines to manage dyslipidemia.

Conclusion: Management of dyslipidemia is suboptimal in Switzerland. The effectiveness of high potency statins is hampered by low posology. The use of GRS in the management of dyslipidemia is not recommended.

Keywords: dyslipidemia, management and control; genetic risk score.

For peer review only - http://bmjopen.bmj.com/site/about/guidelines.xhtml

What is already known on this topic

Management of dyslipidemia in the general population is suboptimal, and a recent review suggested that several single nucleotide polymorphisms (SNPs) could be associated with a reduction in the efficacy of statin treatment. Still, the importance of genetic risk scores (GRS) in management of dyslipidemia is unclear

What this study adds

GRS for plasma lipids were not associated with dyslipidemia control.

How this study might affect research, practice or policy

Our results suggest that clinicians should not use GRS for dyslipidemia management and should focus on assessing CV risk and providing the most potent hypolipidemic treatment possible.

For peer review only

For peer review only - http://bmjopen.bmj.com/site/about/guidelines.xhtml

Introduction

Adequate management of dyslipidemia (high LDL cholesterol levels) translates into a reduction in fatal and non-fatal cardiovascular disease (CVD) [1, 2], and guidelines for the management of dyslipidemia have been issued by the European Society of Cardiology (ESC) and the European Atherosclerosis Society (EAS) [3]. Potent hypolipidemic drugs are available, allowing a considerable reduction in LDL-cholesterol levels [3]. Still, management of dyslipidemia is suboptimal, with a significant percentage of treated patients not reaching target levels [4]. Likely contributing factors are inadequate perception of risk by physicians [5], low compliance by patients [6] or use of lesser potent drugs [4]. It has also been suggested that the efficacy of statins, the main hypolipidemic drugs used, could be modulated by the genetic background of the patients [7, 8]. A recent review suggested that several single nucleotide polymorphisms (SNPs) could be associated with a reduction in the efficacy of statin treatment [8]. Still, the effect of genetic markers on the management of dyslipidemia in the general population has seldom been established.

Thus, we aimed to assess the importance of clinical and genetic factors in the management of dyslipidemia using data from a population-based cohort.

Methods

Study population

The CoLaus|PsyCoLaus (www.colaus-psycolaus.ch) is a prospective cohort study following every 5 years a sample of the inhabitants of the city of Lausanne (Switzerland), aged 35 to 75 years at baseline [9]. In this study, data from the baseline (2003-2006), the first (2009-2012) and the second (2014-2017) follow-ups were used.

Inclusion and exclusion criteria

Participants were eligible if they received any type of lipid-lowering drug. Participants were initially excluded if they had missing information regarding lipid levels, covariates, or genetic data.

For peer review only - http://bmjopen.bmj.com/site/about/guidelines.xhtml

Lipid-lowering treatment and control of dyslipidemia

At each survey, participants reported which drugs they were taking. Based on the Anatomical Therapeutic Chemical (ATC) classification system of the WHO, participants were considered as being treated for dyslipidemia if they were taking one drug coded C10 ("Lipid modifying agents"). Lipid-lowering drugs were further classified into statins, fibrates, and other lipid-lowering drugs. For statins, a further classification regarding the generation and potency was performed in the first and second follow-ups (**supplemental table 1**). Such classification could not be achieved in the baseline survey due to limited coding. Two approaches regarding statin potency were conducted: 1) not taking into account and 2) taking into account posology as defined by US guidelines [10]. This last approach is similar to another study conducted in Poland [11].

As there is no consensus regarding CVD risk assessment in Switzerland, two approaches were applied. The first approach used the ESC/EAS guidelines [3] by applying the SCORE equation recalibrated for Switzerland [12] (**supplemental table 2**). Three CVD categories were defined: very high, high and other. The second approach used the Swiss Group for Lipids and Atherosclerosis (GSLA) criteria [13] (**supplemental table 3**). Depending on the risk category, the threshold to define adequate control changed (**supplemental tables 2 and 3**).

Genetic analysis and genetic scores

Genome-wide genotyping was performed using the Affymetrix 500K SNP array. Subjects were excluded from the analysis in case of inconsistency between sex and genetic data, a genotype call rate of less than 90%, or inconsistencies of genotyping results in duplicate samples. Quality control for SNPs was performed using the following criteria: monomorphic (or with minor allele frequency (MAF) <1%), call rates less than 90%, deviation from the Hardy-Weinberg equilibrium (HWE) ($p<1x10^{-6}$). Phased haplotypes were generated using SHAPEIT2 [14]. Imputation was performed using minimac3 and the Haplotype Reference Consortium version r1.1. Fifty-one SNPs associated with lipid-lowering drug efficiency were extracted (**supplemental table 4**) from a previous review [8]. Genetic risk scores (GRS) for total, LDL- and HDL-cholesterol were computed using 223 SNPs overall as suggested previously

For peer review only - http://bmjopen.bmj.com/site/about/guidelines.xhtml

[15]. Briefly, the GRS were calculated with each SNP being weighted by its relative effect size (β coefficient) obtained from the literature (**supplemental table 5**).

Other covariates

Socio-demographic and lifestyle data were collected by questionnaire and included gender, age, educational level (low/middle/high), marital status (alone/couple), personal and family history of CVD, family history of dyslipidemia, smoking (never/former/current) and alcohol consumption (yes/no). Number of other drugs (including or excluding non-prescribed, over-the-counter drugs) was considered as a proxy for the number of comorbidities.

Body weight and height were measured with participants barefoot and in light indoor clothes. Body weight was measured in kilograms to the nearest 100 g using a Seca® scale (Hamburg, Germany). Height was measured to the nearest 5 mm using a Seca® (Hamburg, Germany) height gauge. Body mass index (BMI) was calculated and categorized into normal (<25 kg/m^2), overweight (25≤BMI<30 kg/m^2) and obese (BMI≥30 kg/m^2).

Blood pressure (BP) was measured using an Omron® HEM-907 automated oscillometric sphygmomanometer after at least a 10-minute rest in a seated position, and the average of the last two measurements was used. Hypertension was defined by a systolic blood pressure (SBP) ≥140 mm Hg or a diastolic blood pressure (DBP) ≥90 mm Hg or presence of antihypertensive drug treatment.

Eight-hour fasting blood samples were collected, and biological measurements were conducted in a Modular P apparatus (Roche Diagnostics, Basel, Switzerland) for the baseline and first follow-up, and in a Cobas 8000 (Roche Diagnostics, Basel, Switzerland) device for the second follow-up. The following analytical procedures (with maximum inter and intra-batch CVs) were used: total cholesterol by CHOD-PAP (1.6-1.7%) and HDL-cholesterol by CHOD-PAP + PEG + cyclodextrin (3.6-0.9%). Glucose was assessed by glucose dehydrogenase (2.1-1.0%) at baseline and by glucose hexokinase (1.6-0.8%) at first and second follow-ups. Diabetes was defined as fasting plasma glucose ≥7.0 mmol/L or presence of an antidiabetic drug treatment.

For peer review only - http://bmjopen.bmj.com/site/about/guidelines.xhtml

Statistical analysis

Statistical analyses were conducted using Stata v.16.1 (Stata Corp, College Station, TX, USA) separately for each survey. Results were expressed as number of participants (percentage) for categorical variables and as average±standard deviation or median [interquartile range] for continuous variables. Bivariate comparisons between controlled and uncontrolled participants (using either ESC/EAS or GSLA criteria) were performed using chi-square for categorical variables and Student's t-test or Kruskal-Wallis nonparametric test for continuous variables. Multivariable analyses were conducted using logistic regression for categorical variables and results were expressed as multivariable-adjusted odds ratio (OR) and 95% confidence interval (CI).

The associations between specific SNPs and management of dyslipidemia were assessed by comparing the distribution of the genotypes according to controlled and uncontrolled participants (as defined by ESC/EAS or GSLA criteria) using Fisher's exact test.

Statistical significance was considered for a two-sided test with $p<0.05$.

Patient and public involvement

The design and conduction of the CoLaus|PsyCoLaus study did not involve patients or the public as this was not a requirement when the study was initiated.

Results

Prevalence of dyslipidemia and changes in statin category

Overall, there were 709, 1056 and 1151 eligible participants at baseline, first and second follow-ups, respectively, of whom 92 (13.0%), 212 (20.1%) and 353 (30.7%) were excluded, leaving 617, 844 and 798 participants for analysis. The reasons for exclusion are indicated in **supplemental figure 1**; the main reason was lack of genetic data. The characteristics of the included and the excluded participants are summarized in **supplemental table 6**; excluded participants were less frequently born in Switzerland, while no other consistent difference was found.

For peer review only - http://bmjopen.bmj.com/site/about/guidelines.xhtml

The distribution of the different types of lipid-lowering treatments for the three surveys is provided in **supplemental figure 2**, and of the statin generations and potency for the first and second follow-ups are provided in **supplemental figure 3**. Statins represented the first type of hypolipidemic drug, but their predominance decreased with time. Prevalence of first generation statins decreased and prevalence of third generation statins increased. Prevalence of low potency statins decreased and high potency statins increased. When posology was considered, statin potency was considerably reduced, but trends were similar (**supplemental figure 3**). This decrease in potency was most marked for intermediate potency statins (**supplemental figure 4**).

Prevalence and factors associated with control of dyslipidemia, ESC/EAS criteria

Prevalence of adequately managed dyslipidemia was 52%, 45% and 46% at baseline, first and second follow-ups, respectively. The results of the analysis using the ESC/EAS criteria stratified by survey are summarized in **tables 1 to 4**.

On bivariate analysis (**table 1**), controlled participants were younger, had lower levels of cardiovascular risk factors and CVD risk, and a higher prevalence of parental history of CVD than inadequately controlled participants in all surveys. Controlled participants also had a lower BMI and were taking less drugs than inadequately controlled participants in the first and second follow-ups; prevalence of fibrates was higher among inadequately controlled participants at baseline and in the first follow-up. No differences were found regarding GRS between controlled and inadequately controlled participants in all surveys. On multivariable analysis (**table 2**), increased age or CVD risk was negatively associated with control in all surveys; no association was found between type of hylipolipidemic drug or quartiles of the LDL GRS and dyslipidemia control.

For peer review only - http://bmjopen.bmj.com/site/about/guidelines.xhtml

Table 1: bivariate comparison between controlled and uncontrolled participants, using ESC/EAS criteria, baseline (2003-2006), first (2009-2012) and second (2014-2017) follow-ups of the CoLaus|PsyCoLaus study, Lausanne, Switzerland.

	Baseline			First follow-up			Second follow-up		
	Uncontrolled	Controlled	p-value	Uncontrolled	Controlled	p-value	Uncontrolled	Controlled	p-value
N	**295**	**322**		**465**	**379**		**428**	**370**	
Age (years)	64.0 ± 8.4	59.5 ± 8.0	<0.001	67.4 ± 8.6	61.0 ± 7.7	<0.001	71.4 ± 8.2	64.4 ± 8.9	<0.001
Women (%)	118 (40.1)	145 (44.9)	0.233	186 (40.0)	223 (58.8)	<0.001	218 (50.9)	183 (49.5)	0.678
Swiss national (%)	210 (71.4)	220 (68.1)	0.371	339 (72.9)	261 (68.9)	0.198	312 (72.9)	251 (67.8)	0.118
Education (%)			0.917			0.512			0.049
High	31 (10.5)	35 (10.8)		62 (13.3)	61 (16.1)		52 (12.2)	68 (18.4)	
Middle	61 (20.8)	71 (22.0)		102 (21.9)	83 (21.9)		99 (23.1)	81 (21.9)	
Low	202 (68.7)	217 (67.2)		301 (64.7)	235 (62.0)		277 (64.7)	221 (59.7)	
Married/couple (%)	199 (67.7)	229 (70.9)	0.388	271 (58.3)	234 (61.7)	0.308	233 (54.4)	215 (58.1)	0.298
BMI (kg/m^2)	28.2 ± 4.4	27.8 ± 4.6	0.217	28.1 ± 4.6	27.1 ± 4.9	0.002	28.1 ± 4.6	27.0 ± 5.1	0.002
BMI categories (%)			0.250			0.001			0.001
Normal	65 (22.1)	90 (27.9)		111 (23.9)	132 (34.8)		112 (26.2)	142 (38.4)	
Overweight	136 (46.3)	141 (43.7)		216 (46.5)	162 (42.7)		185 (43.2)	142 (38.4)	
Obese	93 (31.6)	92 (28.5)		138 (29.7)	85 (22.4)		131 (30.6)	86 (23.2)	
Smoking (%)			0.714			0.239			0.151
Never	101 (34.4)	109 (33.8)		153 (32.9)	142 (37.5)		168 (39.3)	122 (33.0)	
Former	117 (39.8)	138 (42.7)		224 (48.2)	161 (42.5)		190 (44.4)	175 (47.3)	
Current	76 (25.9)	76 (23.5)		88 (18.9)	76 (20.1)		70 (16.4)	73 (19.7)	
Alcohol drinker (%)	224 (76.2)	232 (71.8)	0.218	352 (75.7)	280 (73.9)	0.544	285 (73.3)	240 (71.2)	0.538
Treatment for (%)									
Hypertension	158 (53.7)	145 (44.9)	0.028	266 (57.2)	170 (44.9)	<0.001	258 (60.3)	167 (45.1)	<0.001
Diabetes	68 (23.1)	27 (8.4)	<0.001	121 (26.0)	27 (7.1)	<0.001	102 (23.8)	55 (14.9)	0.001
Parental history (%)	63 (21.4)	101 (31.3)	0.006	95 (20.4)	123 (32.5)	<0.001	95 (22.2)	116 (31.4)	0.003
CVD risk (%)			<0.001			<0.001			<0.001

For peer review only - http://bmjopen.bmj.com/site/about/guidelines.xhtml

Other	95 (32.3)	173 (53.6)		140 (30.1)	225 (59.4)		162 (37.9)	193 (52.2)	
High	66 (22.5)	118 (36.5)		98 (21.1)	107 (28.2)		73 (17.1)	94 (25.4)	
Very high	133 (45.2)	32 (9.9)		227 (48.8)	47 (12.4)		193 (45.1)	83 (22.4)	
Number of drugs									
Including OTC	-	-		4 [3 - 6]	4 [2 - 5]	<0.001*	5 [3 - 7]	4 [3 - 6]	<0.001*
Excluding OTC	-	-		4 [2 - 6]	3 [2 - 5]	<0.001*	4 [3 - 7]	3 [2 - 6]	<0.001*
Genetic risk scores									
Total cholesterol	-2.8 ± 9.4	-3.9 ± 9.7	0.149	-3.5 ± 9.2	-3.6 ± 8.9	0.941	-4.2 ± 9.6	-3.2 ± 8.1	0.147
LDL-cholesterol	-2.2 ± 7.8	-3.2 ± 7.2	0.117	-2.5 ± 7.5	-2.3 ± 7.1	0.827	-3.2 ± 7.6	-2.5 ± 6.3	0.171
HDL-cholesterol	-6.5 ± 3.5	-6.8 ± 3.4	0.215	-6.6 ± 3.6	-6.9 ± 3.6	0.371	-6.5 ± 3.6	-6.9 ± 3.6	0.124
Hypolipidemic drug treatment (%)									
Statins	270 (91.5)	308 (95.7)	0.035	373 (80.4)	288 (75.8)	0.107	328 (76.6)	264 (71.4)	0.089
Fibrates	28 (9.5)	17 (5.3)	0.044	28 (6.0)	5 (1.3)	<0.001	18 (4.2)	14 (3.8)	0.762
Other	11 (3.7)	11 (3.4)	0.834	72 (15.5)	79 (20.8)	0.047	84 (19.6)	85 (23.0)	0.249

BMI, body mass index; CVD, cardiovascular disease; OTC, over the counter. Results are expressed as number of participants (column %) for categorical variables and as average ± standard deviation or as median [interquartile range] for continuous variables. Between-groups comparisons performed using chi-square for categorical variables and student's t-test or Kruskal-Wallis nonparametric test (*) for continuous variables.

For peer review only

For peer review only - http://bmjopen.bmj.com/site/about/guidelines.xhtml

Table 2: multivariable analysis of the factors associated with controlled dyslipidemia using ESC/EAS criteria, baseline (2003-2006), first (2009-2012) and second (2014-2017) follow-ups of the CoLaus|PsyCoLaus study, Lausanne, Switzerland.

	Baseline		First follow-up		Second follow-up	
	OR (95% CI)	p-value	OR (95% CI)	p-value	OR (95% CI)	p-value
Age (per 10 years increase)	0.44 (0.34 - 0.57)	<0.001	0.34 (0.27 - 0.43)	<0.001	0.35 (0.28 - 0.44)	<0.001
Man vs. woman	0.89 (0.58 - 1.36)	0.599	0.48 (0.34 - 0.70)	<0.001	1.23 (0.85 - 1.79)	0.273
Swiss vs. Non-Swiss	1.30 (0.85 - 1.97)	0.223	1.13 (0.79 - 1.64)	0.503	1.01 (0.69 - 1.48)	0.948
Education						
High	1 (ref.)		1 (ref.)		1 (ref.)	
Middle	1.11 (0.54 - 2.27)	0.782	0.83 (0.48 - 1.45)	0.521	0.61 (0.35 - 1.07)	0.087
Low	1.11 (0.59 - 2.11)	0.738	0.81 (0.49 - 1.33)	0.407	0.65 (0.40 - 1.07)	0.093
p-value for trend	0.738		0.407		0.093	
Married vs. not married	1.21 (0.80 - 1.82)	0.374	1.21 (0.86 - 1.71)	0.270	1.06 (0.74 - 1.5)	0.759
Body mass index categories						
Normal	1 (ref.)		1 (ref.)		1 (ref.)	
Overweight	0.59 (0.37 - 0.94)	0.028	0.85 (0.57 - 1.26)	0.413	0.61 (0.40 - 0.92)	0.019
Obese	0.92 (0.54 - 1.56)	0.753	0.96 (0.59 - 1.55)	0.852	0.64 (0.40 - 1.04)	0.074
p-value for trend	0.753		0.852		0.074	
Smoking categories						
Never	1 (ref.)		1 (ref.)		1 (ref.)	
Former	1.54 (0.98 - 2.40)	0.059	1.10 (0.75 - 1.62)	0.618	1.38 (0.94 - 2.02)	0.099
Current	0.63 (0.36 - 1.08)	0.094	0.85 (0.52 - 1.39)	0.512	1.04 (0.61 - 1.75)	0.892
p-value for trend	0.094		0.512		0.892	
Alcohol drinker (yes vs. no)	0.66 (0.42 - 1.03)	0.068	0.91 (0.62 - 1.35)	0.645	0.64 (0.43 - 0.94)	0.024
Antihypertensive treatment (yes vs. no)	1.05 (0.70 - 1.57)	0.820	1.18 (0.82 - 1.69)	0.368	0.77 (0.53 - 1.12)	0.176
Parental history (yes vs. no)	1.00 (0.64 - 1.56)	0.998	1.08 (0.74 - 1.59)	0.690	0.79 (0.53 - 1.18)	0.256
CVD risk						
Other	1 (ref.)		1 (ref.)		1 (ref.)	

For peer review only

High	1.25 (0.76 - 2.04)	0.379	0.68 (0.45 - 1.04)	0.074	1.42 (0.88 - 2.28)	0.153
Very high	0.11 (0.06 - 0.18)	<0.001	0.12 (0.08 - 0.19)	<0.001	0.38 (0.25 - 0.59)	<0.001
p-value for trend	<0.001		<0.001		<0.001	
LDL genetic risk score quartiles						
First	1 (ref.)		1 (ref.)		1 (ref.)	
Second	1.11 (0.65 - 1.89)	0.696	0.97 (0.61 - 1.54)	0.883	1.31 (0.81 - 2.12)	0.264
Third	0.70 (0.41 - 1.19)	0.185	0.96 (0.60 - 1.53)	0.866	1.57 (0.97 - 2.51)	0.064
Fourth	0.74 (0.43 - 1.25)	0.259	1.07 (0.67 - 1.72)	0.764	1.44 (0.89 - 2.32)	0.133
p-value for trend	0.107		0.781		0.099	
Hypolipidemic drug treatment						
Statins	1.56 (0.18 - 13.8)	0.690	1.00 (0.55 - 1.81)	0.998	1.42 (0.81 - 2.51)	0.223
Fibrates	0.67 (0.08 - 5.35)	0.707	0.13 (0.04 - 0.43)	0.001	1.20 (0.46 - 3.15)	0.714
Other	0.94 (0.34 - 2.61)	0.910	0.57 (0.31 - 1.04)	0.067	0.71 (0.40 - 1.26)	0.237

OR, odds ratio; CI, confidence interval; CVD, cardiovascular disease. Results are expressed as odds ratio and (95% CI). Statistical analysis using logistic regression.

For peer review only

The distribution of the statin generation or potency according to dyslipidemia control is provided in **supplemental table 7**. Controlled participants had a higher prevalence of third generation (first follow up) or high potency statins than inadequately controlled participants. When posology was used to estimate potency, no differences were found. The results of the multivariable analyses taking into account statin generation or statin potency irrespective of the posology are provided in **tables 3 and 4**, respectively. In both analyses, increasing age or CVD risk led to a lower likelihood of being controlled, while increasing statin generation or potency led to a higher likelihood of being controlled. When posology was used to estimate potency, the association was no longer significant (**table 5**).

For peer review only

For peer review only - http://bmjopen.bmj.com/site/about/guidelines.xhtml

Table 3: multivariable analysis of the factors associated with controlled dyslipidemia using ESC/EAS criteria, first (2009-2012) and second (2014-2017) follow-ups of the CoLaus|PsyCoLaus study, Lausanne, Switzerland.

	**First follow-up**		**Second follow-up**	
	**OR (95% CI)**	**p-value**	**OR (95% CI)**	**p-value**
Age (per 10 years increase)	0.31 (0.23 - 0.41)	<0.001	0.35 (0.27 - 0.47)	<0.001
Man vs. woman	0.60 (0.39 - 0.92)	0.018	1.40 (0.90 - 2.16)	0.134
Swiss vs. Non-Swiss	1.17 (0.76 - 1.80)	0.474	1.16 (0.75 - 1.80)	0.502
Education				
High	1 (ref.)		1 (ref.)	
Middle	0.78 (0.40 - 1.53)	0.476	1.08 (0.54 - 2.15)	0.821
Low	0.88 (0.49 - 1.59)	0.681	1.04 (0.57 - 1.90)	0.895
p-value for trend	0.681		0.895	
Married vs. not married	1.23 (0.82 - 1.83)	0.317	1.19 (0.79 - 1.78)	0.407
Body mass index categories				
Normal	1 (ref.)		1 (ref.)	
Overweight	0.84 (0.52 - 1.35)	0.474	0.56 (0.35 - 0.92)	0.023
Obese	0.91 (0.53 - 1.58)	0.749	0.54 (0.31 - 0.95)	0.032
p-value for trend	0.749		0.032	
Smoking categories				
Never	1 (ref.)		1 (ref.)	
Former	1.09 (0.70 - 1.71)	0.695	1.21 (0.78 - 1.88)	0.384
Current	0.84 (0.46 - 1.51)	0.560	0.93 (0.51 - 1.71)	0.820
p-value for trend	0.560		0.820	
Alcohol drinker (yes vs. no)	0.79 (0.50 - 1.25)	0.316	0.72 (0.46 - 1.12)	0.146
AntiHTA ttt (yes vs. no)	0.97 (0.63 - 1.51)	0.903	0.80 (0.52 - 1.25)	0.337
Parental history (yes vs. no)	1.27 (0.80 - 2.02)	0.310	0.76 (0.48 - 1.23)	0.267
CVD risk				
Other	1 (ref.)		1 (ref.)	
High	0.63 (0.39 - 1.02)	0.061	1.32 (0.76 - 2.31)	0.327
Very high	0.08 (0.05 - 0.14)	<0.001	0.35 (0.21 - 0.58)	<0.001
p-value for trend	<0.001		<0.001	
LDL genetic risk score quartiles				
First	1 (ref.)		1 (ref.)	
Second	0.90 (0.53 - 1.54)	0.707	1.67 (0.95 - 2.93)	0.076
Third	0.89 (0.52 - 1.52)	0.665	1.79 (1.04 - 3.07)	0.036
Fourth	1.11 (0.65 - 1.92)	0.696	1.64 (0.93 - 2.86)	0.085
p-value for trend	0.725		0.085	
Number of drugs (per 1 unit)	1.15 (1.05 - 1.25)	0.002	1.07 (0.99 - 1.15)	0.069
Statin generation				
First	1 (ref.)		1 (ref.)	
Second	1.90 (1.18 - 3.05)	0.008	1.90 (1.08 - 3.36)	0.026
Third	3.62 (1.65 - 7.92)	0.001	2.18 (1.05 - 4.51)	0.036
p-value for trend	0.001		0.036	

For peer review only - http://bmjopen.bmj.com/site/about/guidelines.xhtml

Fibrates	NC		2.55 (0.19 - 34.1)	0.480
Other hypolipidemic drugs	0.90 (0.38 - 2.11)	0.800	1.13 (0.51 - 2.51)	0.762

OR, odds ratio; CI, confidence interval; antiHTA ttt, antihypertensive drug treatment; NC, not computable. Results are expressed as odds ratio and (95% confidence interval). Statistical analysis using logistic regression.

For peer review only

For peer review only - http://bmjopen.bmj.com/site/about/guidelines.xhtml

Table 4: multivariable analysis of the factors associated with controlled dyslipidemia using ESC/EAS criteria, first (2009-2012) and second (2014-2017) follow-ups of the CoLaus|PsyCoLaus study, Lausanne, Switzerland.

	First follow-up		Second follow-up	
	**OR (95% CI)**	**p-value**	**OR (95% CI)**	**p-value**
Age (per 10 years increase)	0.31 (0.23 - 0.41)	<0.001	0.36 (0.27 - 0.47)	<0.001
Man vs. woman	0.59 (0.38 - 0.90)	0.014	1.36 (0.88 - 2.11)	0.171
Swiss vs. Non-Swiss	1.16 (0.76 - 1.78)	0.495	1.17 (0.75 - 1.81)	0.487
Education				
High	1 (ref.)		1 (ref.)	
Middle	0.76 (0.39 - 1.48)	0.415	1.04 (0.52 - 2.08)	0.908
Low	0.87 (0.48 - 1.57)	0.649	1.02 (0.56 - 1.87)	0.937
p-value for trend	0.649		0.937	
Married vs. not married	1.23 (0.83 - 1.84)	0.299	1.20 (0.80 - 1.79)	0.386
Body mass index categories				
Normal	1 (ref.)		1 (ref.)	
Overweight	0.86 (0.53 - 1.38)	0.523	0.57 (0.35 - 0.93)	0.025
Obese	0.93 (0.54 - 1.61)	0.807	0.53 (0.30 - 0.94)	0.031
p-value for trend	0.807		0.031	
Smoking categories				
Never	1 (ref.)		1 (ref.)	
Former	1.13 (0.72 - 1.77)	0.590	1.19 (0.77 - 1.85)	0.428
Current	0.86 (0.48 - 1.55)	0.614	0.90 (0.49 - 1.65)	0.729
p-value for trend	0.614		0.729	
Alcohol drinker (yes vs. no)	0.81 (0.51 - 1.27)	0.360	0.68 (0.43 - 1.08)	0.101
AntiHTA ttt (yes vs. no)	0.95 (0.62 - 1.47)	0.821	0.80 (0.51 - 1.25)	0.327
Parental history (yes vs. no)	1.25 (0.79 - 1.99)	0.338	0.75 (0.47 - 1.21)	0.240
CVD risk				
Other	1 (ref.)		1 (ref.)	
High	0.63 (0.39 - 1.02)	0.060	1.37 (0.78 - 2.40)	0.275
Very high	0.08 (0.05 - 0.14)	<0.001	0.35 (0.21 - 0.58)	<0.001
p-value for trend	<0.001		<0.001	
LDL genetic risk score quartiles				
First	1 (ref.)		1 (ref.)	
Second	0.89 (0.52 - 1.53)	0.680	1.63 (0.92 - 2.86)	0.092
Third	0.92 (0.54 - 1.57)	0.757	1.76 (1.02 - 3.03)	0.041
Fourth	1.11 (0.65 - 1.91)	0.702	1.58 (0.90 - 2.77)	0.112
p-value for trend	0.694		0.109	
Number of drugs (per 1 unit)	1.14 (1.05 - 1.25)	0.002	1.06 (0.99 - 1.15)	0.099
Statin potency, no posology				
Low	1 (ref.)		1 (ref.)	
Intermediate	1.98 (1.18 - 3.34)	0.010	1.50 (0.80 - 2.82)	0.209
High	2.08 (1.25 - 3.46)	0.005	2.25 (1.26 - 4.01)	0.006
p-value for trend	0.005		0.006	

For peer review only

For peer review only - http://bmjopen.bmj.com/site/about/guidelines.xhtml

Fibrates	NC		2.16 (0.16 - 29.1)	0.560
Other hypolipidemic drugs	0.95 (0.41 - 2.22)	0.905	1.07 (0.48 - 2.37)	0.867

OR, odds ratio; CI, confidence interval; antiHTA ttt, antihypertensive drug treatment; NC, not computable. Results are expressed as odds ratio and (95% confidence interval). Statistical analysis using logistic regression.

For peer review only

Table 5: multivariable analysis of the factors associated with controlled dyslipidemia using ESC/EAS criteria, first (2009-2012) and second (2014-2017) follow-ups of the CoLaus|PsyCoLaus study, Lausanne, Switzerland.

	First follow-up		Second follow-up	
	**OR (95% CI)**	**p-value**	**OR (95% CI)**	**p-value**
Age (per 10 years increase)	0.31 (0.24 - 0.42)	<0.001	0.37 (0.27 - 0.50)	<0.001
Man vs. woman	0.56 (0.36 - 0.86)	0.008	1.45 (0.91 - 2.31)	0.120
Swiss vs. Non-Swiss	1.14 (0.74 - 1.78)	0.548	1.11 (0.69 - 1.76)	0.672
Education				
High	1 (ref.)		1 (ref.)	
Middle	0.74 (0.37 - 1.48)	0.396	0.95 (0.46 - 1.95)	0.882
Low	0.86 (0.47 - 1.56)	0.619	0.94 (0.50 - 1.76)	0.835
p-value for trend	0.619		0.835	
Married vs. not married	1.25 (0.83 - 1.88)	0.29	1.17 (0.76 - 1.80)	0.481
Body mass index categories				
Normal	1 (ref.)		1 (ref.)	
Overweight	0.87 (0.54 - 1.42)	0.586	0.62 (0.37 - 1.05)	0.073
Obese	0.89 (0.50 - 1.56)	0.677	0.56 (0.31 - 1.02)	0.058
p-value for trend	0.677		0.058	
Smoking categories				
Never	1 (ref.)		1 (ref.)	
Former	1.12 (0.71 - 1.77)	0.629	1.12 (0.7 - 1.78)	0.636
Current	0.91 (0.50 - 1.65)	0.747	0.82 (0.42 - 1.58)	0.545
p-value for trend	0.747		0.545	
Alcohol drinker (yes vs. no)	0.73 (0.45 - 1.16)	0.183	0.58 (0.35 - 0.95)	0.031
AntiHTA ttt (yes vs. no)	0.99 (0.64 - 1.55)	0.977	0.89 (0.55 - 1.42)	0.618
Parental history (yes vs. no)	1.24 (0.77 - 1.98)	0.382	0.85 (0.51 - 1.41)	0.529
CVD risk				
Other	1 (ref.)		1 (ref.)	
High	0.64 (0.39 - 1.04)	0.074	1.29 (0.71 - 2.33)	0.400
Very high	0.08 (0.05 - 0.15)	<0.001	0.35 (0.20 - 0.61)	<0.001
p-value for trend	<0.001		<0.001	
LDL genetic risk score quartiles				
First	1 (ref.)		1 (ref.)	
Second	1.01 (0.58 - 1.75)	0.979	1.63 (0.89 - 2.96)	0.111
Third	1.15 (0.66 - 1.99)	0.623	1.89 (1.06 - 3.36)	0.031
Fourth	1.24 (0.71 - 2.16)	0.452	1.68 (0.93 - 3.02)	0.085
p-value for trend	0.392		0.073	
Number of drugs (per 1 unit)	1.13 (1.03 - 1.23)	0.008	1.04 (0.96 - 1.13)	0.366
Statin potency, with posology				
Low	1 (ref.)		1 (ref.)	
Intermediate	1.10 (0.73 - 1.65)	0.656	1.37 (0.86 - 2.17)	0.183
High	1.35 (0.63 - 2.88)	0.437	1.69 (0.89 - 3.21)	0.110
p-value for trend	0.437		0.110	

For peer review only

Fibrates	NC		2.20 (0.16 - 31.2)	0.559
Other hypolipidemic drugs	0.91 (0.38 - 2.21)	0.839	1.06 (0.45 - 2.47)	0.898

OR, odds ratio; CI, confidence interval; antiHTA ttt, antihypertensive drug treatment; NC, not computable. Results are expressed as odds ratio and (95% confidence interval). Statistical analysis using logistic regression.

Prevalence and factors associated with control of dyslipidemia, GSLA criteria

Prevalence of adequately managed dyslipidemia was 70%, 68% and 83% in the baseline, first and second follow-ups, respectively. The results of the analysis using the GSLA criteria stratified by survey are summarized in **supplemental tables 8 to 13**.

On bivariate analysis, controlled participants had lower CVD risk (all surveys), lower BMI (first and second follow-ups) and lower prevalence of smoking (first follow-up) than inadequately controlled participants; no differences were found regarding GRS (**supplemental table 8**). On multivariable analysis, increased CVD risk was negatively associated with dyslipidemia control in all surveys; men had a higher likelihood of being controlled (baseline and second follow-up) and alcohol consumption decreased likelihood of control (baseline); no association was found with LDL GRS or the class of hypolipidemic drug (**supplemental table 9**).

The distribution of the statin generation or potency according to dyslipidemia control is provided in **supplemental table 10**. Controlled participants had a higher prevalence of third generation or high potency statins than inadequately controlled participants. When posology was used to estimate potency, no differences were found. The results of the multivariable analyses taking into account statin generation or statin potency irrespective of the posology are provided in **supplemental tables 11 and 12**, respectively. In both analyses, increasing CVD risk led to a lower likelihood of being adequately controlled, while being male, increasing number of drugs or increasing statin generation or potency led a higher likelihood of being adequately controlled. When posology was used to estimate statin potency, the association was no longer significant (**supplemental table 13**).

For peer review only - http://bmjopen.bmj.com/site/about/guidelines.xhtml

Specific SNPs

The p-values for the associations between 51 specific SNPs and dyslipidemia control are presented in **supplemental table 14**. Most statistically significant associations were found for *SLCO1B1* (Solute Carrier Organic Anion Transporter Family Member 1B1), but no consistent association was found overall.

Discussion

Our results show that individuals at high risk of CVD present an increased risk of mismanagement of dyslipidemia, which was consistent among the three survey periods. The use of more potent statins increased the likelihood of dyslipidemia control, while lipid GRS were not associated with dyslipidemia control.

Prevalence of controlled dyslipidemia

Prevalence of controlled dyslipidemia varied between 45% and 52% according to ESC/EAS criteria and between 68% and 83% according to GSLA criteria. Those values are higher than the values reported by the EUROASPIRE IV, where a third of participants (32.7%) achieved the target level of <2.5 mmol/L for LDL cholesterol [16]. Still, comparisons are difficult as EUROASPIRE IV focused on high-risk participants only. Importantly, prevalence rates of adequately managed dyslipidemia were much higher using GSLA than ESC/EAS criteria. Hence, a clinician using GSLA criteria will lower LDL levels to a lesser level than using ESC/EAS criteria. Given that CVD risk decreases linearly with LDL cholesterol levels (all other factors being equal) [17], the decrease in CVD risk is expected to be lower using GSLA than using ESC/EAS criteria. It would be important to evaluate if people managed according to the GSLA criteria achieve the same level of protection against CVD as if they were managed according to the ESC/EAS criteria.

Factors associated with controlled dyslipidemia

Participants at high risk of CVD had a higher likelihood of being inadequately managed in all surveys, irrespective of the criteria considered. Those findings are consistent with a recent review of

For peer review only - http://bmjopen.bmj.com/site/about/guidelines.xhtml

European studies [18] and a large cross-sectional European study [19] where control rates were lower than 20%. Possible explanations include the fact that subjects at high CVD risk should have much lower lipid values, thus more difficult to achieve.

Similar to a Polish study [11] but contrary to a US study [20], the prevalence of highly potent statins increased from 41% in 2009-2012 to 56% in 2014-2017. This value is higher than reported in the EUROASPIRE V [4] study, where 49.9% of participants were on high intensity therapy. Importantly, participants on high potency statins achieved better control, a finding also reported in the EUROASPIRE V [4] and the DA VINCI [19] studies. The 2019 ESC/EAS guidelines for management of dyslipidemia recommend that high potency statins at highest recommended tolerable dose be initially applied to control lipid levels [3]. Our results thus strengthen the importance of such recommendation, and GPs should be urged to shift to more potent statins to achieve better results. Still, our results also suggest that, despite a higher prescription rate of highly potent statins, those drugs are not prescribed at their full potency/posology, and that a sizable fraction of treated subjects fails to reach lipid targets.

Other clinical and socio-demographic factors were associated with dyslipidemia control, but associations were inconsistent between study periods or between ESC/EAS and GSLA criteria. Increasing age was negatively associated with dyslipidemia control using ESC/EAS criteria but no association was found using GSLA criteria. Either no association [21], an inverse association [22], or a positive association [23] between age and dyslipidemia control have been reported. Similarly, men achieved better control than women using the GSLA criteria, while no consistent association was found using the ESC/EAS criteria. Better control rates have also been reported in the USA [24], while the inverse association was reported in Germany [22]. Such discrepancies might be related to the criteria applied, as age and gender might be stronger or weaker determinants of CVD risk in some risk equations compared to others. No association was found between nationality, education, marital status, job type or BMI categories and dyslipidemia control. Our findings replicate those of other studies where no association between education [21], marital status [21, 22] and dyslipidemia control

For peer review only

was found. Overall, our results suggest that the socio-demographic factors associated with dyslipidemia control differ according to country and to the criteria used to estimate CVD risk.

Genetic scores and individual SNPs

Several authors suggested that genetic profiling could be used to guide statin treatment and thus improve outcomes [7, 8]. A meta-analysis published in 2015 concluded that people with the highest burden of genetic risk derived the largest relative and absolute clinical benefit from statin therapy [25], although such statement could also apply to people for whom cardiovascular risk was assessed using clinical data. Further, the initial promises regarding genetic testing of the kinesin-like protein 6 (KIF6) gene to guide statin prescription (the StatinCheck® test) were not confirmed [26]. In this study, no association between genetic scores for lipid markers and statin efficiency was found. Possible reasons include the small effect of each individual SNP [27], as a set of 95 SNPs explained less than 15% of total lipid variance [28], or the progressive blunting of the genetic effect by advanced aging as found for blood pressure [29]. Thus, our results suggest that genetic profiling of subjects prior to initiation of statin therapy might clinically irrelevant, and such profiling is not stated in the current ESC/EAS guidelines for the management of dyslipidemias [3]. Nevertheless, several associations were found with the *SLCO1B1* gene. Some authors have suggested that genetic variations in this gene are associated with response to statins [8]. Hence, this gene might be of interest to adapt statin treatment, and it would be important that other studies be conducted to confirm our findings.

Study limitations

This study has several limitations worth acknowledging. Firstly, the sample size was relatively small and our study was likely underpowered to detect the minute associations between the genetic scores and dyslipidemia control. Still, should those GRS be applied in clinical practice, their effect should be large enough to allow choosing between several statins in a given individual. Secondly, the analysis was restricted to Switzerland, and findings might be generalizable to other countries or ethnicities. Still, most findings agree with larger studies such as EUROASPIRE V [4] or DA VINCI [19].

For peer review only - http://bmjopen.bmj.com/site/about/guidelines.xhtml

For peer review only

Conclusion

Management of dyslipidemia is suboptimal in Switzerland, especially for individuals at high cardiovascular risk. The effectiveness of high potency statins is hampered by low posology. GRS are not associated with dyslipidemia control, but the effect of *SLCO1B1* in statin therapy should be further investigated.

For peer review only

Funding

The CoLaus|PsyCoLaus study was and is supported by research grants from GlaxoSmithKline (N/A), the Faculty of Biology and Medicine of Lausanne (N/A), and the Swiss National Science Foundation (grants 33CSCO-122661, 33CS30-139468, 33CS30-148401 and 33CS30_177535/1). Valeriya Chekanova received an excellence scholarship of the Swiss government (N/A) to conduct research in Switzerland for one year.

Data availability

Non-identifiable individual-level data are available for researchers who seek to answer questions related to health and disease in the context of research projects who meet the criteria for data sharing by research committees. Please follow the instructions at https://www.colaus-psycolaus.ch/ for information on how to submit an application for gaining access to CoLaus|PsyCoLaus data.

Conflict of interest

The authors report no conflict of interest.

Credit author statement

Valeriya Chekanova: Investigation, Methodology, Writing – Original draft preparation, Visualization. **Pedro Marques-Vidal**: Conceptualization, Methodology, Data curation, Formal analysis, Writing – Reviewing and editing, Visualization. **Nazanin Abolhassani**: Writing – Reviewing and editing. **Julien Vaucher**: Reviewing and editing.

The authors had full access to the data and took responsibility for its integrity. All authors have read and agreed to the written manuscript. PMV had full access to the data and is the guarantor of the study.

For peer review only

Ethical statement

The institutional Ethics Committee of the University of Lausanne, which afterwards became the Ethics Commission of Canton Vaud (www.cer-vd.ch) approved the baseline (reference 16/03), the first (reference 33/09) and the second (reference 26/14) follow-ups. The approval was confirmed in 2021 (reference PB_2018-00038, 239/09). The study was performed in agreement with the Helsinki declaration and its former amendments, and in accordance with the applicable Swiss legislation. All participants gave their signed informed consent before entering the study.

For peer review only

For peer review only - http://bmjopen.bmj.com/site/about/guidelines.xhtml

References

1 Cholesterol Treatment Trialists C, Fulcher J, O'Connell R, et al. Efficacy and safety of LDL-lowering therapy among men and women: meta-analysis of individual data from 174,000 participants in 27 randomised trials. *Lancet* 2015;**385**:1397-405.

2 Singh BM, Lamichhane HK, Srivatsa SS, et al. Role of Statins in the Primary Prevention of Atherosclerotic Cardiovascular Disease and Mortality in the Population with Mean Cholesterol in the Near-Optimal to Borderline High Range: A Systematic Review and Meta-Analysis. *Adv Prev Med* 2020;**2020**:6617905.

3 Mach F, Baigent C, Catapano AL, et al. 2019 ESC/EAS Guidelines for the management of dyslipidaemias: lipid modification to reduce cardiovascular risk. *Eur Heart J* 2020;**41**:111-88.

4 De Backer G, Jankowski P, Kotseva K, et al. Management of dyslipidaemia in patients with coronary heart disease: Results from the ESC-EORP EUROASPIRE V survey in 27 countries. *Atherosclerosis* 2019;**285**:135-46.

5 Mosca L, Linfante AH, Benjamin EJ, et al. National study of physician awareness and adherence to cardiovascular disease prevention guidelines. *Circulation* 2005;**111**:499-510.

6 Naderi SH, Bestwick JP, Wald DS. Adherence to drugs that prevent cardiovascular disease: meta-analysis on 376,162 patients. *Am J Med* 2012;**125**:882-7 e1.

7 Cano-Corres R, Candas-Estebanez B, Padro-Miquel A, et al. Influence of 6 genetic variants on the efficacy of statins in patients with dyslipidemia. *J Clin Lab Anal* 2018;**32**:e22566.

8 Maxwell WD, Ramsey LB, Johnson SG, et al. Impact of pharmacogenetics on efficacy and safety of statin therapy for dyslipidemia. *Pharmacotherapy* 2017;**37**:1172-90.

9 Firmann M, Mayor V, Vidal PM, et al. The CoLaus study: a population-based study to investigate the epidemiology and genetic determinants of cardiovascular risk factors and metabolic syndrome. *BMC Cardiovasc Disord* 2008;**8**:6.

10 Stone NJ, Robinson JG, Lichtenstein AH, et al. 2013 ACC/AHA guideline on the treatment of blood cholesterol to reduce atherosclerotic cardiovascular risk in adults: a report of the American College of Cardiology/American Heart Association Task Force on Practice Guidelines. *J Am Coll Cardiol* 2014;**63**:2889-934.

11 Pajak A, Szafraniec K, Polak M, et al. Changes in the prevalence, treatment, and control of hypercholesterolemia and other dyslipidemias over 10 years in Poland: the WOBASZ study. *Pol Arch Med Wewn* 2016;**126**:642-52.

12 Marques-Vidal P, Rodondi N, Bochud M, et al. Predictive accuracy and usefulness of calibration of the ESC SCORE in Switzerland. *Eur J Cardiovasc Prev Rehabil* 2008;**15**:402-8.

For peer review only - http://bmjopen.bmj.com/site/about/guidelines.xhtml

13 Moser M, Gencer B, Rodondi N. [Recommendations for management of dyslipidemia in 2014]. *Rev Med Suisse* 2014;**10**:518, 20-4.

14 Delaneau O, Zagury JF, Marchini J. Improved whole-chromosome phasing for disease and population genetic studies. *Nat Methods* 2013;**10**:5-6.

15 Spracklen CN, Saftlas AF, Triche EW, et al. Genetic Predisposition to Dyslipidemia and Risk of Preeclampsia. *Am J Hypertens* 2015;**28**:915-23.

16 Kotseva K, De Bacquer D, De Backer G, et al. Lifestyle and risk factor management in people at high risk of cardiovascular disease. A report from the European Society of Cardiology European Action on Secondary and Primary Prevention by Intervention to Reduce Events (EUROASPIRE) IV cross-sectional survey in 14 European regions. *Eur J Prev Cardiol* 2016;**23**:2007-18.

17 Cholesterol Treatment Trialists C, Baigent C, Blackwell L, et al. Efficacy and safety of more intensive lowering of LDL cholesterol: a meta-analysis of data from 170,000 participants in 26 randomised trials. *Lancet* 2010;**376**:1670-81.

18 Bruckert E, Parhofer KG, Gonzalez-Juanatey JR, et al. Proportion of High-Risk/Very High-Risk Patients in Europe with Low-Density Lipoprotein Cholesterol at Target According to European Guidelines: A Systematic Review. *Adv Ther* 2020;**37**:1724-36.

19 Ray KK, Molemans B, Schoonen WM, et al. EU-Wide Cross-Sectional Observational Study of Lipid-Modifying Therapy Use in Secondary and Primary Care: the DA VINCI study. *Eur J Prev Cardiol* 2021;**28**:1279-89.

20 Gu A, Kamat S, Argulian E. Trends and disparities in statin use and low-density lipoprotein cholesterol levels among US patients with diabetes, 1999-2014. *Diabetes Res Clin Pract* 2018;**139**:1-10.

21 Sozmen K, Unal B, Sakarya S, et al. Determinants of prevalence, awareness, treatment and control of high LDL-C in Turkey. *Anatol J Cardiol* 2016;**16**:370-84.

22 Tiffe T, Wagner M, Rucker V, et al. Control of cardiovascular risk factors and its determinants in the general population- findings from the STAAB cohort study. *BMC Cardiovasc Disord* 2017;**17**:276.

23 Boo S, Yoon YJ, Oh H. Evaluating the prevalence, awareness, and control of hypertension, diabetes, and dyslipidemia in Korea using the NHIS-NSC database: A cross-sectional analysis. *Medicine (Baltimore)* 2018;**97**:e13713.

24 Peters SAE, Muntner P, Woodward M. Sex Differences in the Prevalence of, and Trends in, Cardiovascular Risk Factors, Treatment, and Control in the United States, 2001 to 2016. *Circulation* 2019;**139**:1025-35.

For peer review only - http://bmjopen.bmj.com/site/about/guidelines.xhtml

25 Mega JL, Stitziel NO, Smith JG, et al. Genetic risk, coronary heart disease events, and the clinical benefit of statin therapy: an analysis of primary and secondary prevention trials. *Lancet* 2015;**385**:2264-71.

26 Ridker PM, MacFadyen JG, Glynn RJ, et al. Kinesin-like protein 6 (KIF6) polymorphism and the efficacy of rosuvastatin in primary prevention. *Circ Cardiovasc Genet* 2011;**4**:312-7.

27 Waterworth DM, Ricketts SL, Song K, et al. Genetic variants influencing circulating lipid levels and risk of coronary artery disease. *Arterioscler Thromb Vasc Biol* 2010;**30**:2264-76.

28 Teslovich TM, Musunuru K, Smith AV, et al. Biological, clinical and population relevance of 95 loci for blood lipids. *Nature* 2010;**466**:707-13.

29 Simino J, Shi G, Bis JC, et al. Gene-age interactions in blood pressure regulation: a large-scale investigation with the CHARGE, Global BPgen, and ICBP Consortia. *Am J Hum Genet* 2014;**95**:24-38.

For peer review only

For peer review only - http://bmjopen.bmj.com/site/about/guidelines.xhtml

Supplemental figure 1: flowchart of the selection of participants, baseline (2003-2006), first (2009-2012) and second (2014-2017) follow-ups of the CoLaus|PsyCoLaus study, Lausanne, Switzerland

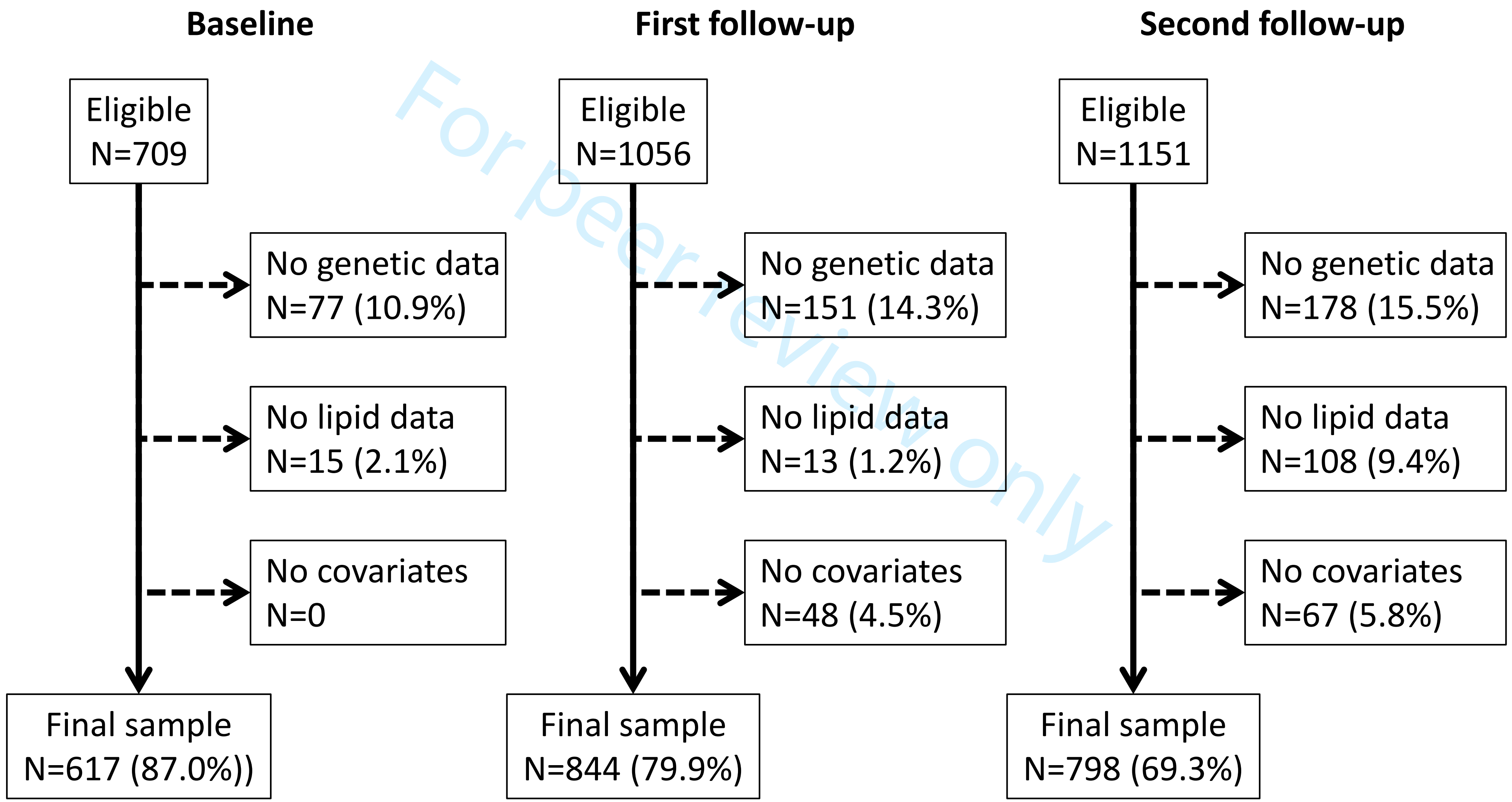

For peer review only

Supplemental figure 2: distribution of hypolipidemic drug classes, baseline (2003-2006), first (2009-2012) and second (2014-2017) follow-ups of the CoLaus|PsyCoLaus study, Lausanne, Switzerland

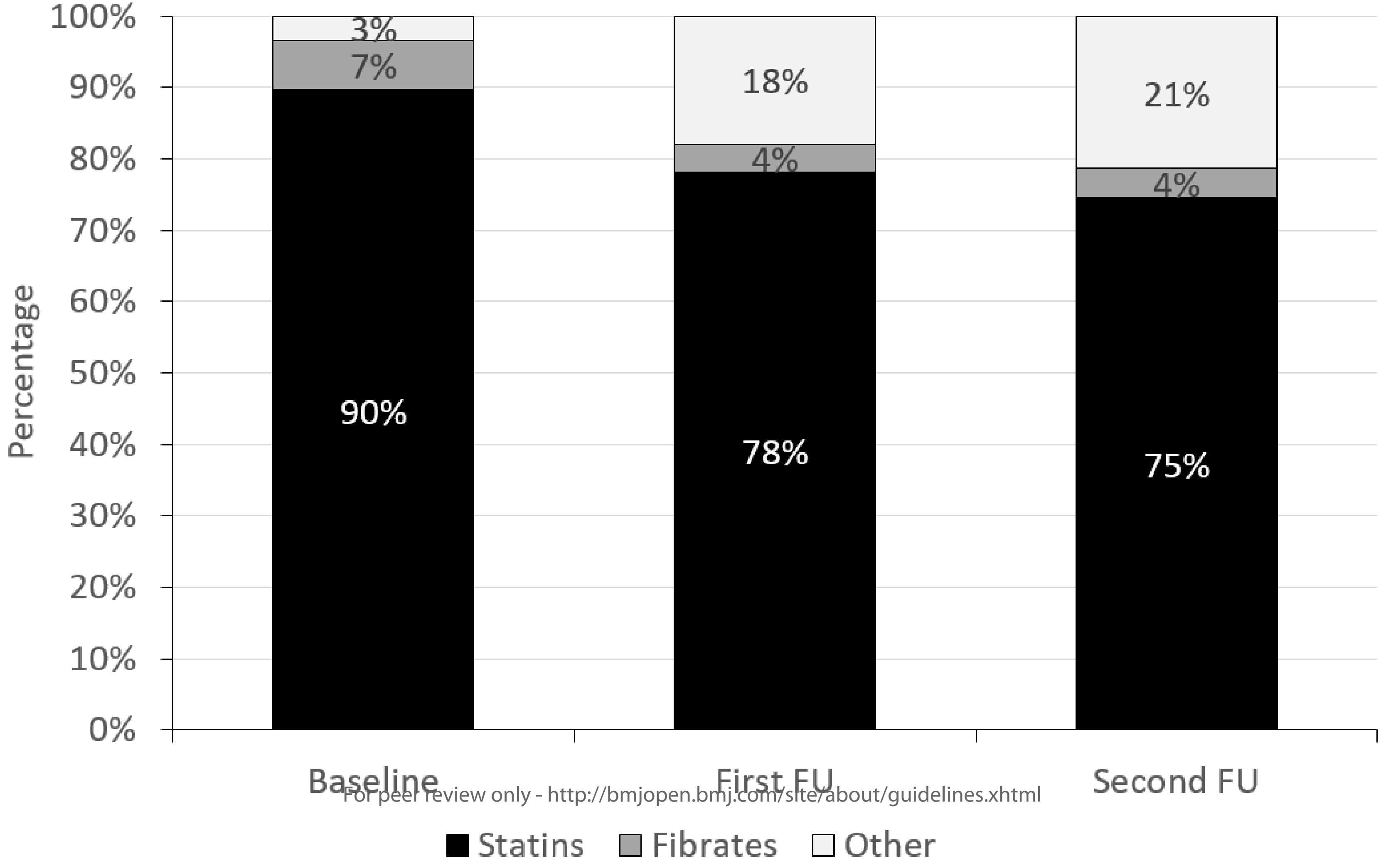

Supplemental figure 3: Generation and potency of statin drugs, first (2009-2012) and second (2014-2017) follow-ups of the CoLaus|PsyCoLaus study, Lausanne, Switzerland. Black: first generation or low potency; dark gray, second generation or intermediate potency; light gray, third generation or high potency.

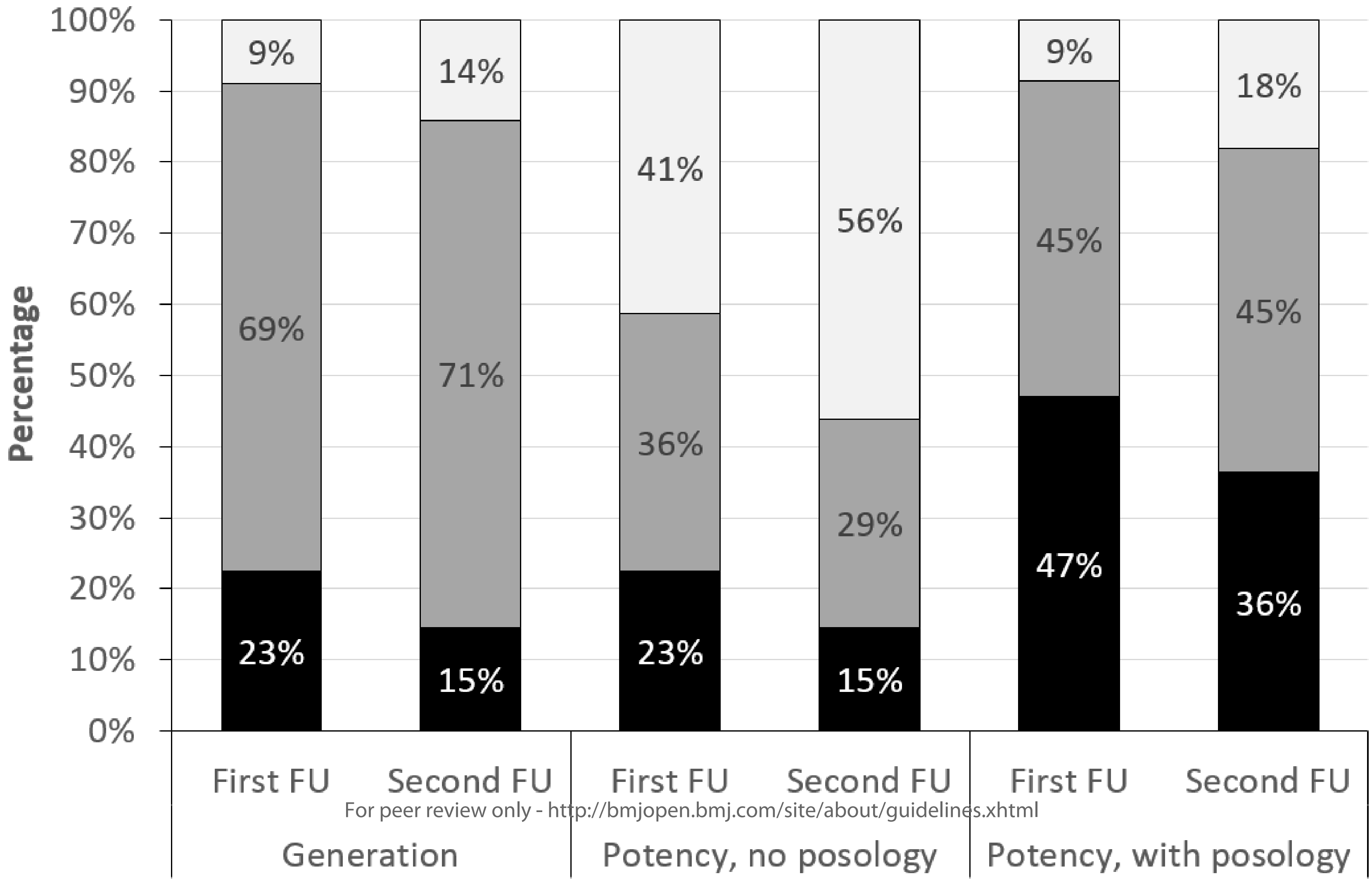

Supplemental figure 4: comparison of statin potency considering or not posology, first (2009-2012) and second (2014-2017) follow-ups of the CoLaus|PsyCoLaus study, Lausanne, Switzerland.

First follow-up

		With posology		
		Low	Intermediate	High
Without posology	Low	85	87	0
	Intermediate	283	7	0
	High	0	256	70

Second follow-up

		With posology		
		Low	Intermediate	High
Without posology	Low	67	43	0
	Intermediate	197	8	0
	High	0	282	136

Supplemental information

Supplemental table 1: statins classification regarding generation and potency.

Statin	ATC code	Generation	Potency, no posology	Potency, with posology
lovastatin	C10AA02	First	Low	20 low<br>40 intermediate
pravastatin	C10AA03	First	Low	<40 low<br>40-80 intermediate
fluvastatin	C10AA04	First	Low	10 low<br>40-80 intermediate
simvastatin	C10AA01	Second	Intermediate	20-40 intermediate<br>80 high
pitavastatin	C10AA08	Second	Intermediate	2 intermediate<br>4 high
atorvastatin	C10AA05	Second	High	10-20 intermediate<br>40-80 high
cerivastatin	C10AA06	Third	High	100 intermediate<br>300 high
rosuvastatin	C10AA07	Third	High	5-10 intermediate<br>20 high

Statins were ordered according to their potency. Adapted from [1, 2].

For peer review only

For peer review only - http://bmjopen.bmj.com/site/about/guidelines.xhtml

Supplemental table 2: LDL-cholesterol thresholds used to define control of dyslipidemia according to the European Society of Cardiology / European Atherosclerosis Society (ESC/EAS) criteria.

Risk categories according to SCORE	Control
Very high risk	
10–y risk >10%, diabetes or personal history of CVD	<1.8 mmol/L (70 mg/dL)
High risk	
10–y risk 5% – 10%	<2.6 mmol/L (100 mg/dL)
Other categories	
Less than 2 CVD risk factors and 10–y risk <5%	<4.0 mmol/L (155 mg/dL)

ESC/EAS criteria as stated in [3]. Risk categories based on the SCORE equation recalibrated for the Swiss population [4].

For peer review only

For peer review only - http://bmjopen.bmj.com/site/about/guidelines.xhtml

Supplemental table 3: LDL-cholesterol thresholds used to define control of dyslipidemia according to the Swiss Group for Lipids and Atherosclerosis.

Risk categories *	Control
High–risk	
10–y risk >20%, diabetes or personal history of CVD	<2.6 mmol/L (100 mg/dL)
Intermediate risk	
At least two CVD risk factors and 10–y risk 10% – 20%	<3.4 mmol/L (130 mg/dL)
Low risk	
At least two CVD risk factors and 10–y risk <10%	<4.2 mmol/L (160 mg/dL)
Very low risk	
Less than 2 CVD risk factors and 10–y risk <10%	<4.2 mmol/L (160 mg/dL)

* Risk calculation as defined in https://www.agla.ch/fr/calculateurs-outils/calculateur-de-risque-du-gsla. According to [5].

For peer review only

For peer review only - http://bmjopen.bmj.com/site/about/guidelines.xhtml

Supplemental table 4: list of specific SNPs interacting with the efficacy of statin treatment.

Gene	SNP	Simvastatin	Pravastatin	Atorvastatin	Rosuvastatin	Lovastatin	Fluvastatin
ABCB1	rs1045642	X, ↑		O			
ABCB1	rs1128503	↑					
ABCB1	rs1922242	O MI	O MI low	O MI low			O
ABCB1	rs2032582	↑					
ABCB1	rs3789244	O MI	O MI	O MI			
ABCG2/BCRP	rs2199936				O		
ABCG2/BCRP	rs2231137				X		
ABCG2/BCRP	rs2231142			O	X		X
ABCG8	rs11887534			O			
ACE	rs1799752						↑
APOA1	rs670		O	O			
APOA1	rs5069			O			
APOA5	rs662799	O		O		O	
APOC1	rs4420638	O	O	O	O		
APOE	rs429358	O		↑		↓	
APOE	rs445925			X			
APOE	rs449647			↑			
APOE	rs71352238				O		
APOE	rs7412		↑	↑		↓	
CETP	rs5783961						O
CETP	rs5882	O					
CETP	rs708272	O	O	O			
CLMN	rs8014194	O	O	O			
CYP2C9	rs1057910						X
CYP2D6	rs3892097			X			
CYP3A4	rs2740574			↑			
CYP3A4	rs355599367	X, ↑					
CYP3A4	rs55951658	O					
CYP3A5	rs776746	↓		X, ↓		↓	
CYP7A1	rs3808607			↓			
ESR1	rs2234693			O			
ESR1	rs9340799			-			
HMGCR	rs12916			O			
HMGCR	rs17238540		-				
HMGCR	rs17244841		↓				
HMGCR	rs17671591			X			
HMGCR	rs5908			-			
KIF6	rs20455		O	O			
LDLR	rs5925						↑
LPA	rs10455872			-	O		
LPL	rs328				O		
PCSK9	rs17111584				O		
PON1	rs662		O				
SCAP	rs12487736	↑					
SCCO1B1	rs2306283		↓		X		↑
SLCO1B1	rs11045819						↑
SLCO1B1	rs4149015		X				
SLCO1B1	rs4149056	X, ↓	X, ↓	X, ↓	X		
SOD2	rs4880				↑		
SREBF2	rs4822063			↓			
TLR4	rs4986790		O				

↑, increases effect; ↓, decreases effect; X, safety issues; O, other; MI, myocardial infarction. Data adapted from [6].

For peer review only

For peer review only - http://bmjopen.bmj.com/site/about/guidelines.xhtml

Supplemental table 5: data used to compute the genetic scores for total, LDL and HDL-cholesterol levels.

Score / Locus	Chr	SNP	Alleles (+/-)	Minor allele	β for weighting
**Total cholesterol**					
ASAP3	1	rs1077514	T/C	C	-0.030
LDLRAP1	1	rs12027135	T/A	A	-1.22
PCSK9	1	rs2479409	G/A	G	1.96
MOSC1	1	rs2807834	G/T	T	-1.38
ANGPTL3	1	rs3850634	T/G	G	-2.60
IRF2BP2	1	rs514230	T/A	A	-1.36
SORT1	1	rs629301	T/G	G	-5.41
EVI5	1	rs7515577	A/C	C	-1.18
INSIG2	2	rs10490626	G/A	A	0.042
UGT1A1	2	rs11563251	T/C	T	0.037
FAM117B	2	rs11694172	G/A	G	0.028
GCKR	2	rs1260326	T/C	T	1.91
APOB	2	rs1367117	A/G	A	4.16
LOC8431	2	rs2030746	T/C	T	0.020
ABCB11	2	rs2287623	G/A	G	0.027
ABCG5/8	2	rs4299376	G/T	G	3.01
RAB3GAP1	2	rs6759321	T/G	T	1.18
PXK	3	rs13315871	G/A	A	-0.036
RAF1	3	rs2290159	G/C	C	-1.42
CMTM6	3	rs7640978	C/T	T	-0.038
LRPAP1	4	rs6831256	G/A	G	-0.022
HMGCR	5	rs12916	C/T	C	2.84
CSNK1G3	5	rs4530754	A/G	G	-0.023
TIMD4	5	rs6882076	C/T	T	-1.98
LPA	6	rs1564348	C/T	C	2.18
HFE	6	rs1800562	G/A	A	-2.16
KCNK17	6	rs2758886	A/G	A	0.023
MYLIP	6	rs3757354	C/T	T	-1.46
HBS1L	6	rs9376090	C/T	T	-0.025
FRK	6	rs9488822	A/T	T	-1.18
GPR146	7	rs1997243	G/A	G	0.033
DNAH11	7	rs2285942	T/C	T	1.70
SOX17	8	rs10102164	A/G	A	0.030
CYP741	8	rs1030431	A/G	A	1.26
PLEC1	8	rs11136341	G/A	G	1.34
NAT2	8	rs1961456	G/A	G	1.07
PPP1R3B	8	rs2126259	C/T	T	-3.14
TRPS1	8	rs2737229	A/C	C	-1.11
TRIB1	8	rs2954022	C/A	A	-2.30
ABCA1	9	rs1883025	C/T	T	-2.24
VLDLR	9	rs3780181	A/G	G	-0.044
TTC39B	9	rs581080	C/G	G	-1.57
ABO	9	rs651007	T/C	T	2.30
VIM-CUBN	10	rs10904908	G/A	G	0.025
GPAM	10	rs2255141	A/G	A	1.14

For peer review only

MARCH8-ALOX5	10	rs970548	C/A	C	-0.026
SPTY2D1	11	rs10832963	G/T	T	-1.06
ST3GAL4	11	rs11220463	T/A	T	2.01
PHLDB1	11	rs11603023	T/C	T	0.022
FADS1-2-3	11	rs174550	T/C	C	-1.78
UBASH3B	11	rs7941030	C/T	C	0.97
APOA1	11	rs964184	G/C	G	4.68
PHC1-A2ML1	12	rs4883201	A/G	G	-0.035
LIPC	15	rs1532085	A/G	A	1.54
HPR	16	rs2000999	A/G	A	2.34
CETP	16	rs3764261	A/C	A	1.67
DLG4	17	rs314253	T/C	C	-0.023
OSBPL7	17	rs7206971	A/G	A	1.01
LIPG	18	rs7239867	G/A	A	-1.94
CILP2	19	rs10401969	T/C	C	-4.74
FLJ36070	19	rs492602	G/A	G	1.27
LDLR	19	rs6511720	G/T	T	-7.09
HNF4A	20	rs1800961	C/T	T	-4.73
ERGIC3	20	rs2277862	C/T	T	-1.19
MAFB	20	rs2902940	A/G	G	-1.38
TOP1	20	rs4297946	C/G	C	1.52
TOM1	22	rs138777	A/G	A	0.021
PPARA	22	rs4253772	T/C	T	0.032
**LDL cholesterol**					
LDLRAP1	1	rs12027135	T/A	A	-1.10
PIGV-NR0B2	1	rs12748152	T/C	T	0.05
PCSK9	1	rs2479409	G/A	G	2.01
ANXA9-CERS2	1	rs267733	A/G	G	-0.03
MOSC1	1	rs2807834	G/T	T	-1.09
ANGPTL3	1	rs3850634	T/G	G	-1.59
IRF2BP2	1	rs514230	T/A	A	-1.13
SORT1	1	rs629301	T/G	G	-5.65
INSIG2	2	rs10490626	G/A	A	-0.05
UGT1A1	2	rs11563251	T/C	T	0.03
FN1	2	rs1250229	C/T	T	-0.02
APOB	2	rs1367117	A/G	A	4.05
LOC84931	2	rs2030746	T/C	T	0.02
EHBP1	2	rs2710642	A/G	G	-0.02
ABCG5/8	2	rs4299376	G/T	G	2.75
ACAD11	3	rs17404153	G/T	T	-0.03
CMTM6	3	rs7640978	C/T	T	-0.04
LRPAP1	4	rs6831256	G/A	G	0.02
HMGCR	5	rs12916	C/T	C	2.45
CSNK1G3	5	rs4530754	A/G	G	-0.03
TIMD4	5	rs6882076	C/T	T	-1.67
FRK	6	rs11153594	C/T	T	-0.89
LPA	6	rs1564348	C/T	C	1.95
HFE	6	rs1800562	G/A	A	-2.22
MYLIP	6	rs3757354	C/T	T	-1.43
DNAH11	7	rs12670798	C/T	C	1.26
NPC1L1	7	rs217386	G/A	A	-1.17
SOX17	8	rs10102164	A/G	A	0.03

For peer review only - http://bmjopen.bmj.com/site/about/guidelines.xhtml

CYP741	8	rs1030431	A/G	A	0.95
PLEC1	8	rs11136341	G/A	G	1.40
PPP1R3B	8	rs2126259	C/T	T	-3.14
TRIB1	8	rs2954022	C/A	A	-1.84
VLDLR	9	rs3780181	A/G	G	-0.04
ABO	9	rs649129	T/C	T	2.05
GPAM	10	rs1129555	A/G	A	1.08
ST3GAL4	11	rs11220462	A/G	A	1.95
FADS1-2-3	11	rs174583	C/T	T	-1.71
APOA1	11	rs964184	G/C	G	2.85
BRCA2	13	rs4942486	T/C	T	0.02
HPR	16	rs2000999	A/G	A	2.00
CETP	16	rs247616	C/T	T	-1.45
DLG4	17	rs314253	T/C	C	-0.02
OSBPL7	17	rs7206971	A/G	A	1.01
CILP2	19	rs10401969	T/C	C	-3.11
SNX5	20	rs2328223	C/A	C	0.03
MAFB	20	rs2902941	A/G	G	-0.98
SPTLC3	20	rs364585	G/A	A	-0.03
TOP1	20	rs909802	T/C	T	1.41
PPARA	22	rs4253772	T/C	T	0.03
MTMR3	22	rs5763662	T/C	T	0.08
**HDL cholesterol**					
HDGF-PMVK	1	rs12145743	G/T	G	0.200
PIGV-NR0B2	1	rs12748152	C/T	T	-0.051
ZNF648	1	rs1689800	G/A	G	-0.47
ANGPTL1	1	rs4650994	G/A	G	0.021
PABPC4	1	rs4660293	G/A	G	-0.48
GALNT2	1	rs4846914	G/A	G	-0.61
APOB	2	rs1042034	T/C	C	0.90
COBLL1	2	rs12328675	T/C	C	0.68
IRS1	2	rs1515100	A/C	C	0.46
STAB1	3	rs13326165	A/G	A	0.029
ACAD11	3	rs17404153	G/T	T	0.028
RBM5	3	rs2013208	T/C	T	0.025
ATG7	3	rs2606736	C/T	C	0.025
GSK3B	3	rs6805251	T/C	T	0.020
C4orf52	4	rs10019888	A/G	G	-0.027
SLC39A8	4	rs13107325	T/C	T	-0.84
ADH5	4	rs2602836	A/G	A	0.019
FAM13A	4	rs3822072	G/A	A	-0.025
ARL15	5	rs6450176	A/G	A	-0.49
RSPO3	6	rs1936800	C/T	C	0.020
C6orf106	6	rs2814944	A/G	A	-0.49
CITED2	6	rs605066	C/T	C	-0.39
VEGFA	6	rs998584	C/A	A	-0.026
MLXIPL	7	rs17145738	C/T	T	0.57
TMEM176A	7	rs17173637	T/C	C	-0.036
SNX13	7	rs4142995	G/T	T	-0.026
KLF14	7	rs4731702	C/T	T	0.59
IKZF1	7	rs4917014	G/T	G	0.022
DAGLB	7	rs702485	G/A	G	0.024

For peer review only
For peer review only - http://bmjopen.bmj.com/site/about/guidelines.xhtml

TRIB1	8	rs10808546	C/T	T	0.61
LPL	8	rs12678919	A/G	G	2.25
TRPS1	8	rs2293889	T/G	T	-0.44
PPP1R3B	8	rs9987289	A/G	A	-1.21
ABCA1	9	rs1883025	T/C	T	-0.94
TTC39B	9	rs643531	C/A	C	-0.72
MARCH8-ALOX5	10	rs970548	C/A	C	0.026
OR4C46	11	rs11246602	C/T	C	0.034
KAT5	11	rs12801636	A/G	A	0.024
FADS1-2-3	11	rs174601	T/C	T	-0.73
AMPD3	11	rs2923084	G/A	G	-0.41
LRP4	11	rs3136441	T/C	C	0.78
MOGAT2-DGAT2	11	rs499974	C/A	A	-0.026
UBASH3B	11	rs7115089	C/G	G	0.31
APOA1	11	rs964184	G/C	G	-1.50
LRP1	12	rs3741414	C/T	T	0.46
SBNO1	12	rs4759375	C/T	T	0.86
ZNF664	12	rs4765127	G/T	T	0.44
PDE3A	12	rs7134375	C/A	A	0.40
MVK	12	rs7134594	C/T	C	-0.44
SCARB1	12	rs838880	T/C	C	0.61
LIPC	15	rs1532085	G/A	A	1.45
LACTB	15	rs2652834	A/G	A	-0.39
FTO	16	rs1121980	G/A	A	-0.020
LCAT	16	rs16942887	G/A	A	1.27
CMIP	16	rs2925979	T/C	T	-0.45
CETP	16	rs3764261	C/A	A	3.39
PGS1	17	rs4082919	G/T	G	-0.40
ABCA8	17	rs4148008	G/C	G	-0.42
STARD3	17	rs881844	C/G	C	-0.51
MC4R	18	rs12967135	A/G	A	-0.42
LIPG	18	rs7241918	G/T	G	-1.31
HAS1	19	rs17695224	G/A	A	-0.029
LILRA3	19	rs386000	G/C	C	0.83
ANGPTL4	19	rs7255436	C/A	C	-0.45
PEPD	19	rs731839	A/G	G	-0.022
LOC55908	19	rs737337	C/T	C	-0.64
HNF4A	20	rs1800961	T/C	T	-1.88
PLTP	20	rs6065906	C/T	C	-0.93
UBE2L3	22	rs181362	T/C	T	-0.46

Chr, chromosome; SNP, single nucleotide polymorphism. Adapted from [7].

For peer review only

For peer review only - http://bmjopen.bmj.com/site/about/guidelines.xhtml

Supplemental table 6: comparisons between retained and excluded participants, baseline (2003-2006), first (2009-2012) and second (2014-2017) follow-ups of the CoLaus|PsyCoLaus study, Lausanne, Switzerland.

	Baseline			First follow-up			Second follow-up		
	**Included**	**Excluded**	**p-value**	**Included**	**Excluded**	**p-value**	**Included**	**Excluded**	**p-value**
N	**617**	**92**		**844**	**212**		**798**	**353**	
Age (years)	61.6 ± 8.5	58.1 ± 9.6	<0.001	64.5 ± 8.8	61.7 ± 10.4	<0.001	68.1 ± 9.2	67.5 ± 10.3	0.309
Women (%)	263 (42.6)	28 (30.4)	0.027	409 (48.5)	90 (42.5)	0.117	401 (50.3)	166 (47.0)	0.313
Swiss national (%)	430 (69.7)	59 (64.1)	0.282	600 (71.1)	116 (54.7)	<0.001	563 (70.6)	191 (54.1)	<0.001
Education (%)			0.301			0.027			0.714
High	66 (10.7)	5 (5.5)		123 (14.6)	47 (22.2)		120 (15.0)	54 (15.3)	
Middle	132 (21.4)	20 (22.0)		185 (21.9)	43 (20.3)		180 (22.6)	72 (20.4)	
Low	419 (67.9)	66 (72.5)		536 (63.5)	122 (57.6)		498 (62.4)	227 (64.3)	
Married/couple (%)	428 (69.4)	65 (71.4)	0.690	505 (59.8)	121 (57.1)	0.465	448 (56.1)	166 (53.9)	0.501
BMI (kg/m^2)	28.0 ± 4.5	28.1 ± 4.8	0.792	27.6 ± 4.8	28.0 ± 5.0	0.398			
BMI categories (%)			0.979			0.986			0.197
Normal	155 (25.1)	24 (26.1)		243 (28.8)	53 (28.2)		254 (31.8)	59 (26.6)	
Overweight	277 (44.9)	41 (44.6)		378 (44.8)	85 (45.2)		327 (41.0)	91 (41.0)	
Obese	185 (30.0)	27 (29.4)		223 (26.4)	50 (26.6)		217 (27.2)	72 (32.4)	
Smoking (%)			0.064			0.099			0.067
Never	210 (34.0)	39 (42.9)		295 (35.0)	73 (34.8)		290 (36.3)	103 (38.7)	
Former	255 (41.3)	26 (28.6)		385 (45.6)	83 (39.5)		365 (45.7)	102 (38.4)	
Current	152 (24.6)	26 (28.6)		164 (19.4)	54 (25.7)		143 (17.9)	61 (22.9)	
Alcohol drinker (%)	456 (73.9)	65 (70.7)	0.510	632 (74.9)	147 (69.3)	0.101	525 (72.3)	149 (62.3)	0.004
Treatment for									
Hypertension	303 (49.1)	49 (53.3)	0.457	436 (51.7)	101 (47.6)	0.296	425 (53.3)	190 (53.8)	0.859
Diabetes	95 (15.4)	24 (26.1)	0.010	148 (17.5)	40 (18.9)	0.650	157 (19.7)	69 (19.6)	0.960
Parental history (%)	164 (26.6)	24 (26.1)	0.920	218 (25.8)	52 (24.5)	0.698	211 (26.4)	64 (18.1)	0.002

For peer review only - http://bmjopen.bmj.com/site/about/guidelines.xhtml

CVD risk (%)			0.551			0.717			<0.001
Low	267 (43.3)	36 (39.1)		367 (43.5)	89 (42.0)		356 (44.6)	103 (29.2)	
Intermediate	182 (29.5)	26 (28.3)		203 (24.1)	48 (22.6)		165 (20.7)	121 (34.3)	
High	168 (27.2)	30 (32.6)		274 (32.5)	75 (35.4)		277 (34.7)	129 (36.5)	

BMI, body mass index; CVD, cardio vascular disease. Results are expressed as number of participants (column %) for categorical variables and as average ± standard deviation for continuous variables. Between-groups comparisons performed using chi-square for categorical variables and student's t-test for continuous variables.

For peer review only

Supplemental table 7: bivariate comparison between controlled and uncontrolled participants, ESC/EAS criteria, first (2009-2012) and second (2014-2017) follow-ups of the CoLaus|PsyCoLaus study, Lausanne, Switzerland. Analysis restricted to participants taking statins.

	First follow-up			Second follow-up		
	**Uncontrolled**	**Controlled**	**p-value**	**Uncontrolled**	**Controlled**	**p-value**
N	**373**	**288**		**428**	**370**	
Generation			0.004			0.212
First	99 (26.5)	50 (17.4)		54 (16.5)	32 (12.2)	
Second	249 (66.8)	204 (70.8)		233 (71.0)	188 (71.8)	
Third	25 (6.7)	34 (11.8)		41 (12.5)	42 (16.0)	
Potency §			0.018			0.018
Low	99 (26.5)	50 (17.4)		54 (16.5)	32 (12.2)	
Intermediate	130 (34.9)	109 (37.9)		107 (32.6)	66 (25.2)	
High	144 (38.6)	129 (44.8)		167 (50.9)	164 (62.6)	
N	**348**	**274**		**328**	**262**	
Potency †			0.911			0.067
Low	166 (47.7)	126 (46.0)		117 (40.6)	72 (31.2)	
Intermediate	153 (44.0)	124 (45.3)		125 (43.4)	111 (48.0)	
High	29 (8.3)	24 (8.7)		46 (16.0)	48 (20.8)	

§, not considering posology; †, considering posology. Results are expressed as number of participants (column %). Between-groups comparisons performed using chi-square.

For peer review only

For peer review only - http://bmjopen.bmj.com/site/about/guidelines.xhtml

Supplemental table 8: bivariate comparisons between controlled and uncontrolled participants, GSLA criterion, baseline (2003-2006), first (2009-2012) and second (2014-2017) follow-ups of the CoLaus|PsyCoLaus study, Lausanne, Switzerland.

	Baseline			First follow-up			Second follow-up		
	**Uncontrolled**	**Controlled**	**p-value**	**Uncontrolled**	**Controlled**	**p-value**	**Uncontrolled**	**Controlled**	**p-value**
N	**184**	**433**		**266**	**578**		**139**	**659**	
Age (years)	61.5 ± 8.5	61.7 ± 8.5	0.728	64.5 ± 8.6	64.5 ± 8.9	0.947	67.1 ± 9.0	68.4 ± 9.3	0.139
Women (%)	81 (44.0)	182 (42.0)	0.648	119 (44.7)	290 (50.2)	0.142	72 (51.8)	329 (49.9)	0.688
Swiss national (%)	128 (70.0)	302 (69.6)	0.929	183 (69.1)	417 (72.0)	0.378	105 (74.5)	458 (69.7)	0.261
Education (%)			0.474			0.707			0.354
High	47 (10.9)	66 (10.7)		16 (6.0)	38 (6.6)		10 (7.2)	50 (7.6)	
Middle	98 (22.6)	132 (21.4)		39 (14.7)	89 (15.4)		17 (12.2)	53 (8.0)	
Low	288 (66.5)	419 (67.9)		50 (18.8)	125 (21.6)		23 (16.6)	95 (14.4)	
Married/couple (%)	127 (69.0)	301 (69.5)	0.903	353 (61.1)	505 (59.8)	0.279	74 (53.2)	374 (56.8)	0.448
BMI (kg/m^2)	28.4 ± 4.4	27.8 ± 4.5	0.150	28.2 ± 4.9	27.4 ± 4.7	0.038	28.6 ± 4.8	27.4 ± 4.9	0.008
BMI categories (%)			0.354			0.125			0.033
Normal	40 (21.7)	115 (26.6)		69 (25.9)	174 (30.1)		33 (23.7)	221 (33.5)	
Overweight	83 (45.1)	194 (44.8)		115 (43.2)	263 (45.5)		58 (41.7)	269 (40.8)	
Obese	61 (33.2)	124 (28.6)		82 (30.8)	141 (24.4)		48 (34.5)	169 (25.6)	
Smoking (%)			0.285			0.019			0.110
Never	152 (35.1)	210 (34.0)		218 (37.7)	295 (35.0)		235 (35.7)	290 (36.3)	
Former	182 (42.0)	255 (41.3)		259 (44.8)	385 (45.6)		312 (47.3)	365 (45.7)	
Current	99 (22.9)	152 (24.6)		101 (17.5)	164 (19.4)		112 (17.0)	143 (17.9)	
Alcohol drinker (%)	148 (80.4)	308 (71.1)	0.016	207 (77.8)	425 (73.5)	0.182	93 (74.4)	432 (71.9)	0.567
Treatment for									
Hypertension	98 (53.3)	205 (47.3)	0.179	41 (44.1)	395 (52.6)	0.121	79 (56.8)	346 (52.5)	0.352
Diabetes	36 (19.6)	59 (13.6)	0.061	13 (14.0)	135 (18.0)	0.339	34 (24.5)	123 (18.7)	0.118
Parental history (%)	134 (72.8)	319 (73.7)	0.828	69 (25.9)	149 (25.8)	0.960	94 (67.6)	493 (74.8)	0.081

For peer review only - http://bmjopen.bmj.com/site/about/guidelines.xhtml

CVD risk (%)			<0.001			<0.001			<0.001
Low	32 (17.4)	234 (54.0)		47 (17.7)	319 (55.2)		28 (20.1)	329 (49.9)	
Intermediate	70 (38.0)	114 (26.3)		74 (27.8)	130 (22.5)		36 (25.9)	131 (19.9)	
High	82 (44.6)	85 (19.6)		145 (54.5)	129 (22.3)		75 (54.0)	199 (30.2)	
Statins (%)	161 (87.5)	417 (96.3)	<0.001	200 (75.2)	461 (79.8)	0.134	88 (63.3)	504 (76.5)	0.001
Fibrates (%)	28 (15.2)	17 (3.9)	<0.001	17 (6.4)	16 (2.8)	0.012	7 (5.0)	25 (3.8)	0.497
Other (%)	7 (3.8)	15 (3.5)	0.835	55 (20.7)	96 (16.6)	0.152	46 (33.1)	123 (18.7)	<0.001
Number of drugs									
Including OTC	-	-		4 [3 - 6]	4 [2 - 6]	0.229 §	5 [3 - 7]	5 [3 - 7]	0.842 §
Excluding OTC	-	-		4 [2 - 6]	3 [2 - 5]	0.246 §	4 [2 - 6]	4 [2 - 6]	0.642 §
Genetic risk scores									
Total cholesterol	-2.7 ± 9.4	-3.7 ± 9.6	0.254	-3.7 ± 9.5	-3.5 ± 8.9	0.763	-3.3 ± 9.4	-3.8 ± 8.9	0.562
LDL-cholesterol	-2.4 ± 7.5	-2.9 ± 7.5	0.461	-2.5 ± 7.6	-2.4 ± 7.2	0.847	-2.5 ± 7.3	-2.9 ± 7.0	0.467
HDL-cholesterol	-6.5 ± 3.4	-6.7 ± 3.6	0.649	0.2 ± 7.4	-6.6 ± 3.5	0.312	-6.9 ± 3.8	-6.7 ± 3.6	0.539

BMI, body mass index; CVD, cardio vascular disease; OTC, over the counter. Results are expressed as number of participants (column %) for categorical variables and as average ± standard deviation or as median [interquartile range] for continuous variables. Between-groups comparisons performed using chi-square for categorical variables and student's t-test or Kruskal-Wallis nonparametric test (§) for continuous variables.

For peer review only

For peer review only - http://bmjopen.bmj.com/site/about/guidelines.xhtml

Supplemental table 9: multivariate analysis of the factors associated with dyslipidemia control, GSLA criterion, baseline (2003-2006), first (2009-2012) and second (2014-2017) follow-ups of the CoLaus|PsyCoLaus study, Lausanne, Switzerland.

	**Baseline**		**First follow-up**		**Second follow-up**	
	**OR (95% CI)**	**p-value**	**OR (95% CI)**	**p-value**	**OR (95% CI)**	**p-value**
Age (per 10 years increase)	1.17 (0.90 - 1.51)	0.231	1.05 (0.85 - 1.30)	0.627	1.18 (0.90 - 1.54)	0.221
Man vs. woman	1.70 (1.08 - 2.69)	0.022	1.15 (0.80 - 1.66)	0.458	1.59 (1.00 - 2.53)	0.050
Swiss vs. Non-Swiss	1.26 (0.81 - 1.96)	0.298	1.30 (0.90 - 1.86)	0.159	0.74 (0.45 - 1.21)	0.232
Education						
High	1 (ref.)		1 (ref.)		1 (ref.)	
Middle	1.23 (0.58 - 2.62)	0.586	1.12 (0.64 - 1.96)	0.690	0.44 (0.21 - 0.92)	0.030
Low	1.07 (0.55 - 2.05)	0.848	0.81 (0.49 - 1.32)	0.396	0.61 (0.30 - 1.22)	0.163
p-value for trend	0.848		0.396		0.163	
Married vs. not married	1.11 (0.71 - 1.72)	0.652	1.13 (0.81 - 1.59)	0.466	0.81 (0.52 - 1.26)	0.349
Body mass index categories						
Normal	1 (ref.)		1 (ref.)		1 (ref.)	
Overweight	0.75 (0.45 - 1.25)	0.268	0.92 (0.60 - 1.40)	0.691	0.57 (0.33 - 0.98)	0.043
Obese	1.05 (0.60 - 1.86)	0.854	0.96 (0.59 - 1.54)	0.854	0.58 (0.31 - 1.09)	0.091
p-value for trend	0.854		0.691		0.091	
Smoking categories						
Never	1 (ref.)		1 (ref.)		1 (ref.)	
Former	1.28 (0.78 - 2.08)	0.328	0.91 (0.62 - 1.35)	0.646	1.96 (1.19 - 3.23)	0.008
Current	1.29 (0.73 - 2.26)	0.380	0.91 (0.56 - 1.47)	0.692	1.76 (0.93 - 3.32)	0.081
p-value for trend	0.380		0.692		0.081	
Alcohol drinker (yes vs. no)	0.46 (0.28 - 0.77)	0.003	0.75 (0.50 - 1.12)	0.155	0.74 (0.45 - 1.23)	0.246
Antihypertensive treatment (yes vs. no)	1.50 (0.97 - 2.29)	0.065	1.48 (1.03 - 2.12)	0.036	1.04 (0.64 - 1.69)	0.862
Parental history (yes vs. no)	0.99 (0.61 - 1.59)	0.964	0.96 (0.65 - 1.42)	0.847	0.65 (0.40 - 1.06)	0.086
CVD risk (GSLA criterion)						
Low	1 (ref.)		1 (ref.)		1 (ref.)	

For peer review only

For peer review only - http://bmjopen.bmj.com/site/about/guidelines.xhtml

Intermediate	0.16 (0.09 - 0.28)	<0.001	0.19 (0.12 - 0.31)	<0.001	0.22 (0.11 - 0.42)	<0.001
High	0.08 (0.05 - 0.14)	<0.001	0.08 (0.05 - 0.13)	<0.001	0.100 (0.06 - 0.20)	<0.001
p-value for trend	<0.001		<0.001		<0.001	
LDL genetic risk score quartiles						
First	1 (ref.)		1 (ref.)		1 (ref.)	
Second	0.89 (0.50 - 1.58)	0.681	1.07 (0.67 - 1.69)	0.784	1.99 (1.06 - 3.74)	0.033
Third	0.54 (0.31 - 0.96)	0.034	1.11 (0.70 - 1.77)	0.645	0.93 (0.51 - 1.70)	0.815
Fourth	0.74 (0.42 - 1.30)	0.293	1.12 (0.70 - 1.77)	0.641	0.77 (0.43 - 1.38)	0.388
p-value for trend	0.125		0.617		0.108	
Hypolipidemic drug treatment						
Statins	0.35 (0.03 - 4.26)	0.411	1.19 (0.66 - 2.15)	0.570	2.21 (1.14 - 4.28)	0.019
Fibrates	0.06 (0.01 - 0.63)	0.019	0.29 (0.11 - 0.76)	0.012	0.61 (0.2 - 1.86)	0.389
Other	0.80 (0.28 - 2.27)	0.674	0.45 (0.25 - 0.81)	0.008	0.47 (0.25 - 0.89)	0.020

OR, odds ratio; CI, confidence interval. Results are expressed as odds ratio and (95% confidence interval). Statistical analysis using logistic regression.

For peer review only

For peer review only - http://bmjopen.bmj.com/site/about/guidelines.xhtml

Supplemental table 10: bivariate comparison of statin generation and potency between controlled and uncontrolled participants, GSLA criterion, first (2009-2012) and second (2014-2017) follow-ups of the CoLaus|PsyCoLaus study, Lausanne, Switzerland. Analysis restricted to participants taking statins.

	**First follow-up**			**Second follow-up**		
	**Uncontrolled**	**Controlled**	**p-value**	**Uncontrolled**	**Controlled**	**p-value**
N	**200**	**461**		**88**	**502**	
Generation			0.003			0.026
First	62 (31.0)	87 (18.9)		21 (23.9)	65 (13.0)	
Second	122 (61.0)	331 (71.8)		55 (62.5)	366 (72.9)	
Third	16 (8.0)	43 (9.3)		12 (13.6)	71 (14.1)	
Potency §			0.002			0.005
Low	62 (31.0)	87 (18.9)		21 (23.9)	65 (13.0)	
Intermediate	70 (35.0)	169 (36.7)		30 (34.1)	143 (28.5)	
High	68 (34.0)	205 (44.5)		37 (42.1)	294 (58.6)	
N	**186**	**436**		**77**	**442**	
Potency §			0.386			0.199
Low	94 (50.5)	198 (45.4)		34 (44.2)	155 (35.1)	
Intermediate	75 (40.3)	202 (46.3)		28 (36.4)	208 (47.1)	
High	17 (9.1)	36 (8.3)		15 (19.5)	79 (17.9)	

§, not considering posology; †, considering posology. Results are expressed as number of participants (column %). Between-groups comparisons performed using chi-square.

For peer review only - http://bmjopen.bmj.com/site/about/guidelines.xhtml

Supplemental table 11: multivariate analysis of the factors associated with dyslipidemia control, GSLA criterion, first (2009-2012) and second (2014-2017) follow-ups of the CoLaus|PsyCoLaus study, Lausanne, Switzerland.

	First follow-up		Second follow-up	
	**OR (95% CI)**	**p-value**	**OR (95% CI)**	**p-value**
Age (per 10 years increase)	1.20 (0.93 - 1.56)	0.159	1.04 (0.73 - 1.48)	0.823
Man vs. woman	1.59 (1.03 - 2.45)	0.037	2.23 (1.23 - 4.02)	0.008
Swiss vs. Non-Swiss	1.25 (0.81 - 1.92)	0.315	0.80 (0.43 - 1.47)	0.468
Education				
High	1 (ref.)		1 (ref.)	
Middle	1.09 (0.54 - 2.19)	0.805	0.85 (0.32 - 2.22)	0.733
Low	0.83 (0.46 - 1.52)	0.551	1.13 (0.46 - 2.74)	0.791
p-value for trend	0.551		0.791	
Married vs. not married	1.24 (0.84 - 1.84)	0.287	0.97 (0.55 - 1.71)	0.917
Body mass index categories				
Normal	1 (ref.)		1 (ref.)	
Overweight	0.87 (0.53 - 1.45)	0.603	0.61 (0.29 - 1.28)	0.191
Obese	0.86 (0.49 - 1.50)	0.591	0.47 (0.21 - 1.06)	0.068
p-value for trend	0.591		0.068	
Smoking categories				
Never	1 (ref.)		1 (ref.)	
Former	0.91 (0.58 - 1.44)	0.688	1.79 (0.96 - 3.36)	0.068
Current	0.96 (0.54 - 1.72)	0.891	1.22 (0.56 - 2.66)	0.625
p-value for trend				
Alcohol drinker (yes vs. no)	0.67 (0.41 - 1.08)	0.099	0.95 (0.51 - 1.78)	0.882
AntiHTA ttt (yes vs. no)	1.19 (0.77 - 1.84)	0.438	1.11 (0.60 - 2.05)	0.733
Parental history (yes vs. no)	0.98 (0.61 - 1.58)	0.945	0.54 (0.28 - 1.03)	0.060
CVD risk (GSLA criterion)				
Low	1 (ref.)		1 (ref.)	
Intermediate	0.18 (0.10 - 0.32)	<0.001	0.19 (0.07 - 0.49)	0.001
High	0.05 (0.03 - 0.10)	<0.001	0.06 (0.02 - 0.14)	<0.001
p-value for trend	<0.001		<0.001	
LDL genetic risk score quartiles				
First	1 (ref.)		1 (ref.)	
Second	1.04 (0.61 - 1.76)	0.888	2.95 (1.29 - 6.72)	0.010
Third	1.21 (0.70 - 2.08)	0.492	1.06 (0.51 - 2.19)	0.881
Fourth	1.29 (0.75 - 2.20)	0.359	1.18 (0.57 - 2.47)	0.653
p-value for trend	0.298		0.666	
Number of drugs (per 1 unit)	1.14 (1.05 - 1.25)	0.003	1.16 (1.04 - 1.29)	0.008
Statins generation				
First	1 (ref.)		1 (ref.)	
Second	2.54 (1.61 - 4.01)	<0.001	4.17 (2.07 - 8.42)	<0.001
Third	2.59 (1.19 - 5.61)	0.016	3.44 (1.31 - 8.99)	0.012

For peer review only - http://bmjopen.bmj.com/site/about/guidelines.xhtml

For peer review only

p-value for trend	0.016		0.012	
Fibrates	NC		NC	
Other	0.62 (0.27 - 1.43)	0.263	1.09 (0.36 - 3.3)	0.880

OR, odds ratio; CI, confidence interval; antiHTA ttt, antihypertensive drug treatment; NC, not computable. Results are expressed as odds ratio and (95% confidence interval). Statistical analysis by logistic regression.

For peer review only

Supplemental table 12: multivariate analysis of the factors associated with dyslipidemia control, GSLA criterion, first (2009-2012) and second (2014-2017) follow-ups of the CoLaus|PsyCoLaus study, Lausanne, Switzerland.

	First follow-up		Second follow-up	
	OR (95% CI)	p-value	OR (95% CI)	p-value
Age (per 10 years increase)	1.22 (0.94 - 1.59)	0.127	1.06 (0.74 - 1.51)	0.748
Man vs. woman	1.57 (1.02 - 2.43)	0.040	2.13 (1.17 - 3.87)	0.013
Swiss vs. Non-Swiss	1.24 (0.81 - 1.92)	0.322	0.82 (0.45 - 1.52)	0.533
Education				
High	1 (ref.)		1 (ref.)	
Middle	1.07 (0.53 - 2.14)	0.856	0.77 (0.29 - 2.04)	0.601
Low	0.83 (0.45 - 1.50)	0.531	1.05 (0.43 - 2.58)	0.914
p-value for trend	0.531		0.914	
Married vs. not married	1.26 (0.85 - 1.86)	0.259	1.00 (0.57 - 1.76)	0.995
Body mass index categories				
Normal	1 (ref.)		1 (ref.)	
Overweight	0.89 (0.54 - 1.47)	0.637	0.64 (0.31 - 1.35)	0.245
Obese	0.85 (0.49 - 1.49)	0.570	0.49 (0.22 - 1.10)	0.085
p-value for trend	0.570		0.085	
Smoking categories				
Never	1 (ref.)		1 (ref.)	
Former	0.90 (0.57 - 1.42)	0.653	1.83 (0.97 - 3.46)	0.061
Current	0.96 (0.54 - 1.72)	0.895	1.18 (0.53 - 2.58)	0.688
p-value for trend	0.895		0.688	
Alcohol drinker (yes vs. no)	0.67 (0.42 - 1.09)	0.106	0.90 (0.48 - 1.70)	0.750
AntiHTA ttt (yes vs. no)	1.20 (0.77 - 1.85)	0.417	1.09 (0.59 - 2.02)	0.781
Parental history (yes vs. no)	0.98 (0.61 - 1.58)	0.942	0.52 (0.27 - 0.99)	0.049
CVD risk				
Low	1 (ref.)		1 (ref.)	
Intermediate	0.17 (0.10 - 0.31)	<0.001	0.20 (0.08 - 0.51)	0.001
High	0.05 (0.03 - 0.10)	<0.001	0.06 (0.02 - 0.15)	<0.001
p-value for trend	<0.001		<0.001	
LDL genetic risk score quartiles				
First	1 (ref.)		1 (ref.)	
Second	1.05 (0.62 - 1.79)	0.852	2.74 (1.20 - 6.24)	0.016
Third	1.19 (0.70 - 2.05)	0.519	1.01 (0.49 - 2.10)	0.982
Fourth	1.27 (0.74 - 2.18)	0.380	1.12 (0.53 - 2.35)	0.763
p-value for trend	0.329		0.586	
Number of drugs (per 1 unit)	1.14 (1.04 - 1.25)	0.003	1.15 (1.03 - 1.28)	0.012
Statins potency, no posology				
Low	1 (ref.)		1 (ref.)	
Intermediate	2.14 (1.30 - 3.54)	0.003	2.90 (1.35 - 6.24)	0.007
High	3.01 (1.82 - 4.98)	<0.001	5.13 (2.43 - 10.8)	<0.001

For peer review only - http://bmjopen.bmj.com/site/about/guidelines.xhtml

p-value for trend	<0.001		<0.001	
Fibrates	NC		NC	
Other	0.58 (0.25 - 1.34)	0.202	0.91 (0.30 - 2.76)	0.871

OR, odds ratio; CI, confidence interval; antiHTA ttt, antihypertensive drug treatment; NC, not computable. Results are expressed as odds ratio and (95% confidence interval). Statistical analysis

For peer review only

Supplemental table 13: multivariate analysis of the factors associated with dyslipidemia control, GSLA criterion, first (2009-2012) and second (2014-2017) follow-ups of the CoLaus|PsyCoLaus study, Lausanne, Switzerland.

	**First follow-up**		**Second follow-up**	
	**OR (95% CI)**	**p-value**	**OR (95% CI)**	**p-value**
Age (per 10 years increase)	1.25 (0.96 - 1.63)	0.102	1.10 (0.76 - 1.60)	0.619
Man vs. woman	1.61 (1.04 - 2.51)	0.034	1.82 (0.97 - 3.42)	0.062
Swiss vs. Non-Swiss	1.25 (0.80 - 1.94)	0.321	0.81 (0.42 - 1.54)	0.515
Education				
High	1 (ref.)		1 (ref.)	
Middle	1.08 (0.53 - 2.19)	0.839	0.47 (0.16 - 1.40)	0.175
Low	0.80 (0.43 - 1.46)	0.465	0.65 (0.23 - 1.79)	0.402
p-value for trend	0.465		0.402	
Married vs. not married	1.33 (0.89 - 2.00)	0.163	1.06 (0.59 - 1.92)	0.842
Body mass index categories				
Normal	1 (ref.)		1 (ref.)	
Overweight	0.86 (0.51 - 1.43)	0.554	0.66 (0.31 - 1.40)	0.283
Obese	0.87 (0.49 - 1.55)	0.641	0.65 (0.28 - 1.48)	0.302
p-value for trend	0.641		0.302	
Smoking categories				
Never	1 (ref.)		1 (ref.)	
Former	0.95 (0.60 - 1.52)	0.836	1.72 (0.89 - 3.34)	0.109
Current	1.05 (0.58 - 1.89)	0.868	1.20 (0.52 - 2.78)	0.672
p-value for trend	0.868		0.672	
Alcohol drinker (yes vs. no)	0.67 (0.41 - 1.10)	0.113	0.93 (0.48 - 1.8)	0.831
AntiHTA ttt (yes vs. no)	1.35 (0.86 - 2.11)	0.187	1.20 (0.63 - 2.28)	0.575
Parental history (yes vs. no)	0.94 (0.58 - 1.52)	0.805	0.67 (0.34 - 1.33)	0.251
CVD risk				
Low	1 (ref.)		1 (ref.)	
Intermediate	0.19 (0.10 - 0.34)	<0.001	0.23 (0.09 - 0.59)	0.002
High	0.06 (0.03 - 0.11)	<0.001	0.07 (0.03 - 0.18)	<0.001
p-value for trend	<0.001		<0.001	
LDL genetic score quartiles				
First	1 (ref.)		1 (ref.)	
Second	1.07 (0.62 - 1.85)	0.800	1.89 (0.81 - 4.39)	0.141
Third	1.34 (0.77 - 2.34)	0.299	0.83 (0.38 - 1.79)	0.634
Fourth	1.25 (0.72 - 2.17)	0.422	1.00 (0.46 - 2.17)	0.997
p-value for trend	0.313		0.514	
Number of drugs (per 1 unit)	1.11 (1.01 - 1.21)	0.028	1.16 (1.02 - 1.30)	0.019
Statins potency, posology				
Low	1 (ref.)		1 (ref.)	
Intermediate	1.36 (0.91 - 2.06)	0.137	1.66 (0.88 - 3.11)	0.115
High	1.67 (0.81 - 3.45)	0.168	2.07 (0.89 - 4.82)	0.092

For peer review only - http://bmjopen.bmj.com/site/about/guidelines.xhtml

For peer review only

p-value for trend	0.168		0.092	
Fibrates	NC		NC	
Other	0.75 (0.31 - 1.84)	0.529	1.64 (0.43 - 6.28)	0.473

OR, odds ratio; CI, confidence interval; antiHTA ttt, antihypertensive drug treatment; NC, not computable. Results are expressed as odds ratio and (95% confidence interval). Statistical analysis by logistic regression.

For peer review only

Supplemental table 14: p-values for the different SNPs associated with statin effect regarding dyslipidemia control, according to the GSLA strict control, the GSLA near control and the ESC/EAS criteria, CoLaus|PsyCoLaus study, Lausanne, Switzerland.

		GSLA strict control			GSLA near control			ESC/EAS		
**Gene**	**SNP**	**Baseline**	**1st FU**	**2nd FU**	**Baseline**	**First FU**	**2nd FU**	**Baseline**	**First FU**	**2nd FU**
ABCB1	rs1128503	0.969	0.794	0.167	0.481	0.539	0.513	0.728	0.896	**0.040**
ABCB1	rs1922242	0.648	0.360	**0.026**	0.902	0.598	0.474	0.910	0.369	0.147
ABCB1	rs2032582	0.887	0.673	0.150	0.387	0.553	0.454	0.835	0.492	0.156
ABCB1	rs3789244	0.985	0.779	0.167	0.481	0.539	0.513	0.728	0.901	**0.040**
ABCG2/BCRP	rs2199936	0.649	0.381	0.141	0.452	0.295	0.507	0.485	0.861	0.444
ABCG2/BCRP	rs2231137	1.000	0.817	0.906	0.671	0.897	0.584	0.094	0.966	0.292
ABCG2/BCRP	rs2231142	0.592	0.310	0.141	0.452	0.329	0.507	0.411	0.894	0.444
ABCG8	rs11887534	0.945	0.210	0.196	1.000	**0.031**	0.603	0.541	0.671	**0.002**
APOA1	rs5069	0.072	0.245	1.000	0.501	0.219	0.680	0.232	0.858	1.000
APOA1	rs670	0.735	0.378	0.386	0.456	0.372	0.833	0.793	0.314	0.125
APOA5	rs662799	0.712	0.792	0.125	0.396	0.437	1.000	0.238	0.052	0.092
APOC1	rs4420638	0.514	0.697	0.085	0.092	0.208	0.503	0.847	0.089	0.567
APOE	rs429358	0.308	0.576	0.784	0.105	1.000	1.000	1.000	0.077	0.502
APOE	rs445925	1.000	0.141	0.856	0.414	0.606	1.000	0.718	0.623	0.788
APOE	rs449647	0.919	0.870	0.617	0.708	0.642	1.000	0.461	0.888	0.207
APOE	rs71352238	0.358	0.952	0.642	0.056	0.761	0.791	0.856	0.269	0.489
APOE	rs7412	0.792	0.694	0.803	0.702	0.347	1.000	0.246	0.154	0.145
CETP	rs5882	0.446	0.773	0.211	0.371	0.105	0.319	0.683	0.749	0.932
CETP	rs708272	0.593	0.580	0.247	0.785	0.182	0.453	0.088	0.841	0.459
CLMN	rs8014194	0.339	0.543	0.636	0.138	0.154	0.527	0.304	0.477	0.382
CYP2C9	rs1057910	0.392	0.060	0.163	0.393	0.064	0.302	0.798	0.064	0.060
CYP2D6	rs3892097	0.328	0.641	0.601	0.619	0.798	0.242	0.781	0.540	0.800
CYP3A4	rs2740574	0.805	0.729	0.220	0.165	0.473	0.703	0.394	0.305	0.430
CYP3A4	rs55951658	NC	NC	NC	NC	NC	NC	NC	NC	NC

For peer review only - http://bmjopen.bmj.com/site/about/guidelines.xhtml

For peer review only

CYP7A1	rs3808607	**0.017**	0.153	0.601	0.647	0.149	0.766	**0.043**	0.573	0.267
ESR1	rs2234693	0.613	0.868	0.119	0.816	0.797	0.757	**0.026**	0.938	1.000
ESR1	rs9340799	0.420	0.778	0.156	**0.036**	0.390	0.619	0.139	0.899	0.569
HMGCR	rs12916	0.584	0.912	0.651	0.802	0.139	0.413	0.598	0.406	0.626
HMGCR	rs17238540	0.153	0.901	0.537	1.000	0.450	0.430	0.833	0.552	0.713
HMGCR	rs17244841	0.151	1.000	0.536	1.000	0.442	0.424	0.666	0.498	0.705
HMGCR	rs17671591	0.337	0.388	0.634	0.590	0.124	0.737	0.171	0.947	0.158
HMGCR	rs5908	1.000	1.000	0.466	0.271	1.000	1.000	0.102	0.507	0.632
KIF6	rs20455	0.602	0.323	0.609	0.094	0.124	0.058	0.307	**0.029**	0.209
LDLR	rs5925	0.206	0.281	0.348	0.553	0.889	0.935	0.408	0.311	**0.015**
LPA	rs10455872	**0.021**	0.340	1.000	1.000	0.461	0.452	0.266	0.659	0.332
LPL	rs328	0.186	0.654	0.835	0.087	1.000	0.878	0.372	0.397	0.674
PCSK9	rs17111584	1.000	0.197	1.000	0.380	**0.027**	1.000	0.462	0.359	0.054
PON1	rs662	0.130	0.407	0.684	0.730	1.000	0.749	0.069	0.637	0.911
SCAP	rs12487736	0.226	0.525	0.840	**0.032**	0.711	0.413	**0.031**	0.145	0.312
SCCO1B1	rs2306283	0.533	0.404	0.902	0.719	0.557	0.126	0.076	0.429	0.384
SLCO1B1	rs11045819	**0.020**	0.841	0.250	0.466	**0.044**	0.728	0.914	0.768	0.068
SLCO1B1	rs4149015	0.895	0.084	0.303	0.758	0.438	**0.039**	0.572	0.819	0.808
SLCO1B1	rs4149056	0.675	0.574	**0.027**	0.856	**0.045**	0.356	0.112	0.888	**0.011**
SOD2	rs4880	0.182	0.740	0.837	0.228	0.925	0.214	0.255	0.611	0.460
TLR4	rs4986790	0.727	0.124	0.662	1.000	0.166	1.000	0.654	0.873	**0.013**
ABCB1	rs1045642	0.877	0.160	0.350	0.461	0.578	0.269	0.789	0.500	0.619

NC, not computable as all participants were homozygous. FU, follow-up. Statistical analysis performed using Fisher's exact test. Values <0.05 are indicated in bold.

For peer review only - http://bmjopen.bmj.com/site/about/guidelines.xhtml

References

1. Maji D, Shaikh S, Solanki D, et al. Safety of statins. *Indian J Endocrinol Metab* 2013; 17: 636-646. 2013/08/21. DOI: 10.4103/2230-8210.113754.
2. Stone NJ, Robinson JG, Lichtenstein AH, et al. 2013 ACC/AHA guideline on the treatment of blood cholesterol to reduce atherosclerotic cardiovascular risk in adults: a report of the American College of Cardiology/American Heart Association Task Force on Practice Guidelines. *J Am Coll Cardiol* 2014; 63: 2889-2934. 2013/11/19. DOI: 10.1016/j.jacc.2013.11.002.
3. Catapano AL, Graham I, De Backer G, et al. 2016 ESC/EAS Guidelines for the Management of Dyslipidaemias. *Eur Heart J* 2016; 37: 2999-3058. 2016/08/28. DOI: 10.1093/eurheartj/ehw272.
4. Marques-Vidal P, Rodondi N, Bochud M, et al. Predictive accuracy and usefulness of calibration of the ESC SCORE in Switzerland. *Eur J Cardiovasc Prev Rehabil* 2008; 15: 402-408. 2008/08/05. DOI: 10.1097/HJR.0b013e3282fb040f.
5. Firmann M, Marques-Vidal P, Paccaud F, et al. Prevalence, treatment and control of dyslipidaemia in Switzerland: still a long way to go. *Eur J Cardiovasc Prev Rehabil* 2010; 17: 682-687. 2010/08/12. DOI: 10.1097/HJR.0b013e32833a09ab.
6. Maxwell WD, Ramsey LB, Johnson SG, et al. Impact of pharmacogenetics on efficacy and safety of statin therapy for dyslipidemia. *Pharmacotherapy* 2017; 37: 1172-1190. 2017/07/04. DOI: 10.1002/phar.1981.
7. Spracklen CN, Saftlas AF, Triche EW, et al. Genetic Predisposition to Dyslipidemia and Risk of Preeclampsia. *Am J Hypertens* 2015; 28: 915-923. 2014/12/20. DOI: 10.1093/ajh/hpu242.

For peer review only

STROBE Statement—Checklist of items that should be included in reports of ***cross-sectional studies***

	Item No	Recommendation	Page No
**Title and abstract**	1	(*a*) Indicate the study's design with a commonly used term in the title or the abstract	1
		(*b*) Provide in the abstract an informative and balanced summary of what was done and what was found	2
**Introduction**			
Background/rationale	2	Explain the scientific background and rationale for the investigation being reported	4
Objectives	3	State specific objectives, including any prespecified hypotheses	4
**Methods**			
Study design	4	Present key elements of study design early in the paper	4
Setting	5	Describe the setting, locations, and relevant dates, including periods of recruitment, exposure, follow-up, and data collection	4
Participants	6	(*a*) Give the eligibility criteria, and the sources and methods of selection of participants	4-5
Variables	7	Clearly define all outcomes, exposures, predictors, potential confounders, and effect modifiers. Give diagnostic criteria, if applicable	5
Data sources/ measurement	8*	For each variable of interest, give sources of data and details of methods of assessment (measurement). Describe comparability of assessment methods if there is more than one group	5-6
Bias	9	Describe any efforts to address potential sources of bias	7
Study size	10	Explain how the study size was arrived at	7
Quantitative variables	11	Explain how quantitative variables were handled in the analyses. If applicable, describe which groupings were chosen and why	7
Statistical methods	12	(*a*) Describe all statistical methods, including those used to control for confounding	7
		(*b*) Describe any methods used to examine subgroups and interactions	NR
		(*c*) Explain how missing data were addressed	NR
		(*d*) If applicable, describe analytical methods taking account of sampling strategy	NR
		(*e*) Describe any sensitivity analyses	7
**Results**			
Participants	13*	(a) Report numbers of individuals at each stage of study—eg numbers potentially eligible, examined for eligibility, confirmed eligible, included in the study, completing follow-up, and analysed	Sup fig 1
		(b) Give reasons for non-participation at each stage	Sup fig 1
		(c) Consider use of a flow diagram	Sup fig 1
Descriptive data	14*	(a) Give characteristics of study participants (eg demographic, clinical, social) and information on exposures and potential confounders	Sup table 6
		(b) Indicate number of participants with missing data for each variable of interest	NR
Outcome data	15*	Report numbers of outcome events or summary measures	

For peer review only - http://bmjopen.bmj.com/site/about/guidelines.xhtml

Main results	16	(*a*) Give unadjusted estimates and, if applicable, confounder-adjusted estimates and their precision (eg, 95% confidence interval). Make clear which confounders were adjusted for and why they were included	Tables 1 and 2
		(*b*) Report category boundaries when continuous variables were categorized	NR
		(*c*) If relevant, consider translating estimates of relative risk into absolute risk for a meaningful time period	NR
Other analyses	17	Report other analyses done—eg analyses of subgroups and interactions, and sensitivity analyses	Sup tables 7-13
**Discussion**			
Key results	18	Summarise key results with reference to study objectives	10
Limitations	19	Discuss limitations of the study, taking into account sources of potential bias or imprecision. Discuss both direction and magnitude of any potential bias	12-13
Interpretation	20	Give a cautious overall interpretation of results considering objectives, limitations, multiplicity of analyses, results from similar studies, and other relevant evidence	10-12
Generalisability	21	Discuss the generalisability (external validity) of the study results	12
**Other information**			
Funding	22	Give the source of funding and the role of the funders for the present study and, if applicable, for the original study on which the present article is based	14

*Give information separately for exposed and unexposed groups.

Note: An Explanation and Elaboration article discusses each checklist item and gives methodological background and published examples of transparent reporting. The STROBE checklist is best used in conjunction with this article (freely available on the Web sites of PLoS Medicine at http://www.plosmedicine.org/, Annals of Internal Medicine at http://www.annals.org/, and Epidemiology at http://www.epidem.com/). Information on the STROBE Initiative is available at www.strobe-statement.org.

For peer review only

BMJ Open

Association of clinical and genetic risk factors with management of dyslipidemia: analysis of repeated cross-sectional studies in the general population of Lausanne, Switzerland

Journal:	*BMJ Open*
Manuscript ID	bmjopen-2022-065409.R1
Article Type:	Original research
Date Submitted by the Author:	19-Jan-2023

Complete List of Authors:	Chekanova, Valeriya; National Medical Research Center of Cardiology; University of Lausanne, Department of Medicine, Internal medicine<br>Abolhassani, Nazanin; University of Lausanne, Department of Medicine, Internal medicine; Institute of Primary health care<br>Vaucher, Julien; CHUV, Medicine, Internal medicine<br>Marques-Vidal, Pedro; Centre hospitalier universitaire vaudois Departement de medecine, Department of Medicine, Internal Medicine

**Primary Subject Heading**:	Public health
Secondary Subject Heading:	Cardiovascular medicine, Epidemiology
Keywords:	Lipid disorders < DIABETES & ENDOCRINOLOGY, EPIDEMIOLOGY, GENETICS, CARDIOLOGY

SCHOLARONE™ Manuscripts

I, the Submitting Author has the right to grant and does grant on behalf of all authors of the Work (as defined in the below author licence), an exclusive licence and/or a non-exclusive licence for contributions from authors who are: i) UK Crown employees; ii) where BMJ has agreed a CC-BY licence shall apply, and/or iii) in accordance with the terms applicable for US Federal Government officers or employees acting as part of their official duties; on a worldwide, perpetual, irrevocable, royalty-free basis to BMJ Publishing Group Ltd ("BMJ") its licensees and where the relevant Journal is co-owned by BMJ to the co-owners of the Journal, to publish the Work in this journal and any other BMJ products and to exploit all rights, as set out in our licence.

The Submitting Author accepts and understands that any supply made under these terms is made by BMJ to the Submitting Author unless you are acting as an employee on behalf of your employer or a postgraduate student of an affiliated institution which is paying any applicable article publishing charge ("APC") for Open Access articles. Where the Submitting Author wishes to make the Work available on an Open Access basis (and intends to pay the relevant APC), the terms of reuse of such Open Access shall be governed by a Creative Commons licence – details of these licences and which Creative Commons licence will apply to this Work are set out in our licence referred to above.

Other than as permitted in any relevant BMJ Author's Self Archiving Policies, I confirm this Work has not been accepted for publication elsewhere, is not being considered for publication elsewhere and does not duplicate material already published. I confirm all authors consent to publication of this Work and authorise the granting of this licence.

For peer review only

Association of clinical and genetic risk factors with management of dyslipidemia: analysis of repeated cross-sectional studies in the general population of Lausanne, Switzerland

Valeriya Chekanova, MD [1, 2]; Nazanin Abolhassani, PhD [2, 3]; Julien Vaucher, MD, PhD [2] and Pedro Marques-Vidal, MD, PhD [2]

[1] National Medical Research Center of Cardiology, Moscow, Russia

[2] Department of Medicine, Internal medicine, Lausanne university hospital and university of Lausanne, Lausanne, Switzerland

[3] Institute of Primary health care (BIHAM), Bern, Switzerland

Authors' emails

Valeriya Chekanova:	2466107@mail.ru
Nazanin Abolhassani:	Nazanin.Abolhassani@biham.unibe.ch
Julien Vaucher:	Julien.Vaucher@chuv.ch
Pedro Marques-Vidal:	Pedro.marquesvidal@gmail.com

Correspondence to:

Pedro Marques-Vidal
Office BH10-642
Internal Medicine, Lausanne University Hospital (CHUV)
Rue du Bugnon 46
1011 Lausanne
Switzerland
Phone: +41 21 314 09 34
Email: Pedro-Manuel.Marques-Vidal@chuv.ch or Pedro.marquesvidal@gmail.com

For peer review only

Abstract

Objectives: To assess the importance of clinical and genetic factors in management of dyslipidemia in the general population.

Design: Repeated cross-sectional studies (2003-2006; 2009-2012 and 2014-2017) from a population-based cohort.

Setting: Single center in Lausanne, Switzerland.

Participants: 617 (42.6% women, mean±standard deviation 61.6±8.5 years), 844 (48.5% women, 64.5±8.8 years) and 798 (50.3% women, 68.1±9.2) participants of the baseline, first and second follow-ups receiving any type of lipid-lowering drug. Participants were excluded if they had missing information regarding lipid levels, covariates, or genetic data.

Primary and secondary outcome measures: Management of dyslipidemia was assessed according to European or Swiss guidelines. Genetic risk scores (GRS) for lipid levels were computed based on the existing literature.

Results: Prevalence of adequately controlled dyslipidemia was 52%, 45% and 46% at baseline, first and second follow-ups, respectively. On multivariable analysis, when compared to intermediate or low-risk individuals, participants at very high cardiovascular risk had an odds ratio (OR) for dyslipidemia control of 0.11 (95% confidence interval, 0.06-0.18), 0.12 (0.08-0.19) and 0.38 (0.25-0.59) at baseline, first and second follow-ups, respectively. Use of newer generation or higher potency statins was associated with better control: OR 1.90 (1.18-3.05) and 3.62 (1.65-7.92) for second and third generations compared to first in the first follow-up, with the corresponding values in the second follow-up being 1.90 (1.08-3.36) and 2.18 (1.05-4.51). No differences in GRS were found between controlled and inadequately controlled subjects. Similar findings were obtained using Swiss guidelines.

Conclusion: Management of dyslipidemia is suboptimal in Switzerland. The effectiveness of high potency statins is hampered by low posology. The use of GRS in the management of dyslipidemia is not recommended.

For peer review only - http://bmjopen.bmj.com/site/about/guidelines.xhtml

Keywords: dyslipidemia, management and control; genetic risk score.

For peer review only

For peer review only - http://bmjopen.bmj.com/site/about/guidelines.xhtml

Strengths and limitations of this study

- Multiple cross-sectional studies conducted in a population-based cohort.
- Three different genetic risk scores and 51 single SNPs for lipids were tested.
- Two criteria to define and treat dyslipidemia were applied.
- Lack of consensus regarding diagnosis and management of dyslipidemia; results cannot be extrapolated to other settings and populations.
- Results based on a single population and hence not forcefully generalizable to other settings and populations.

For peer review only

For peer review only - http://bmjopen.bmj.com/site/about/guidelines.xhtml

Introduction

Adequate management of dyslipidemia (high LDL cholesterol levels) translates into a reduction in fatal and non-fatal cardiovascular disease (CVD) [1 2], and guidelines for the management of dyslipidemia have been issued by the European Society of Cardiology (ESC) and the European Atherosclerosis Society (EAS) [3]. Potent hypolipidemic drugs are available, allowing a considerable reduction in LDL-cholesterol levels [3]. Still, management of dyslipidemia is suboptimal, with a significant percentage of treated patients not reaching target levels [4]. Likely contributing factors are inadequate perception of risk by physicians [5], low compliance by patients [6] or use of lesser potent drugs [4]. It has also been suggested that the efficacy of statins, the main hypolipidemic drugs used, could be modulated by the genetic background of the patients [7 8]. A recent review suggested that several single nucleotide polymorphisms (SNPs) could be associated with a reduction in the efficacy of statin treatment [8]. Still, the effect of genetic markers on the management of dyslipidemia in the general population has seldom been established.

Thus, we aimed to assess the importance of clinical and genetic factors in the management of dyslipidemia using data from a population-based cohort.

Methods

Study population

The CoLaus|PsyCoLaus (www.colaus-psycolaus.ch) is a prospective cohort study following every 5 years a sample of the inhabitants of the city of Lausanne (Switzerland, population 137,810 in 2017), aged 35 to 75 years at baseline [9]. In the present study, data from the baseline (2003-2006), the first (2009-2012) and the second (2014-2017) follow-ups were used.

Inclusion and exclusion criteria

Participants were eligible if they received any type of lipid-lowering drug. Participants were initially excluded if they had missing information regarding lipid levels, covariates, or genetic data.

For peer review only

Lipid-lowering treatment and control of dyslipidemia

At each survey, participants reported which drugs they were taking. Based on the Anatomical Therapeutic Chemical (ATC) classification system of the WHO, participants were considered as being treated for dyslipidemia if they were taking one drug coded C10 ("Lipid modifying agents"). Lipid-lowering drugs were further classified into statins, fibrates, and other lipid-lowering drugs. For statins, a further classification regarding the generation and potency was performed in the first and second follow-ups (**supplemental table 1**). Such classification could not be achieved in the baseline survey due to limited coding. Two approaches regarding statin potency were conducted: 1) not taking into account and 2) taking into account posology as defined by US guidelines [10]. This last approach is similar to another study conducted in Poland [11].

As there is no consensus regarding CVD risk assessment in Switzerland, two approaches were applied. The first approach used the ESC/EAS guidelines [3] by applying the SCORE equation recalibrated for Switzerland [12] (**supplemental table 2**). Three CVD categories were defined: very high, high and other. The second approach used the Swiss Group for Lipids and Atherosclerosis (GSLA) criteria [13] (**supplemental table 3**). Depending on the risk category, the threshold to define adequate control changed (**supplemental tables 2 and 3**).

Genetic analysis and genetic scores

Genome-wide genotyping was performed using the Affymetrix 500K SNP array. Subjects were excluded from the analysis in case of inconsistency between sex and genetic data, a genotype call rate of less than 90%, or inconsistencies of genotyping results in duplicate samples. Quality control for SNPs was performed using the following criteria: monomorphic (or with minor allele frequency (MAF) <1%), call rates less than 90%, deviation from the Hardy-Weinberg equilibrium (HWE) ($p<1x10^{-6}$). Phased haplotypes were generated using SHAPEIT2 [14]. Imputation was performed using minimac3 and the Haplotype Reference Consortium version r1.1. Fifty-one SNPs associated with lipid-lowering drug efficiency were extracted (**supplemental table 4**) from a previous review [8]. Genetic risk scores (GRS) for total, LDL- and HDL-cholesterol were computed using 223 SNPs overall as suggested previously

For peer review only - http://bmjopen.bmj.com/site/about/guidelines.xhtml

[15]. Briefly, the GRS were calculated with each SNP being weighted by its relative effect size (β coefficient) obtained from the literature (**supplemental table 5**).

Other covariates

Socio-demographic and lifestyle data were collected by questionnaire and included gender, age, educational level (low/middle/high), marital status (alone/couple), personal and family history of CVD, family history of dyslipidemia, smoking (never/former/current) and alcohol consumption (yes/no). Number of other drugs (including or excluding non-prescribed, over-the-counter drugs) was considered as a proxy for the number of comorbidities.

Body weight and height were measured with participants barefoot and in light indoor clothes. Body weight was measured in kilograms to the nearest 100 g using a Seca® scale (Hamburg, Germany). Height was measured to the nearest 5 mm using a Seca® (Hamburg, Germany) height gauge. Body mass index (BMI) was calculated and categorized into normal (<25 kg/m^2), overweight (25≤BMI<30 kg/m^2) and obese (BMI≥30 kg/m^2).

Blood pressure (BP) was measured using an Omron® HEM-907 automated oscillometric sphygmomanometer after at least a 10-minute rest in a seated position, and the average of the last two measurements was used. Hypertension was defined by a systolic blood pressure (SBP) ≥140 mm Hg or a diastolic blood pressure (DBP) ≥90 mm Hg or presence of antihypertensive drug treatment.

Eight-hour fasting blood samples were collected, and biological measurements were conducted in a Modular P apparatus (Roche Diagnostics, Basel, Switzerland) for the baseline and first follow-up, and in a Cobas 8000 (Roche Diagnostics, Basel, Switzerland) device for the second follow-up. The following analytical procedures (with maximum inter and intra-batch CVs) were used: total cholesterol by CHOD-PAP (1.6-1.7%) and HDL-cholesterol by CHOD-PAP + PEG + cyclodextrin (3.6-0.9%). Glucose was assessed by glucose dehydrogenase (2.1-1.0%) at baseline and by glucose hexokinase (1.6-0.8%) at first and second follow-ups. Diabetes was defined as fasting plasma glucose ≥7.0 mmol/L or presence of an antidiabetic drug treatment.

For peer review only - http://bmjopen.bmj.com/site/about/guidelines.xhtml

Statistical analysis

Statistical analyses were conducted using Stata v.16.1 (Stata Corp, College Station, TX, USA) separately for each survey. Results were expressed as number of participants (percentage) for categorical variables and as average±standard deviation or median [interquartile range] for continuous variables. Bivariate comparisons between controlled and uncontrolled participants (using either ESC/EAS or GSLA criteria) were performed using chi-square for categorical variables and Student's t-test or Kruskal-Wallis nonparametric test for continuous variables. Multivariable analyses were conducted using logistic regression for categorical variables and results were expressed as multivariable-adjusted odds ratio (OR) and 95% confidence interval (CI).

The associations between specific SNPs and management of dyslipidemia were assessed by comparing the distribution of the genotypes according to controlled and uncontrolled participants (as defined by ESC/EAS or GSLA criteria) using Fisher's exact test.

Statistical significance was considered for a two-sided test with $p<0.05$.

Patient and public involvement

None.

Results

Prevalence of dyslipidemia and changes in statin category

Overall, there were 709, 1056 and 1151 eligible participants at baseline, first and second follow-ups, respectively, of whom 92 (13.0%), 212 (20.1%) and 353 (30.7%) were excluded, leaving 617, 844 and 798 participants for analysis. The reasons for exclusion are indicated in **supplemental figure 1**; the main reason was lack of genetic data. The number of participants treated for dyslipidemia changed between surveys depending on the number of participants newly treated and the number of participants who dropped out. The characteristics of the included and the excluded participants are summarized in **supplemental table 6**; excluded participants were less frequently born in Switzerland, while no other consistent difference was found.

For peer review only - http://bmjopen.bmj.com/site/about/guidelines.xhtml

The distribution of the different types of lipid-lowering treatments for the three surveys is provided in **supplemental figure 2**, and of the statin generations and potency for the first and second follow-ups are provided in **supplemental figure 3**. Statins represented the first type of hypolipidemic drug, but their predominance decreased with time. Prevalence of first generation statins decreased and prevalence of third generation statins increased. Prevalence of low potency statins decreased and high potency statins increased. When posology was considered, statin potency was considerably reduced, but trends were similar (**supplemental figure 3**). This decrease in potency was most marked for intermediate potency statins (**supplemental figure 4**).

Prevalence and factors associated with control of dyslipidemia, ESC/EAS criteria

Prevalence of adequately managed dyslipidemia was 52%, 45% and 46% at baseline, first and second follow-ups, respectively. The results of the analysis using the ESC/EAS criteria stratified by survey are summarized in **tables 1 to 4**.

On bivariate analysis (**table 1**), controlled participants were younger, had lower levels of cardiovascular risk factors and CVD risk, and a higher prevalence of parental history of CVD than inadequately controlled participants in all surveys. Controlled participants also had a lower BMI and were taking less drugs than inadequately controlled participants in the first and second follow-ups; prevalence of fibrates was higher among inadequately controlled participants at baseline and in the first follow-up. No differences were found regarding GRS between controlled and inadequately controlled participants in all surveys. On multivariable analysis (**table 2**), increased age or CVD risk was negatively associated with control in all surveys; no association was found between type of hypolipidemic drug or quartiles of the LDL GRS and dyslipidemia control.

For peer review only - http://bmjopen.bmj.com/site/about/guidelines.xhtml

Table 1. Bivariate comparison of socio-economic and clinical characteristics among participants treated for dyslipidemia, according to controlled and uncontrolled status as per European Society of Cardiology / European Atherosclerosis Society (ESC/EAS) criteria. Data from the baseline (2003-2006), first (2009-2012) and second (2014-2017) follow-ups of the CoLaus|PsyCoLaus study, Lausanne, Switzerland.

	**Baseline**			**First follow-up**			**Second follow-up**		
	**Uncontrolled**	**Controlled**	**p-value**	**Uncontrolled**	**Controlled**	**p-value**	**Uncontrolled**	**Controlled**	**p-value**
N	**295**	**322**		**465**	**379**		**428**	**370**	
Age (years)	64.0 ± 8.4	59.5 ± 8.0	<0.001	67.4 ± 8.6	61.0 ± 7.7	<0.001	71.4 ± 8.2	64.4 ± 8.9	<0.001
Women (%)	118 (40.1)	145 (44.9)	0.233	186 (40.0)	223 (58.8)	<0.001	218 (50.9)	183 (49.5)	0.678
Swiss national (%)	210 (71.4)	220 (68.1)	0.371	339 (72.9)	261 (68.9)	0.198	312 (72.9)	251 (67.8)	0.118
Education (%)			0.917			0.512			0.049
High	31 (10.5)	35 (10.8)		62 (13.3)	61 (16.1)		52 (12.2)	68 (18.4)	
Middle	61 (20.8)	71 (22.0)		102 (21.9)	83 (21.9)		99 (23.1)	81 (21.9)	
Low	202 (68.7)	217 (67.2)		301 (64.7)	235 (62.0)		277 (64.7)	221 (59.7)	
Married/couple (%)	199 (67.7)	229 (70.9)	0.388	271 (58.3)	234 (61.7)	0.308	233 (54.4)	215 (58.1)	0.298
BMI (kg/m^2)	28.2 ± 4.4	27.8 ± 4.6	0.217	28.1 ± 4.6	27.1 ± 4.9	0.002	28.1 ± 4.6	27.0 ± 5.1	0.002
BMI categories (%)			0.250			0.001			0.001
Normal	65 (22.1)	90 (27.9)		111 (23.9)	132 (34.8)		112 (26.2)	142 (38.4)	
Overweight	136 (46.3)	141 (43.7)		216 (46.5)	162 (42.7)		185 (43.2)	142 (38.4)	
Obese	93 (31.6)	92 (28.5)		138 (29.7)	85 (22.4)		131 (30.6)	86 (23.2)	
Smoking (%)			0.714			0.239			0.151
Never	101 (34.4)	109 (33.8)		153 (32.9)	142 (37.5)		168 (39.3)	122 (33.0)	
Former	117 (39.8)	138 (42.7)		224 (48.2)	161 (42.5)		190 (44.4)	175 (47.3)	
Current	76 (25.9)	76 (23.5)		88 (18.9)	76 (20.1)		70 (16.4)	73 (19.7)	
Alcohol drinker (%)	224 (76.2)	232 (71.8)	0.218	352 (75.7)	280 (73.9)	0.544	285 (73.3)	240 (71.2)	0.538
Treatment for (%)									
Hypertension	158 (53.7)	145 (44.9)	0.028	266 (57.2)	170 (44.9)	<0.001	258 (60.3)	167 (45.1)	<0.001
Diabetes	68 (23.1)	27 (8.4)	<0.001	121 (26.0)	27 (7.1)	<0.001	102 (23.8)	55 (14.9)	0.001

For peer review only - http://bmjopen.bmj.com/site/about/guidelines.xhtml

Parental history (%)	63 (21.4)	101 (31.3)	0.006	95 (20.4)	123 (32.5)	<0.001	95 (22.2)	116 (31.4)	0.003
CVD risk (%)			<0.001			<0.001			<0.001
Other	95 (32.3)	173 (53.6)		140 (30.1)	225 (59.4)		162 (37.9)	193 (52.2)	
High	66 (22.5)	118 (36.5)		98 (21.1)	107 (28.2)		73 (17.1)	94 (25.4)	
Very high	133 (45.2)	32 (9.9)		227 (48.8)	47 (12.4)		193 (45.1)	83 (22.4)	
Number of drugs									
Including OTC	-	-		4 [3 - 6]	4 [2 - 5]	<0.001*	5 [3 - 7]	4 [3 - 6]	<0.001*
Excluding OTC	-	-		4 [2 - 6]	3 [2 - 5]	<0.001*	4 [3 - 7]	3 [2 - 6]	<0.001*
Genetic risk scores									
Total cholesterol	-2.8 ± 9.4	-3.9 ± 9.7	0.149	-3.5 ± 9.2	-3.6 ± 8.9	0.941	-4.2 ± 9.6	-3.2 ± 8.1	0.147
LDL-cholesterol	-2.2 ± 7.8	-3.2 ± 7.2	0.117	-2.5 ± 7.5	-2.3 ± 7.1	0.827	-3.2 ± 7.6	-2.5 ± 6.3	0.171
HDL-cholesterol	-6.5 ± 3.5	-6.8 ± 3.4	0.215	-6.6 ± 3.6	-6.9 ± 3.6	0.371	-6.5 ± 3.6	-6.9 ± 3.6	0.124
Hypolipidemic drug treatment (%)									
Statins	270 (91.5)	308 (95.7)	0.035	373 (80.4)	288 (75.8)	0.107	328 (76.6)	264 (71.4)	0.089
Fibrates	28 (9.5)	17 (5.3)	0.044	28 (6.0)	5 (1.3)	<0.001	18 (4.2)	14 (3.8)	0.762
Other	11 (3.7)	11 (3.4)	0.834	72 (15.5)	79 (20.8)	0.047	84 (19.6)	85 (23.0)	0.249

BMI, body mass index; CVD, cardiovascular disease; OTC, over the counter. Results are expressed as number of participants (column %) for categorical variables and as average ± standard deviation or as median [interquartile range] for continuous variables. Between-groups comparisons performed using chi-square for categorical variables and student's t-test or Kruskal-Wallis nonparametric test (*) for continuous variables.

For peer review only - http://bmjopen.bmj.com/site/about/guidelines.xhtml

For peer review only

Table 2. Multivariable analysis of the factors associated with dyslipidemia control as per European Society of Cardiology / European Atherosclerosis Society criteria. Data from the baseline (2003-2006), first (2009-2012) and second (2014-2017) follow-ups of the CoLaus|PsyCoLaus study, Lausanne, Switzerland.

	Baseline		First follow-up		Second follow-up	
	**OR (95% CI)**	**p-value**	**OR (95% CI)**	**p-value**	**OR (95% CI)**	**p-value**
Age (per 10 years increase)	0.44 (0.34 - 0.57)	<0.001	0.34 (0.27 - 0.43)	<0.001	0.35 (0.28 - 0.44)	<0.001
Man vs. woman	0.89 (0.58 - 1.36)	0.599	0.48 (0.34 - 0.70)	<0.001	1.23 (0.85 - 1.79)	0.273
Swiss vs. Non-Swiss	1.30 (0.85 - 1.97)	0.223	1.13 (0.79 - 1.64)	0.503	1.01 (0.69 - 1.48)	0.948
Education						
High	1 (ref.)		1 (ref.)		1 (ref.)	
Middle	1.11 (0.54 - 2.27)	0.782	0.83 (0.48 - 1.45)	0.521	0.61 (0.35 - 1.07)	0.087
Low	1.11 (0.59 - 2.11)	0.738	0.81 (0.49 - 1.33)	0.407	0.65 (0.40 - 1.07)	0.093
p-value for trend	0.738		0.407		0.093	
Married vs. not married	1.21 (0.80 - 1.82)	0.374	1.21 (0.86 - 1.71)	0.270	1.06 (0.74 - 1.5)	0.759
Body mass index categories						
Normal	1 (ref.)		1 (ref.)		1 (ref.)	
Overweight	0.59 (0.37 - 0.94)	0.028	0.85 (0.57 - 1.26)	0.413	0.61 (0.40 - 0.92)	0.019
Obese	0.92 (0.54 - 1.56)	0.753	0.96 (0.59 - 1.55)	0.852	0.64 (0.40 - 1.04)	0.074
p-value for trend	0.753		0.852		0.074	
Smoking categories						
Never	1 (ref.)		1 (ref.)		1 (ref.)	
Former	1.54 (0.98 - 2.40)	0.059	1.10 (0.75 - 1.62)	0.618	1.38 (0.94 - 2.02)	0.099
Current	0.63 (0.36 - 1.08)	0.094	0.85 (0.52 - 1.39)	0.512	1.04 (0.61 - 1.75)	0.892
p-value for trend	0.094		0.512		0.892	
Alcohol drinker (yes vs. no)	0.66 (0.42 - 1.03)	0.068	0.91 (0.62 - 1.35)	0.645	0.64 (0.43 - 0.94)	0.024
Antihypertensive treatment (yes vs. no)	1.05 (0.70 - 1.57)	0.820	1.18 (0.82 - 1.69)	0.368	0.77 (0.53 - 1.12)	0.176
Parental history (yes vs. no)	1.00 (0.64 - 1.56)	0.998	1.08 (0.74 - 1.59)	0.690	0.79 (0.53 - 1.18)	0.256
CVD risk						

For peer review only - http://bmjopen.bmj.com/site/about/guidelines.xhtml

Other	1 (ref.)		1 (ref.)		1 (ref.)	
High	1.25 (0.76 - 2.04)	0.379	0.68 (0.45 - 1.04)	0.074	1.42 (0.88 - 2.28)	0.153
Very high	0.11 (0.06 - 0.18)	<0.001	0.12 (0.08 - 0.19)	<0.001	0.38 (0.25 - 0.59)	<0.001
p-value for trend	<0.001		<0.001		<0.001	
LDL genetic risk score quartiles						
First	1 (ref.)		1 (ref.)		1 (ref.)	
Second	1.11 (0.65 - 1.89)	0.696	0.97 (0.61 - 1.54)	0.883	1.31 (0.81 - 2.12)	0.264
Third	0.70 (0.41 - 1.19)	0.185	0.96 (0.60 - 1.53)	0.866	1.57 (0.97 - 2.51)	0.064
Fourth	0.74 (0.43 - 1.25)	0.259	1.07 (0.67 - 1.72)	0.764	1.44 (0.89 - 2.32)	0.133
p-value for trend	0.107		0.781		0.099	
Hypolipidemic drug treatment						
Statins	1.56 (0.18 - 13.8)	0.690	1.00 (0.55 - 1.81)	0.998	1.42 (0.81 - 2.51)	0.223
Fibrates	0.67 (0.08 - 5.35)	0.707	0.13 (0.04 - 0.43)	0.001	1.20 (0.46 - 3.15)	0.714
Other	0.94 (0.34 - 2.61)	0.910	0.57 (0.31 - 1.04)	0.067	0.71 (0.40 - 1.26)	0.237

OR, odds ratio; CI, confidence interval; CVD, cardiovascular disease. Results are expressed as odds ratio and (95% CI). Statistical analysis using logistic regression.

For peer review only - http://bmjopen.bmj.com/site/about/guidelines.xhtml

The distribution of the statin generation or potency according to dyslipidemia control is provided in **supplemental table 7**. Controlled participants had a higher prevalence of third generation (first follow up) or high potency statins than inadequately controlled participants. When posology was used to estimate potency, no differences were found. The results of the multivariable analyses taking into account statin generation or statin potency irrespective of the posology are provided in **tables 3 and supplemental table 8**, respectively. In both analyses, increasing age or CVD risk led to a lower likelihood of being controlled, while increasing statin generation or potency led to a higher likelihood of being controlled. When posology was used to estimate potency, the association was no longer significant (supplemental **table 9**).

For peer review only

For peer review only - http://bmjopen.bmj.com/site/about/guidelines.xhtml

Table 3. Multivariable analysis of the factors associated with dyslipidemia control as per European Society of Cardiology / European Atherosclerosis Society criteria. Data from the first (2009-2012) and second (2014-2017) follow-ups of the CoLaus|PsyCoLaus study, Lausanne, Switzerland. Analysis taking into account statin generation.

	First follow-up		Second follow-up	
	**OR (95% CI)**	**p-value**	**OR (95% CI)**	**p-value**
Age (per 10 years increase)	0.31 (0.23 - 0.41)	<0.001	0.35 (0.27 - 0.47)	<0.001
Man vs. woman	0.60 (0.39 - 0.92)	0.018	1.40 (0.90 - 2.16)	0.134
Swiss vs. Non-Swiss	1.17 (0.76 - 1.80)	0.474	1.16 (0.75 - 1.80)	0.502
Education				
High	1 (ref.)		1 (ref.)	
Middle	0.78 (0.40 - 1.53)	0.476	1.08 (0.54 - 2.15)	0.821
Low	0.88 (0.49 - 1.59)	0.681	1.04 (0.57 - 1.90)	0.895
p-value for trend	0.681		0.895	
Married vs. not married	1.23 (0.82 - 1.83)	0.317	1.19 (0.79 - 1.78)	0.407
Body mass index categories				
Normal	1 (ref.)		1 (ref.)	
Overweight	0.84 (0.52 - 1.35)	0.474	0.56 (0.35 - 0.92)	0.023
Obese	0.91 (0.53 - 1.58)	0.749	0.54 (0.31 - 0.95)	0.032
p-value for trend	0.749		0.032	
Smoking categories				
Never	1 (ref.)		1 (ref.)	
Former	1.09 (0.70 - 1.71)	0.695	1.21 (0.78 - 1.88)	0.384
Current	0.84 (0.46 - 1.51)	0.560	0.93 (0.51 - 1.71)	0.820
p-value for trend	0.560		0.820	
Alcohol drinker (yes vs. no)	0.79 (0.50 - 1.25)	0.316	0.72 (0.46 - 1.12)	0.146
AntiHTA ttt (yes vs. no)	0.97 (0.63 - 1.51)	0.903	0.80 (0.52 - 1.25)	0.337
Parental history (yes vs. no)	1.27 (0.80 - 2.02)	0.310	0.76 (0.48 - 1.23)	0.267
CVD risk				
Other	1 (ref.)		1 (ref.)	
High	0.63 (0.39 - 1.02)	0.061	1.32 (0.76 - 2.31)	0.327
Very high	0.08 (0.05 - 0.14)	<0.001	0.35 (0.21 - 0.58)	<0.001
p-value for trend	<0.001		<0.001	
LDL genetic risk score quartiles				
First	1 (ref.)		1 (ref.)	
Second	0.90 (0.53 - 1.54)	0.707	1.67 (0.95 - 2.93)	0.076
Third	0.89 (0.52 - 1.52)	0.665	1.79 (1.04 - 3.07)	0.036
Fourth	1.11 (0.65 - 1.92)	0.696	1.64 (0.93 - 2.86)	0.085
p-value for trend	0.725		0.085	
Number of drugs (per 1 unit)	1.15 (1.05 - 1.25)	0.002	1.07 (0.99 - 1.15)	0.069
Statin generation				
First	1 (ref.)		1 (ref.)	
Second	1.90 (1.18 - 3.05)	0.008	1.90 (1.08 - 3.36)	0.026

For peer review only - http://bmjopen.bmj.com/site/about/guidelines.xhtml

Third	3.62 (1.65 - 7.92)	0.001	2.18 (1.05 - 4.51)	0.036
p-value for trend	0.001		0.036	
Fibrates	NC		2.55 (0.19 - 34.1)	0.480
Other hypolipidemic drugs	0.90 (0.38 - 2.11)	0.800	1.13 (0.51 - 2.51)	0.762

OR, odds ratio; CI, confidence interval; antiHTA ttt, antihypertensive drug treatment; NC, not computable. Results are expressed as odds ratio and (95% confidence interval). Statistical analysis using logistic regression.

Prevalence and factors associated with control of dyslipidemia, GSLA criteria

Prevalence of adequately managed dyslipidemia was 70%, 68% and 83% in the baseline, first and second follow-ups, respectively. The results of the analysis using the GSLA criteria stratified by survey are summarized in **supplemental tables 10 to 15**.

On bivariate analysis, controlled participants had lower CVD risk (all surveys), lower BMI (first and second follow-ups) and lower prevalence of smoking (first follow-up) than inadequately controlled participants; no differences were found regarding GRS (**supplemental table 10**). On multivariable analysis, increased CVD risk was negatively associated with dyslipidemia control in all surveys; men had a higher likelihood of being controlled (baseline and second follow-up) and alcohol consumption decreased likelihood of control (baseline); no association was found with LDL GRS or the class of hypolipidemic drug (**supplemental table 11**).

The distribution of the statin generation or potency according to dyslipidemia control is provided in **supplemental table 12**. Controlled participants had a higher prevalence of third generation or high potency statins than inadequately controlled participants. When posology was used to estimate potency, no differences were found. The results of the multivariable analyses taking into account statin generation or statin potency irrespective of the posology are provided in **supplemental tables 13 and 14**, respectively. In both analyses, increasing CVD risk led to a lower likelihood of being adequately controlled, while being male, increasing number of drugs or increasing statin generation or potency led a higher likelihood of being adequately controlled. When posology was used to estimate statin potency, the association was no longer significant (**supplemental table 15**).

For peer review only - http://bmjopen.bmj.com/site/about/guidelines.xhtml

Specific SNPs

The p-values for the associations between 51 specific SNPs and dyslipidemia control are presented in **supplemental table 16**. Most statistically significant associations were found for *SLCO1B1* (Solute Carrier Organic Anion Transporter Family Member 1B1), but no consistent association was found overall.

Discussion

Our results show that individuals at high risk of CVD present an increased risk of mismanagement of dyslipidemia, which was consistent among the three survey periods. The use of more potent statins increased the likelihood of dyslipidemia control, while lipid GRS were not associated with dyslipidemia control.

Prevalence of controlled dyslipidemia

Prevalence of controlled dyslipidemia varied between 45% and 52% according to ESC/EAS criteria and between 68% and 83% according to GSLA criteria. Those values are higher than the values reported by the EUROASPIRE IV, where a third of participants (32.7%) achieved the target level of <2.5 mmol/L for LDL cholesterol [16]. Still, comparisons are difficult as EUROASPIRE IV focused on high-risk participants only. Importantly, prevalence rates of adequately managed dyslipidemia were much higher using GSLA than ESC/EAS criteria. Hence, a clinician using GSLA criteria will lower LDL levels to a lesser level than using ESC/EAS criteria. Given that CVD risk decreases linearly with LDL cholesterol levels (all other factors being equal) [17], the decrease in CVD risk is expected to be lower using GSLA than using ESC/EAS criteria. It would be important to evaluate if people managed according to the GSLA criteria achieve the same level of protection against CVD as if they were managed according to the ESC/EAS criteria.

Factors associated with controlled dyslipidemia

Participants at high risk of CVD had a higher likelihood of being inadequately managed in all surveys, irrespective of the criteria considered. Those findings are consistent with a recent review of European

For peer review only - http://bmjopen.bmj.com/site/about/guidelines.xhtml

studies [18] and a large cross-sectional European study [19] where control rates were lower than 20%. Possible explanations include the fact that subjects at high CVD risk should have much lower lipid values, thus more difficult to achieve.

Similar to a Polish study [11] but contrary to a US study [20], the prevalence of highly potent statins increased from 41% in 2009-2012 to 56% in 2014-2017. This value is higher than reported in the EUROASPIRE V [4] study, where 49.9% of participants were on high intensity therapy. Importantly, participants on high potency statins achieved better control, a finding also reported in the EUROASPIRE V [4] and the DA VINCI [19] studies. The 2019 ESC/EAS guidelines for management of dyslipidemia recommend that high potency statins at highest recommended tolerable dose be initially applied to control lipid levels [3]. Our results thus strengthen the importance of such recommendation, and GPs should be urged to shift to more potent statins to achieve better results. Still, our results also suggest that, despite a higher prescription rate of highly potent statins, those drugs are not prescribed at their full potency/posology, and that a sizable fraction of treated subjects fails to reach lipid targets.

Other clinical and socio-demographic factors were associated with dyslipidemia control, but associations were inconsistent between study periods or between ESC/EAS and GSLA criteria. Increasing age was negatively associated with dyslipidemia control using ESC/EAS criteria but no association was found using GSLA criteria. Either no association [21], an inverse association [22], or a positive association [23] between age and dyslipidemia control have been reported. Similarly, men achieved better control than women using the GSLA criteria, while no consistent association was found using the ESC/EAS criteria. Better control rates have also been reported in the USA [24], while the inverse association was reported in Germany [22]. Such discrepancies might be related to the criteria applied, as age and gender might be stronger or weaker determinants of CVD risk in some risk equations compared to others. No association was found between nationality, education, marital status, job type or BMI categories and dyslipidemia control. Our findings replicate those of other studies where no association between education [21], marital status [21 22] and dyslipidemia control

For peer review only - http://bmjopen.bmj.com/site/about/guidelines.xhtml

was found. Overall, our results suggest that the socio-demographic factors associated with dyslipidemia control differ according to country and to the criteria used to estimate CVD risk.

Genetic scores and individual SNPs

Several authors suggested that genetic profiling could be used to guide statin treatment and thus improve outcomes [7 8]. A meta-analysis published in 2015 concluded that people with the highest burden of genetic risk derived the largest relative and absolute clinical benefit from statin therapy [25], although such statement could also apply to people for whom cardiovascular risk was assessed using clinical data. Further, the initial promises regarding genetic testing of the kinesin-like protein 6 (KIF6) gene to guide statin prescription (the StatinCheck® test) were not confirmed [26]. In this study, no association between genetic scores for lipid markers and statin efficiency was found. Possible reasons include the small effect of each individual SNP [27], as a set of 95 SNPs explained less than 15% of total lipid variance [28], or the progressive blunting of the genetic effect by advanced aging as found for blood pressure [29]. Thus, our results suggest that genetic profiling of subjects prior to initiation of statin therapy might clinically irrelevant, and such profiling is not stated in the current ESC/EAS guidelines for the management of dyslipidemias [3]. Nevertheless, several associations were found with the *SLCO1B1* gene. Some authors have suggested that genetic variations in this gene are associated with response to statins [8]. Hence, this gene might be of interest to adapt statin treatment, and it would be important that other studies be conducted to confirm our findings.

Study limitations

This study has several limitations worth acknowledging. Firstly, the sample size was relatively small and our study was likely underpowered to detect the minute associations between the genetic scores and dyslipidemia control. Still, should those GRS be applied in clinical practice, their effect should be large enough to allow choosing between several statins in a given individual. Secondly, the analysis was restricted to Switzerland, and findings might be generalizable to other countries or ethnicities. Still, most findings agree with larger studies such as EUROASPIRE V [4] or DA VINCI [19]. Thirdly, there is no consensus regarding the management of dyslipidemia, as thresholds for treatment vary according

For peer review only - http://bmjopen.bmj.com/site/about/guidelines.xhtml

to country or scientific society [30]. Hence, our results cannot be extrapolated to other settings, and it would be important that similar studies be conducted in other countries.

Conclusion

Management of dyslipidemia is suboptimal in Switzerland, especially for individuals at high cardiovascular risk. The effectiveness of high potency statins is hampered by low posology. GRS are not associated with dyslipidemia control, but the effect of *SLCO1B1* in statin therapy should be further investigated.

For peer review only

For peer review only - http://bmjopen.bmj.com/site/about/guidelines.xhtml

Funding

The CoLaus|PsyCoLaus study was and is supported by research grants from GlaxoSmithKline (N/A), the Faculty of Biology and Medicine of Lausanne (N/A), and the Swiss National Science Foundation (grants 33CSCO-122661, 33CS30-139468, 33CS30-148401 and 33CS30_177535/1). Valeriya Chekanova received an excellence scholarship of the Swiss government (N/A) to conduct research in Switzerland for one year.

Data availability statement

Non-identifiable individual-level data are available for researchers who seek to answer questions related to health and disease in the context of research projects who meet the criteria for data sharing by research committees. Please follow the instructions at https://www.colaus-psycolaus.ch/ for information on how to submit an application for gaining access to CoLaus|PsyCoLaus data.

Competing interests

The authors report no conflict of interest.

Contributorships

Valeriya Chekanova: Investigation, Methodology, Writing – Original draft preparation, Visualization. Pedro Marques-Vidal: Conceptualization, Methodology, Data curation, Formal analysis, Writing – Reviewing and editing, Visualization. Nazanin Abolhassani: Writing – Reviewing and editing. Julien Vaucher: Reviewing and editing. The authors had full access to the data and took responsibility for its integrity. All authors have read and agreed to the written manuscript. PMV had full access to the data and is the guarantor of the study.

Ethics statement

The institutional Ethics Committee of the University of Lausanne, which afterwards became the Ethics Commission of Canton Vaud (www.cer-vd.ch) approved the baseline (reference 16/03), the first (reference 33/09) and the second (reference 26/14) follow-ups. The approval was confirmed in 2021

For peer review only

(reference PB_2018-00038, 239/09). The study was performed in agreement with the Helsinki declaration and its former amendments, and in accordance with the applicable Swiss legislation. All participants gave their signed informed consent before entering the study.

For peer review only

For peer review only - http://bmjopen.bmj.com/site/about/guidelines.xhtml

References

1. Cholesterol Treatment Trialists C, Fulcher J, O'Connell R, et al. Efficacy and safety of LDL-lowering therapy among men and women: meta-analysis of individual data from 174,000 participants in 27 randomised trials. *Lancet* 2015;385(9976):1397-405. doi: 10.1016/S0140-6736(14)61368-4 [published Online First: 2015/01/13]
2. Singh BM, Lamichhane HK, Srivatsa SS, et al. Role of Statins in the Primary Prevention of Atherosclerotic Cardiovascular Disease and Mortality in the Population with Mean Cholesterol in the Near-Optimal to Borderline High Range: A Systematic Review and Meta-Analysis. *Adv Prev Med* 2020;2020:6617905. doi: 10.1155/2020/6617905 [published Online First: 2020/12/10]
3. Mach F, Baigent C, Catapano AL, et al. 2019 ESC/EAS Guidelines for the management of dyslipidaemias: lipid modification to reduce cardiovascular risk. *Eur Heart J* 2020;41(1):111-88. doi: 10.1093/eurheartj/ehz455 [published Online First: 2019/09/11]
4. De Backer G, Jankowski P, Kotseva K, et al. Management of dyslipidaemia in patients with coronary heart disease: Results from the ESC-EORP EUROASPIRE V survey in 27 countries. *Atherosclerosis* 2019;285:135-46. doi: 10.1016/j.atherosclerosis.2019.03.014 [published Online First: 2019/05/06]
5. Mosca L, Linfante AH, Benjamin EJ, et al. National study of physician awareness and adherence to cardiovascular disease prevention guidelines. *Circulation* 2005;111(4):499-510. doi: 10.1161/01.CIR.0000154568.43333.82 [published Online First: 2005/02/03]
6. Naderi SH, Bestwick JP, Wald DS. Adherence to drugs that prevent cardiovascular disease: meta-analysis on 376,162 patients. *Am J Med* 2012;125(9):882-7 e1. doi: 10.1016/j.amjmed.2011.12.013 [published Online First: 2012/07/04]
7. Cano-Corres R, Candas-Estebanez B, Padro-Miquel A, et al. Influence of 6 genetic variants on the efficacy of statins in patients with dyslipidemia. *J Clin Lab Anal* 2018;32(8):e22566. doi: 10.1002/jcla.22566 [published Online First: 2018/05/08]
8. Maxwell WD, Ramsey LB, Johnson SG, et al. Impact of pharmacogenetics on efficacy and safety of statin therapy for dyslipidemia. *Pharmacotherapy* 2017;37(9):1172-90. doi: 10.1002/phar.1981 [published Online First: 2017/07/04]
9. Firmann M, Mayor V, Vidal PM, et al. The CoLaus study: a population-based study to investigate the epidemiology and genetic determinants of cardiovascular risk factors and metabolic syndrome. *BMC Cardiovasc Disord* 2008;8:6. doi: 10.1186/1471-2261-8-6 [published Online First: 2008/03/28]

For peer review only - http://bmjopen.bmj.com/site/about/guidelines.xhtml

10. Stone NJ, Robinson JG, Lichtenstein AH, et al. 2013 ACC/AHA guideline on the treatment of blood cholesterol to reduce atherosclerotic cardiovascular risk in adults: a report of the American College of Cardiology/American Heart Association Task Force on Practice Guidelines. *J Am Coll Cardiol* 2014;63(25 Pt B):2889-934. doi: 10.1016/j.jacc.2013.11.002 [published Online First: 2013/11/19]

11. Pajak A, Szafraniec K, Polak M, et al. Changes in the prevalence, treatment, and control of hypercholesterolemia and other dyslipidemias over 10 years in Poland: the WOBASZ study. *Pol Arch Med Wewn* 2016;126(9):642-52. doi: 10.20452/pamw.3464 [published Online First: 2016/07/28]

12. Marques-Vidal P, Rodondi N, Bochud M, et al. Predictive accuracy and usefulness of calibration of the ESC SCORE in Switzerland. *Eur J Cardiovasc Prev Rehabil* 2008;15(4):402-8. doi: 10.1097/HJR.0b013e3282fb040f [published Online First: 2008/08/05]

13. Moser M, Gencer B, Rodondi N. [Recommendations for management of dyslipidemia in 2014]. *Rev Med Suisse* 2014;10(420):518, 20-4. [published Online First: 2014/04/08]

14. Delaneau O, Zagury JF, Marchini J. Improved whole-chromosome phasing for disease and population genetic studies. *Nat Methods* 2013;10(1):5-6. doi: 10.1038/nmeth.2307 [published Online First: 2012/12/28]

15. Spracklen CN, Saftlas AF, Triche EW, et al. Genetic Predisposition to Dyslipidemia and Risk of Preeclampsia. *Am J Hypertens* 2015;28(7):915-23. doi: 10.1093/ajh/hpu242 [published Online First: 2014/12/20]

16. Kotseva K, De Bacquer D, De Backer G, et al. Lifestyle and risk factor management in people at high risk of cardiovascular disease. A report from the European Society of Cardiology European Action on Secondary and Primary Prevention by Intervention to Reduce Events (EUROASPIRE) IV cross-sectional survey in 14 European regions. *Eur J Prev Cardiol* 2016;23(18):2007-18. doi: 10.1177/2047487316667784 [published Online First: 2016/09/18]

17. Cholesterol Treatment Trialists C, Baigent C, Blackwell L, et al. Efficacy and safety of more intensive lowering of LDL cholesterol: a meta-analysis of data from 170,000 participants in 26 randomised trials. *Lancet* 2010;376(9753):1670-81. doi: 10.1016/S0140-6736(10)61350-5 [published Online First: 2010/11/12]

18. Bruckert E, Parhofer KG, Gonzalez-Juanatey JR, et al. Proportion of High-Risk/Very High-Risk Patients in Europe with Low-Density Lipoprotein Cholesterol at Target According to European Guidelines: A Systematic Review. *Adv Ther* 2020;37(5):1724-36. doi: 10.1007/s12325-020-01285-2 [published Online First: 2020/03/23]

For peer review only

19. Ray KK, Molemans B, Schoonen WM, et al. EU-Wide Cross-Sectional Observational Study of Lipid-Modifying Therapy Use in Secondary and Primary Care: the DA VINCI study. *Eur J Prev Cardiol* 2021;28(11):1279-89. doi: 10.1093/eurjpc/zwaa047 [published Online First: 2021/02/14]
20. Gu A, Kamat S, Argulian E. Trends and disparities in statin use and low-density lipoprotein cholesterol levels among US patients with diabetes, 1999-2014. *Diabetes Res Clin Pract* 2018;139:1-10. doi: 10.1016/j.diabres.2018.02.019 [published Online First: 2018/02/25]
21. Sozmen K, Unal B, Sakarya S, et al. Determinants of prevalence, awareness, treatment and control of high LDL-C in Turkey. *Anatol J Cardiol* 2016;16(6):370-84. doi: 10.14744/AnatolJCardiol.2016.7018 [published Online First: 2016/06/11]
22. Tiffe T, Wagner M, Rucker V, et al. Control of cardiovascular risk factors and its determinants in the general population- findings from the STAAB cohort study. *BMC Cardiovasc Disord* 2017;17(1):276. doi: 10.1186/s12872-017-0708-x [published Online First: 2017/11/04]
23. Boo S, Yoon YJ, Oh H. Evaluating the prevalence, awareness, and control of hypertension, diabetes, and dyslipidemia in Korea using the NHIS-NSC database: A cross-sectional analysis. *Medicine (Baltimore)* 2018;97(51):e13713. doi: 10.1097/MD.0000000000013713 [published Online First: 2018/12/24]
24. Peters SAE, Muntner P, Woodward M. Sex Differences in the Prevalence of, and Trends in, Cardiovascular Risk Factors, Treatment, and Control in the United States, 2001 to 2016. *Circulation* 2019;139(8):1025-35. doi: 10.1161/CIRCULATIONAHA.118.035550 [published Online First: 2019/02/20]
25. Mega JL, Stitziel NO, Smith JG, et al. Genetic risk, coronary heart disease events, and the clinical benefit of statin therapy: an analysis of primary and secondary prevention trials. *Lancet* 2015;385(9984):2264-71. doi: 10.1016/S0140-6736(14)61730-X [published Online First: 2015/03/10]
26. Ridker PM, MacFadyen JG, Glynn RJ, et al. Kinesin-like protein 6 (KIF6) polymorphism and the efficacy of rosuvastatin in primary prevention. *Circ Cardiovasc Genet* 2011;4(3):312-7. doi: 10.1161/CIRCGENETICS.110.959353 [published Online First: 2011/04/16]
27. Waterworth DM, Ricketts SL, Song K, et al. Genetic variants influencing circulating lipid levels and risk of coronary artery disease. *Arterioscler Thromb Vasc Biol* 2010;30(11):2264-76. doi: 10.1161/ATVBAHA.109.201020 [published Online First: 2010/09/25]
28. Teslovich TM, Musunuru K, Smith AV, et al. Biological, clinical and population relevance of 95 loci for blood lipids. *Nature* 2010;466(7307):707-13. doi: 10.1038/nature09270 [published Online First: 2010/08/06]

For peer review only - http://bmjopen.bmj.com/site/about/guidelines.xhtml

29. Simino J, Shi G, Bis JC, et al. Gene-age interactions in blood pressure regulation: a large-scale investigation with the CHARGE, Global BPgen, and ICBP Consortia. *Am J Hum Genet* 2014;95(1):24-38. doi: 10.1016/j.ajhg.2014.05.010 [published Online First: 2014/06/24]

30. Pavlovic J, Greenland P, Franco OH, et al. Recommendations and Associated Levels of Evidence for Statin Use in Primary Prevention of Cardiovascular Disease: A Comparison at Population Level of the American Heart Association/American College of Cardiology/Multisociety, US Preventive Services Task Force, Department of Veterans Affairs/Department of Defense, Canadian Cardiovascular Society, and European Society of Cardiology/European Atherosclerosis Society Clinical Practice Guidelines. *Circ Cardiovasc Qual Outcomes* 2021;14(9):e007183. doi: 10.1161/CIRCOUTCOMES.120.007183 [published Online First: 2021/09/22]

For peer review only

For peer review only - http://bmjopen.bmj.com/site/about/guidelines.xhtml

Supplemental figure 1: flowchart of the selection of participants, baseline (2003-2006), first (2009-2012) and second (2014-2017) follow-ups of the CoLaus|PsyCoLaus study, Lausanne, Switzerland

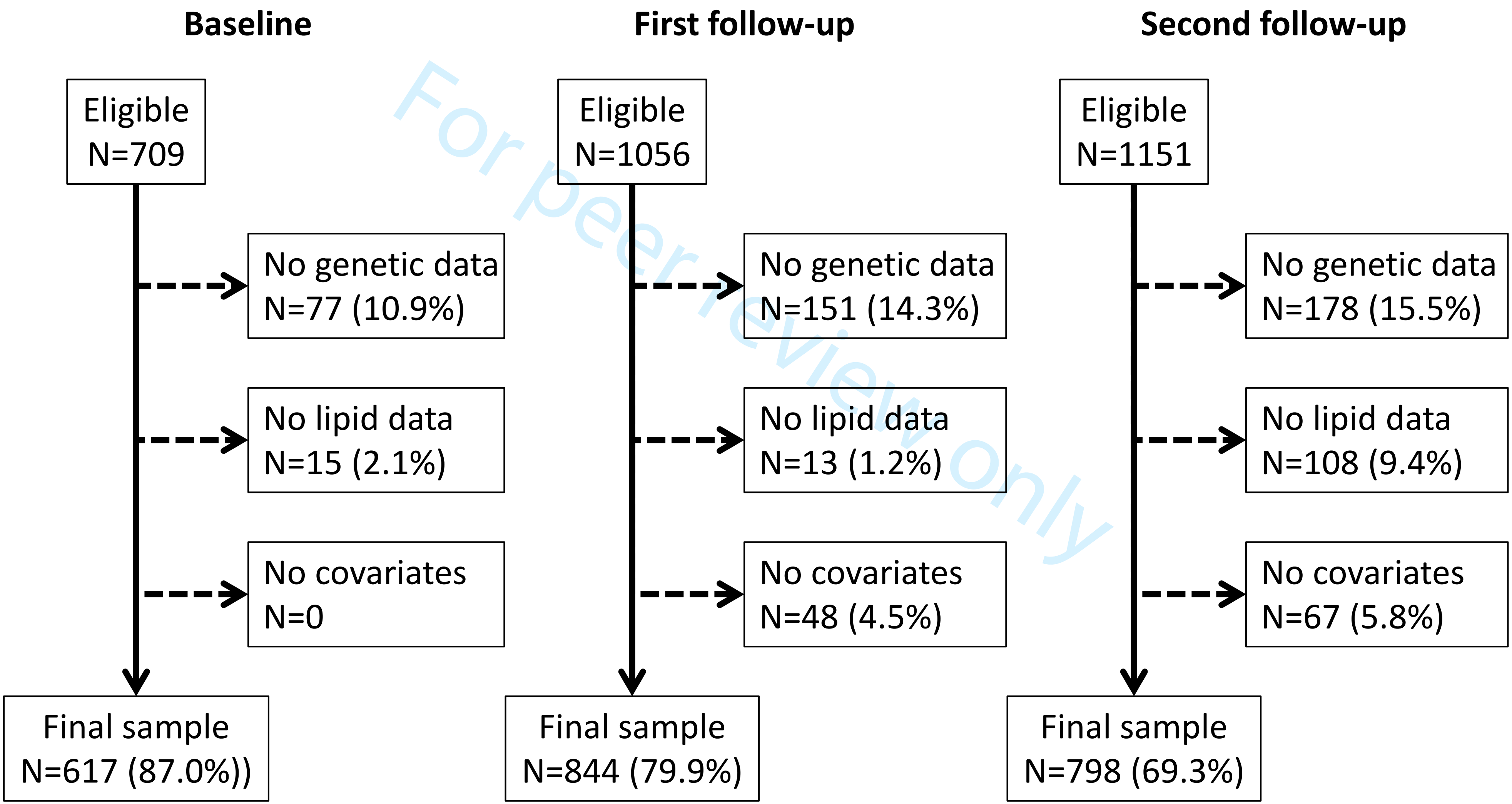

For peer review only

Supplemental figure 2: distribution of hypolipidemic drug classes, baseline (2003-2006), first (2009-2012) and second (2014-2017) follow-ups of the CoLaus|PsyCoLaus study, Lausanne, Switzerland

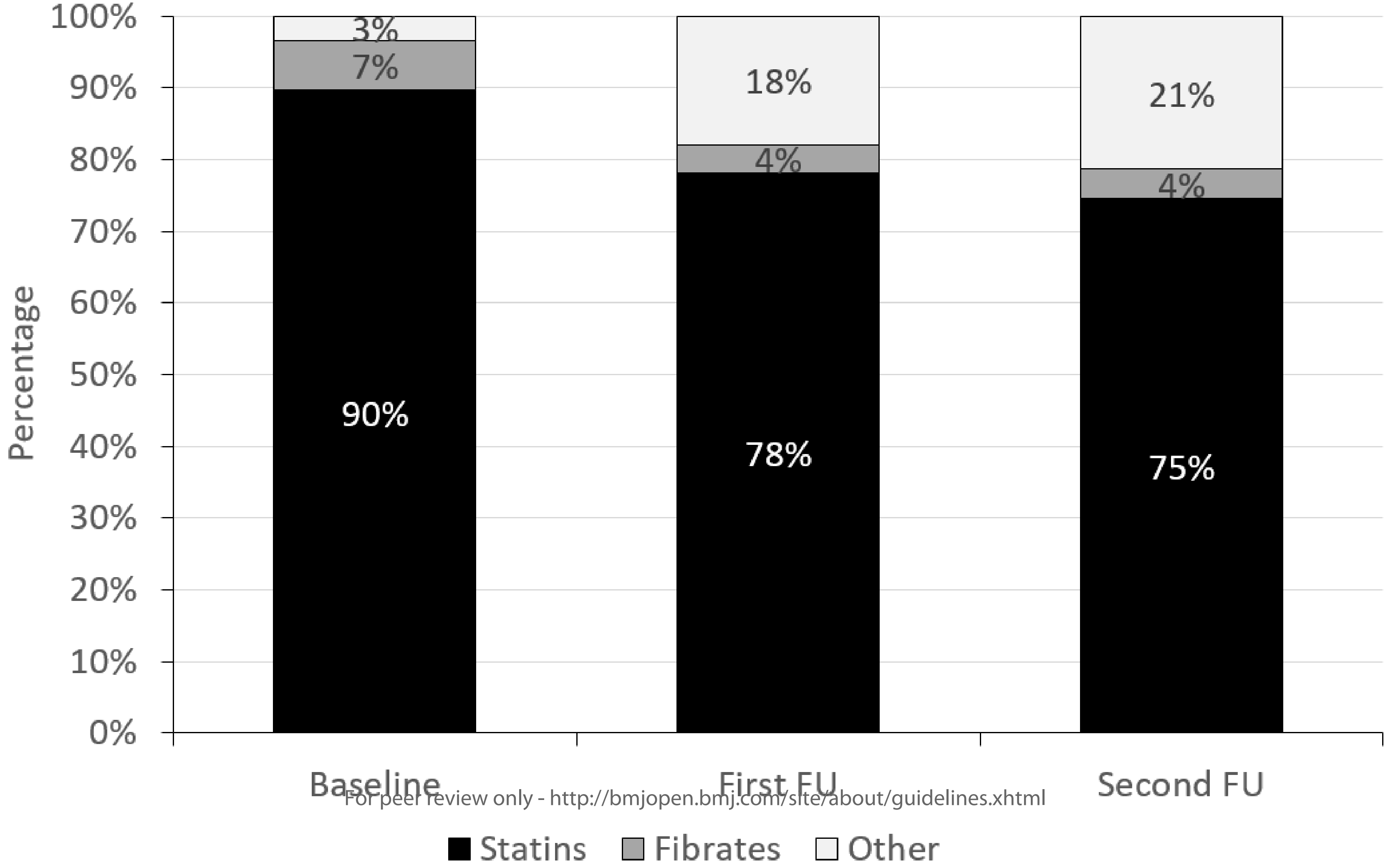

Supplemental figure 3: Generation and potency of statin drugs, first (2009-2012) and second (2014-2017) follow-ups of the CoLaus|PsyCoLaus study, Lausanne, Switzerland. Black: first generation or low potency; dark gray, second generation or intermediate potency; light gray, third generation or high potency.

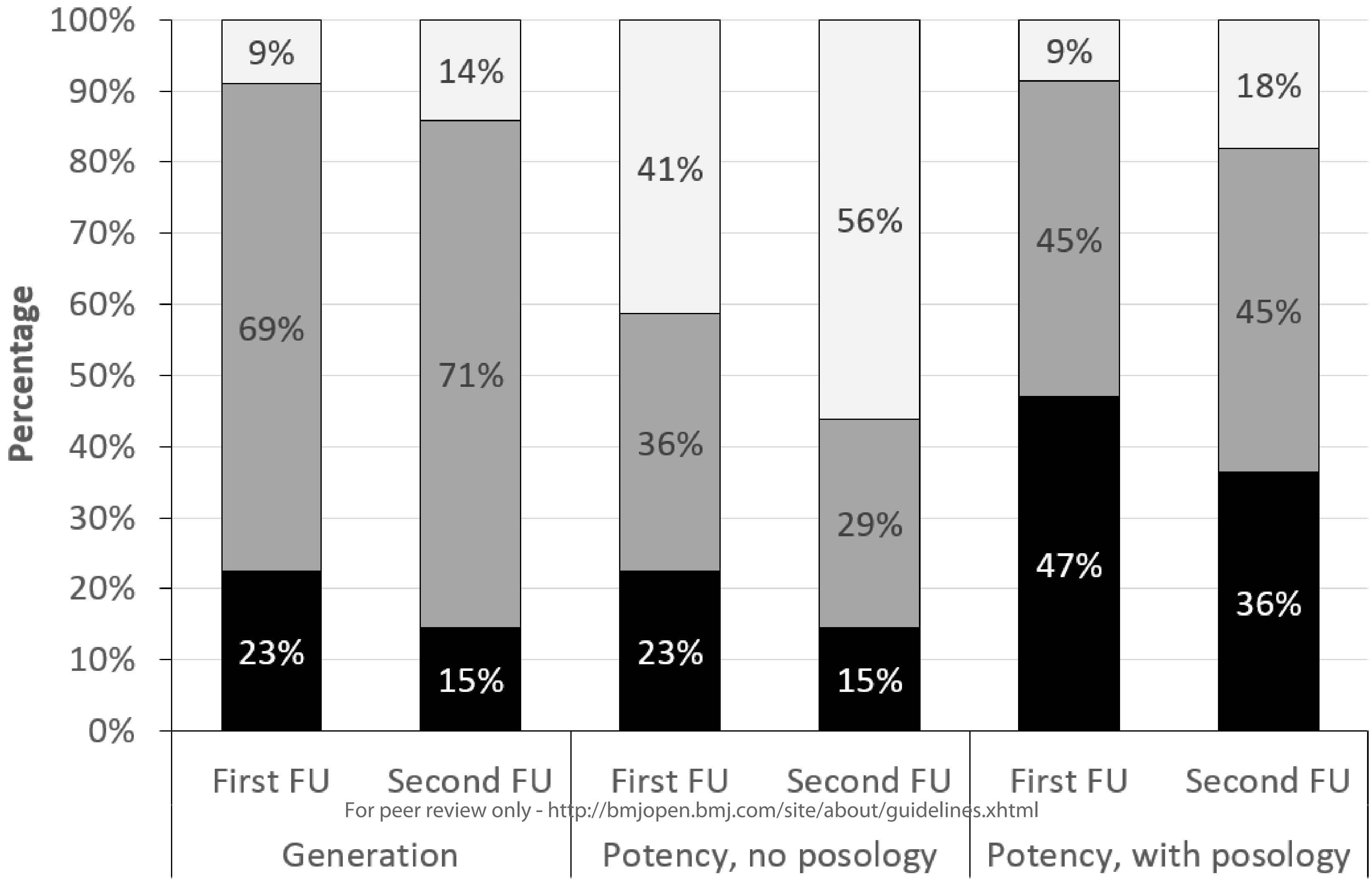

Supplemental figure 4: comparison of statin potency considering or not posology, first (2009-2012) and second (2014-2017) follow-ups of the CoLaus|PsyCoLaus study, Lausanne, Switzerland.

First follow-up

		With posology		
		Low	Intermediate	High
Without posology	Low	85	87	0
	Intermediate	283	7	0
	High	0	256	70

Second follow-up

		With posology		
		Low	Intermediate	High
Without posology	Low	67	43	0
	Intermediate	197	8	0
	High	0	282	136

Supplemental information

Supplemental table 1: statins classification regarding generation and potency.

Statin	ATC code	Generation	Potency, no posology	Potency, with posology
lovastatin	C10AA02	First	Low	20 low<br>40 intermediate
pravastatin	C10AA03	First	Low	<40 low<br>40-80 intermediate
fluvastatin	C10AA04	First	Low	10 low<br>40-80 intermediate
simvastatin	C10AA01	Second	Intermediate	20-40 intermediate<br>80 high
pitavastatin	C10AA08	Second	Intermediate	2 intermediate<br>4 high
atorvastatin	C10AA05	Second	High	10-20 intermediate<br>40-80 high
cerivastatin	C10AA06	Third	High	100 intermediate<br>300 high
rosuvastatin	C10AA07	Third	High	5-10 intermediate<br>20 high

Statins were ordered according to their potency. Adapted from [1, 2].

For peer review only

Supplemental table 2: LDL-cholesterol thresholds used to define control of dyslipidemia according to the European Society of Cardiology / European Atherosclerosis Society (ESC/EAS) criteria.

Risk categories according to SCORE	Control
Very high risk	
10–y risk >10%, diabetes or personal history of CVD	<1.8 mmol/L (70 mg/dL)
High risk	
10–y risk 5% – 10%	<2.6 mmol/L (100 mg/dL)
Other categories	
Less than 2 CVD risk factors and 10–y risk <5%	<4.0 mmol/L (155 mg/dL)

ESC/EAS criteria as stated in [3]. Risk categories based on the SCORE equation recalibrated for the Swiss population [4].

For peer review only

Supplemental table 3: LDL-cholesterol thresholds used to define control of dyslipidemia according to the Swiss Group for Lipids and Atherosclerosis.

**Risk categories ***	**Control**
High–risk	
10–y risk >20%, diabetes or personal history of CVD	<2.6 mmol/L (100 mg/dL)
Intermediate risk	
At least two CVD risk factors and 10–y risk 10% – 20%	<3.4 mmol/L (130 mg/dL)
Low risk	
At least two CVD risk factors and 10–y risk <10%	<4.2 mmol/L (160 mg/dL)
Very low risk	
Less than 2 CVD risk factors and 10–y risk <10%	<4.2 mmol/L (160 mg/dL)

* Risk calculation as defined in https://www.agla.ch/fr/calculateurs-outils/calculateur-de-risque-du-gsla. According to [5].

For peer review only

Supplemental table 4: list of specific SNPs interacting with the efficacy of statin treatment.

Gene	SNP	Simvastatin	Pravastatin	Atorvastatin	Rosuvastatin	Lovastatin	Fluvastatin
ABCB1	rs1045642	X, ↑		O			
ABCB1	rs1128503	↑					
ABCB1	rs1922242	O MI	O MI low	O MI low			O
ABCB1	rs2032582	↑					
ABCB1	rs3789244	O MI	O MI	O MI			
ABCG2/BCRP	rs2199936				O		
ABCG2/BCRP	rs2231137				X		
ABCG2/BCRP	rs2231142			O	X		X
ABCG8	rs11887534			O			
ACE	rs1799752						↑
APOA1	rs670		O	O			
APOA1	rs5069			O			
APOA5	rs662799	O		O		O	
APOC1	rs4420638	O	O	O	O		
APOE	rs429358	O		↑		↓	
APOE	rs445925			X			
APOE	rs449647			↑			
APOE	rs71352238				O		
APOE	rs7412		↑	↑		↓	
CETP	rs5783961						O
CETP	rs5882	O					
CETP	rs708272	O	O	O			
CLMN	rs8014194	O	O	O			
CYP2C9	rs1057910						X
CYP2D6	rs3892097			X			
CYP3A4	rs2740574			↑			
CYP3A4	rs355599367	X, ↑					
CYP3A4	rs55951658	O					
CYP3A5	rs776746	↓		X, ↓		↓	
CYP7A1	rs3808607			↓			
ESR1	rs2234693			O			
ESR1	rs9340799			-			
HMGCR	rs12916			O			
HMGCR	rs17238540		-				
HMGCR	rs17244841		↓				
HMGCR	rs17671591			X			
HMGCR	rs5908			-			
KIF6	rs20455		O	O			
LDLR	rs5925						↑
LPA	rs10455872			-	O		
LPL	rs328				O		
PCSK9	rs17111584				O		
PON1	rs662		O				
SCAP	rs12487736	↑					
SCCO1B1	rs2306283		↓		X		↑
SLCO1B1	rs11045819						↑
SLCO1B1	rs4149015		X				
SLCO1B1	rs4149056	X, ↓	X, ↓	X, ↓	X		
SOD2	rs4880				↑		
SREBF2	rs4822063			↓			
TLR4	rs4986790		O				

↑, increases effect; ↓, decreases effect; X, safety issues; O, other; MI, myocardial infarction. Data adapted from [6].

For peer review only

For peer review only - http://bmjopen.bmj.com/site/about/guidelines.xhtml

Supplemental table 5: data used to compute the genetic risk scores for total, LDL and HDL-cholesterol levels.

Score / Locus	Chr	SNP	Alleles (+/-)	Minor allele	β for weighting
**Total cholesterol**					
ASAP3	1	rs1077514	T/C	C	-0.030
LDLRAP1	1	rs12027135	T/A	A	-1.22
PCSK9	1	rs2479409	G/A	G	1.96
MOSC1	1	rs2807834	G/T	T	-1.38
ANGPTL3	1	rs3850634	T/G	G	-2.60
IRF2BP2	1	rs514230	T/A	A	-1.36
SORT1	1	rs629301	T/G	G	-5.41
EVI5	1	rs7515577	A/C	C	-1.18
INSIG2	2	rs10490626	G/A	A	0.042
UGT1A1	2	rs11563251	T/C	T	0.037
FAM117B	2	rs11694172	G/A	G	0.028
GCKR	2	rs1260326	T/C	T	1.91
APOB	2	rs1367117	A/G	A	4.16
LOC8431	2	rs2030746	T/C	T	0.020
ABCB11	2	rs2287623	G/A	G	0.027
ABCG5/8	2	rs4299376	G/T	G	3.01
RAB3GAP1	2	rs6759321	T/G	T	1.18
PXK	3	rs13315871	G/A	A	-0.036
RAF1	3	rs2290159	G/C	C	-1.42
CMTM6	3	rs7640978	C/T	T	-0.038
LRPAP1	4	rs6831256	G/A	G	-0.022
HMGCR	5	rs12916	C/T	C	2.84
CSNK1G3	5	rs4530754	A/G	G	-0.023
TIMD4	5	rs6882076	C/T	T	-1.98
LPA	6	rs1564348	C/T	C	2.18
HFE	6	rs1800562	G/A	A	-2.16
KCNK17	6	rs2758886	A/G	A	0.023
MYLIP	6	rs3757354	C/T	T	-1.46
HBS1L	6	rs9376090	C/T	T	-0.025
FRK	6	rs9488822	A/T	T	-1.18
GPR146	7	rs1997243	G/A	G	0.033
DNAH11	7	rs2285942	T/C	T	1.70
SOX17	8	rs10102164	A/G	A	0.030
CYP741	8	rs1030431	A/G	A	1.26
PLEC1	8	rs11136341	G/A	G	1.34
NAT2	8	rs1961456	G/A	G	1.07
PPP1R3B	8	rs2126259	C/T	T	-3.14
TRPS1	8	rs2737229	A/C	C	-1.11
TRIB1	8	rs2954022	C/A	A	-2.30
ABCA1	9	rs1883025	C/T	T	-2.24
VLDLR	9	rs3780181	A/G	G	-0.044
TTC39B	9	rs581080	C/G	G	-1.57
ABO	9	rs651007	T/C	T	2.30
VIM-CUBN	10	rs10904908	G/A	G	0.025
GPAM	10	rs2255141	A/G	A	1.14
MARCH8-ALOX5	10	rs970548	C/A	C	-0.026

For peer review only

For peer review only - http://bmjopen.bmj.com/site/about/guidelines.xhtml

SPTY2D1	11	rs10832963	G/T	T	-1.06
ST3GAL4	11	rs11220463	T/A	T	2.01
PHLDB1	11	rs11603023	T/C	T	0.022
FADS1-2-3	11	rs174550	T/C	C	-1.78
UBASH3B	11	rs7941030	C/T	C	0.97
APOA1	11	rs964184	G/C	G	4.68
PHC1-A2ML1	12	rs4883201	A/G	G	-0.035
LIPC	15	rs1532085	A/G	A	1.54
HPR	16	rs2000999	A/G	A	2.34
CETP	16	rs3764261	A/C	A	1.67
DLG4	17	rs314253	T/C	C	-0.023
OSBPL7	17	rs7206971	A/G	A	1.01
LIPG	18	rs7239867	G/A	A	-1.94
CILP2	19	rs10401969	T/C	C	-4.74
FLJ36070	19	rs492602	G/A	G	1.27
LDLR	19	rs6511720	G/T	T	-7.09
HNF4A	20	rs1800961	C/T	T	-4.73
ERGIC3	20	rs2277862	C/T	T	-1.19
MAFB	20	rs2902940	A/G	G	-1.38
TOP1	20	rs4297946	C/G	C	1.52
TOM1	22	rs138777	A/G	A	0.021
PPARA	22	rs4253772	T/C	T	0.032
**LDL cholesterol**					
LDLRAP1	1	rs12027135	T/A	A	-1.10
PIGV-NR0B2	1	rs12748152	T/C	T	0.05
PCSK9	1	rs2479409	G/A	G	2.01
ANXA9-CERS2	1	rs267733	A/G	G	-0.03
MOSC1	1	rs2807834	G/T	T	-1.09
ANGPTL3	1	rs3850634	T/G	G	-1.59
IRF2BP2	1	rs514230	T/A	A	-1.13
SORT1	1	rs629301	T/G	G	-5.65
INSIG2	2	rs10490626	G/A	A	-0.05
UGT1A1	2	rs11563251	T/C	T	0.03
FN1	2	rs1250229	C/T	T	-0.02
APOB	2	rs1367117	A/G	A	4.05
LOC84931	2	rs2030746	T/C	T	0.02
EHBP1	2	rs2710642	A/G	G	-0.02
ABCG5/8	2	rs4299376	G/T	G	2.75
ACAD11	3	rs17404153	G/T	T	-0.03
CMTM6	3	rs7640978	C/T	T	-0.04
LRPAP1	4	rs6831256	G/A	G	0.02
HMGCR	5	rs12916	C/T	C	2.45
CSNK1G3	5	rs4530754	A/G	G	-0.03
TIMD4	5	rs6882076	C/T	T	-1.67
FRK	6	rs11153594	C/T	T	-0.89
LPA	6	rs1564348	C/T	C	1.95
HFE	6	rs1800562	G/A	A	-2.22
MYLIP	6	rs3757354	C/T	T	-1.43
DNAH11	7	rs12670798	C/T	C	1.26
NPC1L1	7	rs217386	G/A	A	-1.17
SOX17	8	rs10102164	A/G	A	0.03
CYP741	8	rs1030431	A/G	A	0.95

For peer review only

PLEC1	8	rs11136341	G/A	G	1.40
PPP1R3B	8	rs2126259	C/T	T	-3.14
TRIB1	8	rs2954022	C/A	A	-1.84
VLDLR	9	rs3780181	A/G	G	-0.04
ABO	9	rs649129	T/C	T	2.05
GPAM	10	rs1129555	A/G	A	1.08
ST3GAL4	11	rs11220462	A/G	A	1.95
FADS1-2-3	11	rs174583	C/T	T	-1.71
APOA1	11	rs964184	G/C	G	2.85
BRCA2	13	rs4942486	T/C	T	0.02
HPR	16	rs2000999	A/G	A	2.00
CETP	16	rs247616	C/T	T	-1.45
DLG4	17	rs314253	T/C	C	-0.02
OSBPL7	17	rs7206971	A/G	A	1.01
CILP2	19	rs10401969	T/C	C	-3.11
SNX5	20	rs2328223	C/A	C	0.03
MAFB	20	rs2902941	A/G	G	-0.98
SPTLC3	20	rs364585	G/A	A	-0.03
TOP1	20	rs909802	T/C	T	1.41
PPARA	22	rs4253772	T/C	T	0.03
MTMR3	22	rs5763662	T/C	T	0.08
**HDL cholesterol**					
HDGF-PMVK	1	rs12145743	G/T	G	0.200
PIGV-NR0B2	1	rs12748152	C/T	T	-0.051
ZNF648	1	rs1689800	G/A	G	-0.47
ANGPTL1	1	rs4650994	G/A	G	0.021
PABPC4	1	rs4660293	G/A	G	-0.48
GALNT2	1	rs4846914	G/A	G	-0.61
APOB	2	rs1042034	T/C	C	0.90
COBLL1	2	rs12328675	T/C	C	0.68
IRS1	2	rs1515100	A/C	C	0.46
STAB1	3	rs13326165	A/G	A	0.029
ACAD11	3	rs17404153	G/T	T	0.028
RBM5	3	rs2013208	T/C	T	0.025
ATG7	3	rs2606736	C/T	C	0.025
GSK3B	3	rs6805251	T/C	T	0.020
C4orf52	4	rs10019888	A/G	G	-0.027
SLC39A8	4	rs13107325	T/C	T	-0.84
ADH5	4	rs2602836	A/G	A	0.019
FAM13A	4	rs3822072	G/A	A	-0.025
ARL15	5	rs6450176	A/G	A	-0.49
RSPO3	6	rs1936800	C/T	C	0.020
C6orf106	6	rs2814944	A/G	A	-0.49
CITED2	6	rs605066	C/T	C	-0.39
VEGFA	6	rs998584	C/A	A	-0.026
MLXIPL	7	rs17145738	C/T	T	0.57
TMEM176A	7	rs17173637	T/C	C	-0.036
SNX13	7	rs4142995	G/T	T	-0.026
KLF14	7	rs4731702	C/T	T	0.59
IKZF1	7	rs4917014	G/T	G	0.022
DAGLB	7	rs702485	G/A	G	0.024
TRIB1	8	rs10808546	C/T	T	0.61

For peer review only

For peer review only - http://bmjopen.bmj.com/site/about/guidelines.xhtml

LPL	8	rs12678919	A/G	G	2.25
TRPS1	8	rs2293889	T/G	T	-0.44
PPP1R3B	8	rs9987289	A/G	A	-1.21
ABCA1	9	rs1883025	T/C	T	-0.94
TTC39B	9	rs643531	C/A	C	-0.72
MARCH8-ALOX5	10	rs970548	C/A	C	0.026
OR4C46	11	rs11246602	C/T	C	0.034
KAT5	11	rs12801636	A/G	A	0.024
FADS1-2-3	11	rs174601	T/C	T	-0.73
AMPD3	11	rs2923084	G/A	G	-0.41
LRP4	11	rs3136441	T/C	C	0.78
MOGAT2-DGAT2	11	rs499974	C/A	A	-0.026
UBASH3B	11	rs7115089	C/G	G	0.31
APOA1	11	rs964184	G/C	G	-1.50
LRP1	12	rs3741414	C/T	T	0.46
SBNO1	12	rs4759375	C/T	T	0.86
ZNF664	12	rs4765127	G/T	T	0.44
PDE3A	12	rs7134375	C/A	A	0.40
MVK	12	rs7134594	C/T	C	-0.44
SCARB1	12	rs838880	T/C	C	0.61
LIPC	15	rs1532085	G/A	A	1.45
LACTB	15	rs2652834	A/G	A	-0.39
FTO	16	rs1121980	G/A	A	-0.020
LCAT	16	rs16942887	G/A	A	1.27
CMIP	16	rs2925979	T/C	T	-0.45
CETP	16	rs3764261	C/A	A	3.39
PGS1	17	rs4082919	G/T	G	-0.40
ABCA8	17	rs4148008	G/C	G	-0.42
STARD3	17	rs881844	C/G	C	-0.51
MC4R	18	rs12967135	A/G	A	-0.42
LIPG	18	rs7241918	G/T	G	-1.31
HAS1	19	rs17695224	G/A	A	-0.029
LILRA3	19	rs386000	G/C	C	0.83
ANGPTL4	19	rs7255436	C/A	C	-0.45
PEPD	19	rs731839	A/G	G	-0.022
LOC55908	19	rs737337	C/T	C	-0.64
HNF4A	20	rs1800961	T/C	T	-1.88
PLTP	20	rs6065906	C/T	C	-0.93
UBE2L3	22	rs181362	T/C	T	-0.46

Chr, chromosome; SNP, single nucleotide polymorphism. Adapted from [7].

For peer review only

Supplemental table 6: bivariate comparisons of socio-economic and clinical characteristics between retained and excluded participants. Data from the baseline (2003-2006), first (2009-2012) and second (2014-2017) follow-ups of the CoLaus|PsyCoLaus study, Lausanne, Switzerland.

	Baseline			First follow-up			Second follow-up		
	**Included**	**Excluded**	**p-value**	**Included**	**Excluded**	**p-value**	**Included**	**Excluded**	**p-value**
N	**617**	**92**		**844**	**212**		**798**	**353**	
Age (years)	61.6 ± 8.5	58.1 ± 9.6	<0.001	64.5 ± 8.8	61.7 ± 10.4	<0.001	68.1 ± 9.2	67.5 ± 10.3	0.309
Women (%)	263 (42.6)	28 (30.4)	0.027	409 (48.5)	90 (42.5)	0.117	401 (50.3)	166 (47.0)	0.313
Swiss national (%)	430 (69.7)	59 (64.1)	0.282	600 (71.1)	116 (54.7)	<0.001	563 (70.6)	191 (54.1)	<0.001
Education (%)			0.301			0.027			0.714
High	66 (10.7)	5 (5.5)		123 (14.6)	47 (22.2)		120 (15.0)	54 (15.3)	
Middle	132 (21.4)	20 (22.0)		185 (21.9)	43 (20.3)		180 (22.6)	72 (20.4)	
Low	419 (67.9)	66 (72.5)		536 (63.5)	122 (57.6)		498 (62.4)	227 (64.3)	
Married/couple (%)	428 (69.4)	65 (71.4)	0.690	505 (59.8)	121 (57.1)	0.465	448 (56.1)	166 (53.9)	0.501
BMI (kg/m^2)	28.0 ± 4.5	28.1 ± 4.8	0.792	27.6 ± 4.8	28.0 ± 5.0	0.398			
BMI categories (%)			0.979			0.986			0.197
Normal	155 (25.1)	24 (26.1)		243 (28.8)	53 (28.2)		254 (31.8)	59 (26.6)	
Overweight	277 (44.9)	41 (44.6)		378 (44.8)	85 (45.2)		327 (41.0)	91 (41.0)	
Obese	185 (30.0)	27 (29.4)		223 (26.4)	50 (26.6)		217 (27.2)	72 (32.4)	
Smoking (%)			0.064			0.099			0.067
Never	210 (34.0)	39 (42.9)		295 (35.0)	73 (34.8)		290 (36.3)	103 (38.7)	
Former	255 (41.3)	26 (28.6)		385 (45.6)	83 (39.5)		365 (45.7)	102 (38.4)	
Current	152 (24.6)	26 (28.6)		164 (19.4)	54 (25.7)		143 (17.9)	61 (22.9)	
Alcohol drinker (%)	456 (73.9)	65 (70.7)	0.510	632 (74.9)	147 (69.3)	0.101	525 (72.3)	149 (62.3)	0.004
Treatment for									
Hypertension	303 (49.1)	49 (53.3)	0.457	436 (51.7)	101 (47.6)	0.296	425 (53.3)	190 (53.8)	0.859
Diabetes	95 (15.4)	24 (26.1)	0.010	148 (17.5)	40 (18.9)	0.650	157 (19.7)	69 (19.6)	0.960
Parental history (%)	164 (26.6)	24 (26.1)	0.920	218 (25.8)	52 (24.5)	0.698	211 (26.4)	64 (18.1)	0.002
CVD risk (%)			0.551			0.717			<0.001

For peer review only - http://bmjopen.bmj.com/site/about/guidelines.xhtml

Low	267 (43.3)	36 (39.1)	367 (43.5)	89 (42.0)	356 (44.6)	103 (29.2)
Intermediate	182 (29.5)	26 (28.3)	203 (24.1)	48 (22.6)	165 (20.7)	121 (34.3)
High	168 (27.2)	30 (32.6)	274 (32.5)	75 (35.4)	277 (34.7)	129 (36.5)

BMI, body mass index; CVD, cardio vascular disease. Results are expressed as number of participants (column %) for categorical variables and as average ± standard deviation for continuous variables. Between-groups comparisons performed using chi-square for categorical variables and student's t-test for continuous variables.

For peer review only

Supplemental table 7: bivariate comparison of the statin generation and potency among participants treated for dyslipidemia, according to controlled and uncontrolled status as per European Society of Cardiology / European Atherosclerosis Society (ESC/EAS) criteria. Data from the first (2009-2012) and second (2014-2017) follow-ups of the CoLaus|PsyCoLaus study, Lausanne, Switzerland. Analysis restricted to participants taking statins.

	**First follow-up**			**Second follow-up**		
	**Uncontrolled**	**Controlled**	**p-value**	**Uncontrolled**	**Controlled**	**p-value**
N	**373**	**288**		**428**	**370**	
Generation			0.004			0.212
First	99 (26.5)	50 (17.4)		54 (16.5)	32 (12.2)	
Second	249 (66.8)	204 (70.8)		233 (71.0)	188 (71.8)	
Third	25 (6.7)	34 (11.8)		41 (12.5)	42 (16.0)	
Potency §			0.018			0.018
Low	99 (26.5)	50 (17.4)		54 (16.5)	32 (12.2)	
Intermediate	130 (34.9)	109 (37.9)		107 (32.6)	66 (25.2)	
High	144 (38.6)	129 (44.8)		167 (50.9)	164 (62.6)	
N	**348**	**274**		**328**	**262**	
Potency †			0.911			0.067
Low	166 (47.7)	126 (46.0)		117 (40.6)	72 (31.2)	
Intermediate	153 (44.0)	124 (45.3)		125 (43.4)	111 (48.0)	
High	29 (8.3)	24 (8.7)		46 (16.0)	48 (20.8)	

§, not considering posology; †, considering posology. Results are expressed as number of participants (column %). Between-groups comparisons performed using chi-square.

For peer review only - http://bmjopen.bmj.com/site/about/guidelines.xhtml

Supplemental table 8: multivariable analysis of the factors associated with dyslipidemia control as per European Society of Cardiology / European Atherosclerosis Society criteria. Data from the first (2009-2012) and second (2014-2017) follow-ups of the CoLaus|PsyCoLaus study, Lausanne, Switzerland. Analysis taking into account statin potency, but not considering posology.

	First follow-up		Second follow-up	
	OR (95% CI)	p-value	OR (95% CI)	p-value
Age (per 10 years increase)	0.31 (0.23 - 0.41)	<0.001	0.36 (0.27 - 0.47)	<0.001
Man vs. woman	0.59 (0.38 - 0.90)	0.014	1.36 (0.88 - 2.11)	0.171
Swiss vs. Non-Swiss	1.16 (0.76 - 1.78)	0.495	1.17 (0.75 - 1.81)	0.487
Education				
High	1 (ref.)		1 (ref.)	
Middle	0.76 (0.39 - 1.48)	0.415	1.04 (0.52 - 2.08)	0.908
Low	0.87 (0.48 - 1.57)	0.649	1.02 (0.56 - 1.87)	0.937
p-value for trend	0.649		0.937	
Married vs. not married	1.23 (0.83 - 1.84)	0.299	1.20 (0.80 - 1.79)	0.386
Body mass index categories				
Normal	1 (ref.)		1 (ref.)	
Overweight	0.86 (0.53 - 1.38)	0.523	0.57 (0.35 - 0.93)	0.025
Obese	0.93 (0.54 - 1.61)	0.807	0.53 (0.30 - 0.94)	0.031
p-value for trend	0.807		0.031	
Smoking categories				
Never	1 (ref.)		1 (ref.)	
Former	1.13 (0.72 - 1.77)	0.590	1.19 (0.77 - 1.85)	0.428
Current	0.86 (0.48 - 1.55)	0.614	0.90 (0.49 - 1.65)	0.729
p-value for trend	0.614		0.729	
Alcohol drinker (yes vs. no)	0.81 (0.51 - 1.27)	0.360	0.68 (0.43 - 1.08)	0.101
AntiHTA ttt (yes vs. no)	0.95 (0.62 - 1.47)	0.821	0.80 (0.51 - 1.25)	0.327
Parental history (yes vs. no)	1.25 (0.79 - 1.99)	0.338	0.75 (0.47 - 1.21)	0.240
CVD risk				
Other	1 (ref.)		1 (ref.)	
High	0.63 (0.39 - 1.02)	0.060	1.37 (0.78 - 2.40)	0.275
Very high	0.08 (0.05 - 0.14)	<0.001	0.35 (0.21 - 0.58)	<0.001
p-value for trend	<0.001		<0.001	
LDL genetic risk score quartiles				
First	1 (ref.)		1 (ref.)	
Second	0.89 (0.52 - 1.53)	0.680	1.63 (0.92 - 2.86)	0.092
Third	0.92 (0.54 - 1.57)	0.757	1.76 (1.02 - 3.03)	0.041
Fourth	1.11 (0.65 - 1.91)	0.702	1.58 (0.90 - 2.77)	0.112
p-value for trend	0.694		0.109	
Number of drugs (per 1 unit)	1.14 (1.05 - 1.25)	0.002	1.06 (0.99 - 1.15)	0.099
Statin potency, no posology				
Low	1 (ref.)		1 (ref.)	
Intermediate	1.98 (1.18 - 3.34)	0.010	1.50 (0.80 - 2.82)	0.209

For peer review only - http://bmjopen.bmj.com/site/about/guidelines.xhtml
For peer review only

High	2.08 (1.25 - 3.46)	0.005	2.25 (1.26 - 4.01)	0.006
p-value for trend	0.005		0.006	
Fibrates	NC		2.16 (0.16 - 29.1)	0.560
Other hypolipidemic drugs	0.95 (0.41 - 2.22)	0.905	1.07 (0.48 - 2.37)	0.867

OR, odds ratio; CI, confidence interval; antiHTA ttt, antihypertensive drug treatment; NC, not computable. Results are expressed as odds ratio and (95% confidence interval). Statistical analysis using logistic regression.

For peer review only

Supplemental table 9: multivariable analysis of the factors associated with dyslipidemia control as per European Society of Cardiology / European Atherosclerosis Society criteria. Data from the first (2009-2012) and second (2014-2017) follow-ups of the CoLaus|PsyCoLaus study, Lausanne, Switzerland. Analysis taking into account statin potency and posology.

	**First follow-up**		**Second follow-up**	
	**OR (95% CI)**	**p-value**	**OR (95% CI)**	**p-value**
Age (per 10 years increase)	0.31 (0.24 - 0.42)	<0.001	0.37 (0.27 - 0.50)	<0.001
Man vs. woman	0.56 (0.36 - 0.86)	0.008	1.45 (0.91 - 2.31)	0.120
Swiss vs. Non-Swiss	1.14 (0.74 - 1.78)	0.548	1.11 (0.69 - 1.76)	0.672
Education				
High	1 (ref.)		1 (ref.)	
Middle	0.74 (0.37 - 1.48)	0.396	0.95 (0.46 - 1.95)	0.882
Low	0.86 (0.47 - 1.56)	0.619	0.94 (0.50 - 1.76)	0.835
p-value for trend	0.619		0.835	
Married vs. not married	1.25 (0.83 - 1.88)	0.29	1.17 (0.76 - 1.80)	0.481
Body mass index categories				
Normal	1 (ref.)		1 (ref.)	
Overweight	0.87 (0.54 - 1.42)	0.586	0.62 (0.37 - 1.05)	0.073
Obese	0.89 (0.50 - 1.56)	0.677	0.56 (0.31 - 1.02)	0.058
p-value for trend	0.677		0.058	
Smoking categories				
Never	1 (ref.)		1 (ref.)	
Former	1.12 (0.71 - 1.77)	0.629	1.12 (0.7 - 1.78)	0.636
Current	0.91 (0.50 - 1.65)	0.747	0.82 (0.42 - 1.58)	0.545
p-value for trend	0.747		0.545	
Alcohol drinker (yes vs. no)	0.73 (0.45 - 1.16)	0.183	0.58 (0.35 - 0.95)	0.031
AntiHTA ttt (yes vs. no)	0.99 (0.64 - 1.55)	0.977	0.89 (0.55 - 1.42)	0.618
Parental history (yes vs. no)	1.24 (0.77 - 1.98)	0.382	0.85 (0.51 - 1.41)	0.529
CVD risk				
Other	1 (ref.)		1 (ref.)	
High	0.64 (0.39 - 1.04)	0.074	1.29 (0.71 - 2.33)	0.400
Very high	0.08 (0.05 - 0.15)	<0.001	0.35 (0.20 - 0.61)	<0.001
p-value for trend	<0.001		<0.001	
LDL genetic risk score quartiles				
First	1 (ref.)		1 (ref.)	
Second	1.01 (0.58 - 1.75)	0.979	1.63 (0.89 - 2.96)	0.111
Third	1.15 (0.66 - 1.99)	0.623	1.89 (1.06 - 3.36)	0.031
Fourth	1.24 (0.71 - 2.16)	0.452	1.68 (0.93 - 3.02)	0.085
p-value for trend	0.392		0.073	
Number of drugs (per 1 unit)	1.13 (1.03 - 1.23)	0.008	1.04 (0.96 - 1.13)	0.366
Statin potency, with posology				
Low	1 (ref.)		1 (ref.)	
Intermediate	1.10 (0.73 - 1.65)	0.656	1.37 (0.86 - 2.17)	0.183

For peer review only - http://bmjopen.bmj.com/site/about/guidelines.xhtml

High	1.35 (0.63 - 2.88)	0.437	1.69 (0.89 - 3.21)	0.110
p-value for trend	0.437		0.110	
Fibrates	NC		2.20 (0.16 - 31.2)	0.559
Other hypolipidemic drugs	0.91 (0.38 - 2.21)	0.839	1.06 (0.45 - 2.47)	0.898

OR, odds ratio; CI, confidence interval; antiHTA ttt, antihypertensive drug treatment; NC, not computable. Results are expressed as odds ratio and (95% confidence interval). Statistical analysis using logistic regression.

For peer review only

For peer review only - http://bmjopen.bmj.com/site/about/guidelines.xhtml

Supplemental table 10: bivariate comparisons of socio-economic and clinical characteristics among participants treated for dyslipidemia, according to controlled and uncontrolled status as per the Swiss Group for Lipids and Atherosclerosis criteria. Data from the baseline (2003-2006), first (2009-2012) and second (2014-2017) follow-ups of the CoLaus|PsyCoLaus study, Lausanne, Switzerland.

	**Baseline**			**First follow-up**			**Second follow-up**		
	**Uncontrolled**	**Controlled**	**p-value**	**Uncontrolled**	**Controlled**	**p-value**	**Uncontrolled**	**Controlled**	**p-value**
N	**184**	**433**		**266**	**578**		**139**	**659**	
Age (years)	61.5 ± 8.5	61.7 ± 8.5	0.728	64.5 ± 8.6	64.5 ± 8.9	0.947	67.1 ± 9.0	68.4 ± 9.3	0.139
Women (%)	81 (44.0)	182 (42.0)	0.648	119 (44.7)	290 (50.2)	0.142	72 (51.8)	329 (49.9)	0.688
Swiss national (%)	128 (70.0)	302 (69.6)	0.929	183 (69.1)	417 (72.0)	0.378	105 (74.5)	458 (69.7)	0.261
Education (%)			0.474			0.707			0.354
High	47 (10.9)	66 (10.7)		16 (6.0)	38 (6.6)		10 (7.2)	50 (7.6)	
Middle	98 (22.6)	132 (21.4)		39 (14.7)	89 (15.4)		17 (12.2)	53 (8.0)	
Low	288 (66.5)	419 (67.9)		50 (18.8)	125 (21.6)		23 (16.6)	95 (14.4)	
Married/couple (%)	127 (69.0)	301 (69.5)	0.903	353 (61.1)	505 (59.8)	0.279	74 (53.2)	374 (56.8)	0.448
BMI (kg/m^2)	28.4 ± 4.4	27.8 ± 4.5	0.150	28.2 ± 4.9	27.4 ± 4.7	0.038	28.6 ± 4.8	27.4 ± 4.9	0.008
BMI categories (%)			0.354			0.125			0.033
Normal	40 (21.7)	115 (26.6)		69 (25.9)	174 (30.1)		33 (23.7)	221 (33.5)	
Overweight	83 (45.1)	194 (44.8)		115 (43.2)	263 (45.5)		58 (41.7)	269 (40.8)	
Obese	61 (33.2)	124 (28.6)		82 (30.8)	141 (24.4)		48 (34.5)	169 (25.6)	
Smoking (%)			0.285			0.019			0.110
Never	152 (35.1)	210 (34.0)		218 (37.7)	295 (35.0)		235 (35.7)	290 (36.3)	
Former	182 (42.0)	255 (41.3)		259 (44.8)	385 (45.6)		312 (47.3)	365 (45.7)	
Current	99 (22.9)	152 (24.6)		101 (17.5)	164 (19.4)		112 (17.0)	143 (17.9)	
Alcohol drinker (%)	148 (80.4)	308 (71.1)	0.016	207 (77.8)	425 (73.5)	0.182	93 (74.4)	432 (71.9)	0.567
Treatment for									
Hypertension	98 (53.3)	205 (47.3)	0.179	41 (44.1)	395 (52.6)	0.121	79 (56.8)	346 (52.5)	0.352
Diabetes	36 (19.6)	59 (13.6)	0.061	13 (14.0)	135 (18.0)	0.339	34 (24.5)	123 (18.7)	0.118
Parental history (%)	134 (72.8)	319 (73.7)	0.828	69 (25.9)	149 (25.8)	0.960	94 (67.6)	493 (74.8)	0.081

For peer review only - http://bmjopen.bmj.com/site/about/guidelines.xhtml

CVD risk (%)			<0.001			<0.001			<0.001
Low	32 (17.4)	234 (54.0)		47 (17.7)	319 (55.2)		28 (20.1)	329 (49.9)	
Intermediate	70 (38.0)	114 (26.3)		74 (27.8)	130 (22.5)		36 (25.9)	131 (19.9)	
High	82 (44.6)	85 (19.6)		145 (54.5)	129 (22.3)		75 (54.0)	199 (30.2)	
Statins (%)	161 (87.5)	417 (96.3)	<0.001	200 (75.2)	461 (79.8)	0.134	88 (63.3)	504 (76.5)	0.001
Fibrates (%)	28 (15.2)	17 (3.9)	<0.001	17 (6.4)	16 (2.8)	0.012	7 (5.0)	25 (3.8)	0.497
Other (%)	7 (3.8)	15 (3.5)	0.835	55 (20.7)	96 (16.6)	0.152	46 (33.1)	123 (18.7)	<0.001
Number of drugs									
Including OTC	-	-		4 [3 - 6]	4 [2 - 6]	0.229 §	5 [3 - 7]	5 [3 - 7]	0.842 §
Excluding OTC	-	-		4 [2 - 6]	3 [2 - 5]	0.246 §	4 [2 - 6]	4 [2 - 6]	0.642 §
Genetic risk scores									
Total cholesterol	-2.7 ± 9.4	-3.7 ± 9.6	0.254	-3.7 ± 9.5	-3.5 ± 8.9	0.763	-3.3 ± 9.4	-3.8 ± 8.9	0.562
LDL-cholesterol	-2.4 ± 7.5	-2.9 ± 7.5	0.461	-2.5 ± 7.6	-2.4 ± 7.2	0.847	-2.5 ± 7.3	-2.9 ± 7.0	0.467
HDL-cholesterol	-6.5 ± 3.4	-6.7 ± 3.6	0.649	0.2 ± 7.4	-6.6 ± 3.5	0.312	-6.9 ± 3.8	-6.7 ± 3.6	0.539

BMI, body mass index; CVD, cardio vascular disease; OTC, over the counter. Results are expressed as number of participants (column %) for categorical variables and as average ± standard deviation or as median [interquartile range] for continuous variables. Between-groups comparisons performed using chi-square for categorical variables and student's t-test or Kruskal-Wallis nonparametric test (§) for continuous variables.

For peer review only

Supplemental table 11: multivariable analysis of the factors associated with dyslipidemia control as per the Swiss Group for Lipids and Atherosclerosis criteria. Data from the baseline (2003-2006), first (2009-2012) and second (2014-2017) follow-ups of the CoLaus|PsyCoLaus study, Lausanne, Switzerland.

	Baseline		First follow-up		Second follow-up	
	OR (95% CI)	p-value	OR (95% CI)	p-value	OR (95% CI)	p-value
Age (per 10 years increase)	1.17 (0.90 - 1.51)	0.231	1.05 (0.85 - 1.30)	0.627	1.18 (0.90 - 1.54)	0.221
Man vs. woman	1.70 (1.08 - 2.69)	0.022	1.15 (0.80 - 1.66)	0.458	1.59 (1.00 - 2.53)	0.050
Swiss vs. Non-Swiss	1.26 (0.81 - 1.96)	0.298	1.30 (0.90 - 1.86)	0.159	0.74 (0.45 - 1.21)	0.232
Education						
High	1 (ref.)		1 (ref.)		1 (ref.)	
Middle	1.23 (0.58 - 2.62)	0.586	1.12 (0.64 - 1.96)	0.690	0.44 (0.21 - 0.92)	0.030
Low	1.07 (0.55 - 2.05)	0.848	0.81 (0.49 - 1.32)	0.396	0.61 (0.30 - 1.22)	0.163
p-value for trend	0.848		0.396		0.163	
Married vs. not married	1.11 (0.71 - 1.72)	0.652	1.13 (0.81 - 1.59)	0.466	0.81 (0.52 - 1.26)	0.349
Body mass index categories						
Normal	1 (ref.)		1 (ref.)		1 (ref.)	
Overweight	0.75 (0.45 - 1.25)	0.268	0.92 (0.60 - 1.40)	0.691	0.57 (0.33 - 0.98)	0.043
Obese	1.05 (0.60 - 1.86)	0.854	0.96 (0.59 - 1.54)	0.854	0.58 (0.31 - 1.09)	0.091
p-value for trend	0.854		0.691		0.091	
Smoking categories						
Never	1 (ref.)		1 (ref.)		1 (ref.)	
Former	1.28 (0.78 - 2.08)	0.328	0.91 (0.62 - 1.35)	0.646	1.96 (1.19 - 3.23)	0.008
Current	1.29 (0.73 - 2.26)	0.380	0.91 (0.56 - 1.47)	0.692	1.76 (0.93 - 3.32)	0.081
p-value for trend	0.380		0.692		0.081	
Alcohol drinker (yes vs. no)	0.46 (0.28 - 0.77)	0.003	0.75 (0.50 - 1.12)	0.155	0.74 (0.45 - 1.23)	0.246
Antihypertensive treatment (yes vs. no)	1.50 (0.97 - 2.29)	0.065	1.48 (1.03 - 2.12)	0.036	1.04 (0.64 - 1.69)	0.862
Parental history (yes vs. no)	0.99 (0.61 - 1.59)	0.964	0.96 (0.65 - 1.42)	0.847	0.65 (0.40 - 1.06)	0.086
CVD risk (GSLA criterion)						
Low	1 (ref.)		1 (ref.)		1 (ref.)	

For peer review only

Intermediate	0.16 (0.09 - 0.28)	<0.001	0.19 (0.12 - 0.31)	<0.001	0.22 (0.11 - 0.42)	<0.001
High	0.08 (0.05 - 0.14)	<0.001	0.08 (0.05 - 0.13)	<0.001	0.100 (0.06 - 0.20)	<0.001
p-value for trend	<0.001		<0.001		<0.001	
LDL genetic risk score quartiles						
First	1 (ref.)		1 (ref.)		1 (ref.)	
Second	0.89 (0.50 - 1.58)	0.681	1.07 (0.67 - 1.69)	0.784	1.99 (1.06 - 3.74)	0.033
Third	0.54 (0.31 - 0.96)	0.034	1.11 (0.70 - 1.77)	0.645	0.93 (0.51 - 1.70)	0.815
Fourth	0.74 (0.42 - 1.30)	0.293	1.12 (0.70 - 1.77)	0.641	0.77 (0.43 - 1.38)	0.388
p-value for trend	0.125		0.617		0.108	
Hypolipidemic drug treatment						
Statins	0.35 (0.03 - 4.26)	0.411	1.19 (0.66 - 2.15)	0.570	2.21 (1.14 - 4.28)	0.019
Fibrates	0.06 (0.01 - 0.63)	0.019	0.29 (0.11 - 0.76)	0.012	0.61 (0.2 - 1.86)	0.389
Other	0.80 (0.28 - 2.27)	0.674	0.45 (0.25 - 0.81)	0.008	0.47 (0.25 - 0.89)	0.020

OR, odds ratio; CI, confidence interval. Results are expressed as odds ratio and (95% confidence interval). Statistical analysis using logistic regression.

For peer review only

For peer review only - http://bmjopen.bmj.com/site/about/guidelines.xhtml

Supplemental table 12: bivariate comparisons of the statin generation and potency among participants treated for dyslipidemia, according to controlled and uncontrolled status as per the Swiss Group for Lipids and Atherosclerosis criteria. Data from the first (2009-2012) and second (2014-2017) follow-ups of the CoLaus|PsyCoLaus study, Lausanne, Switzerland. Analysis restricted to participants taking statins.

	First follow-up			Second follow-up		
	**Uncontrolled**	**Controlled**	**p-value**	**Uncontrolled**	**Controlled**	**p-value**
N	**200**	**461**		**88**	**502**	
Generation			0.003			0.026
First	62 (31.0)	87 (18.9)		21 (23.9)	65 (13.0)	
Second	122 (61.0)	331 (71.8)		55 (62.5)	366 (72.9)	
Third	16 (8.0)	43 (9.3)		12 (13.6)	71 (14.1)	
Potency §			0.002			0.005
Low	62 (31.0)	87 (18.9)		21 (23.9)	65 (13.0)	
Intermediate	70 (35.0)	169 (36.7)		30 (34.1)	143 (28.5)	
High	68 (34.0)	205 (44.5)		37 (42.1)	294 (58.6)	
N	**186**	**436**		**77**	**442**	
Potency §			0.386			0.199
Low	94 (50.5)	198 (45.4)		34 (44.2)	155 (35.1)	
Intermediate	75 (40.3)	202 (46.3)		28 (36.4)	208 (47.1)	
High	17 (9.1)	36 (8.3)		15 (19.5)	79 (17.9)	

§, not considering posology; †, considering posology. Results are expressed as number of participants (column %). Between-groups comparisons performed using chi-square.

For peer review only

Supplemental table 13: multivariable analysis of the factors associated with dyslipidemia control as per the Swiss Group for Lipids and Atherosclerosis criteria. Data from the first (2009-2012) and second (2014-2017) follow-ups of the CoLaus|PsyCoLaus study, Lausanne, Switzerland. Analysis taking into account statin generation.

	**First follow-up**		**Second follow-up**	
	**OR (95% CI)**	**p-value**	**OR (95% CI)**	**p-value**
Age (per 10 years increase)	1.20 (0.93 - 1.56)	0.159	1.04 (0.73 - 1.48)	0.823
Man vs. woman	1.59 (1.03 - 2.45)	0.037	2.23 (1.23 - 4.02)	0.008
Swiss vs. Non-Swiss	1.25 (0.81 - 1.92)	0.315	0.80 (0.43 - 1.47)	0.468
Education				
High	1 (ref.)		1 (ref.)	
Middle	1.09 (0.54 - 2.19)	0.805	0.85 (0.32 - 2.22)	0.733
Low	0.83 (0.46 - 1.52)	0.551	1.13 (0.46 - 2.74)	0.791
p-value for trend	0.551		0.791	
Married vs. not married	1.24 (0.84 - 1.84)	0.287	0.97 (0.55 - 1.71)	0.917
Body mass index categories				
Normal	1 (ref.)		1 (ref.)	
Overweight	0.87 (0.53 - 1.45)	0.603	0.61 (0.29 - 1.28)	0.191
Obese	0.86 (0.49 - 1.50)	0.591	0.47 (0.21 - 1.06)	0.068
p-value for trend	0.591		0.068	
Smoking categories				
Never	1 (ref.)		1 (ref.)	
Former	0.91 (0.58 - 1.44)	0.688	1.79 (0.96 - 3.36)	0.068
Current	0.96 (0.54 - 1.72)	0.891	1.22 (0.56 - 2.66)	0.625
p-value for trend				
Alcohol drinker (yes vs. no)	0.67 (0.41 - 1.08)	0.099	0.95 (0.51 - 1.78)	0.882
AntiHTA ttt (yes vs. no)	1.19 (0.77 - 1.84)	0.438	1.11 (0.60 - 2.05)	0.733
Parental history (yes vs. no)	0.98 (0.61 - 1.58)	0.945	0.54 (0.28 - 1.03)	0.060
CVD risk (GSLA criterion)				
Low	1 (ref.)		1 (ref.)	
Intermediate	0.18 (0.10 - 0.32)	<0.001	0.19 (0.07 - 0.49)	0.001
High	0.05 (0.03 - 0.10)	<0.001	0.06 (0.02 - 0.14)	<0.001
p-value for trend	<0.001		<0.001	
LDL genetic risk score quartiles				
First	1 (ref.)		1 (ref.)	
Second	1.04 (0.61 - 1.76)	0.888	2.95 (1.29 - 6.72)	0.010
Third	1.21 (0.70 - 2.08)	0.492	1.06 (0.51 - 2.19)	0.881
Fourth	1.29 (0.75 - 2.20)	0.359	1.18 (0.57 - 2.47)	0.653
p-value for trend	0.298		0.666	
Number of drugs (per 1 unit)	1.14 (1.05 - 1.25)	0.003	1.16 (1.04 - 1.29)	0.008
Statins generation				
First	1 (ref.)		1 (ref.)	
Second	2.54 (1.61 - 4.01)	<0.001	4.17 (2.07 - 8.42)	<0.001
Third	2.59 (1.19 - 5.61)	0.016	3.44 (1.31 - 8.99)	0.012

For peer review only - http://bmjopen.bmj.com/site/about/guidelines.xhtml

p-value for trend	0.016		0.012	
Fibrates	NC		NC	
Other	0.62 (0.27 - 1.43)	0.263	1.09 (0.36 - 3.3)	0.880

OR, odds ratio; CI, confidence interval; antiHTA ttt, antihypertensive drug treatment; NC, not computable. Results are expressed as odds ratio and (95% confidence interval). Statistical analysis by logistic regression.

For peer review only

For peer review only - http://bmjopen.bmj.com/site/about/guidelines.xhtml

Supplemental table 14: multivariable analysis of the factors associated with dyslipidemia control as per the Swiss Group for Lipids and Atherosclerosis criteria. Data from the first (2009-2012) and second (2014-2017) follow-ups of the CoLaus|PsyCoLaus study, Lausanne, Switzerland. Analysis taking into account statin potency, but not considering posology.

	First follow-up		Second follow-up	
	**OR (95% CI)**	**p-value**	**OR (95% CI)**	**p-value**
Age (per 10 years increase)	1.22 (0.94 - 1.59)	0.127	1.06 (0.74 - 1.51)	0.748
Man vs. woman	1.57 (1.02 - 2.43)	0.040	2.13 (1.17 - 3.87)	0.013
Swiss vs. Non-Swiss	1.24 (0.81 - 1.92)	0.322	0.82 (0.45 - 1.52)	0.533
Education				
High	1 (ref.)		1 (ref.)	
Middle	1.07 (0.53 - 2.14)	0.856	0.77 (0.29 - 2.04)	0.601
Low	0.83 (0.45 - 1.50)	0.531	1.05 (0.43 - 2.58)	0.914
p-value for trend	0.531		0.914	
Married vs. not married	1.26 (0.85 - 1.86)	0.259	1.00 (0.57 - 1.76)	0.995
Body mass index categories				
Normal	1 (ref.)		1 (ref.)	
Overweight	0.89 (0.54 - 1.47)	0.637	0.64 (0.31 - 1.35)	0.245
Obese	0.85 (0.49 - 1.49)	0.570	0.49 (0.22 - 1.10)	0.085
p-value for trend	0.570		0.085	
Smoking categories				
Never	1 (ref.)		1 (ref.)	
Former	0.90 (0.57 - 1.42)	0.653	1.83 (0.97 - 3.46)	0.061
Current	0.96 (0.54 - 1.72)	0.895	1.18 (0.53 - 2.58)	0.688
p-value for trend	0.895		0.688	
Alcohol drinker (yes vs. no)	0.67 (0.42 - 1.09)	0.106	0.90 (0.48 - 1.70)	0.750
AntiHTA ttt (yes vs. no)	1.20 (0.77 - 1.85)	0.417	1.09 (0.59 - 2.02)	0.781
Parental history (yes vs. no)	0.98 (0.61 - 1.58)	0.942	0.52 (0.27 - 0.99)	0.049
CVD risk				
Low	1 (ref.)		1 (ref.)	
Intermediate	0.17 (0.10 - 0.31)	<0.001	0.20 (0.08 - 0.51)	0.001
High	0.05 (0.03 - 0.10)	<0.001	0.06 (0.02 - 0.15)	<0.001
p-value for trend	<0.001		<0.001	
LDL genetic risk score quartiles				
First	1 (ref.)		1 (ref.)	
Second	1.05 (0.62 - 1.79)	0.852	2.74 (1.20 - 6.24)	0.016
Third	1.19 (0.70 - 2.05)	0.519	1.01 (0.49 - 2.10)	0.982
Fourth	1.27 (0.74 - 2.18)	0.380	1.12 (0.53 - 2.35)	0.763
p-value for trend	0.329		0.586	
Number of drugs (per 1 unit)	1.14 (1.04 - 1.25)	0.003	1.15 (1.03 - 1.28)	0.012
Statins potency, no posology				
Low	1 (ref.)		1 (ref.)	
Intermediate	2.14 (1.30 - 3.54)	0.003	2.90 (1.35 - 6.24)	0.007
High	3.01 (1.82 - 4.98)	<0.001	5.13 (2.43 - 10.8)	<0.001

For peer review only - http://bmjopen.bmj.com/site/about/guidelines.xhtml

For peer review only

p-value for trend	<0.001		<0.001	
Fibrates	NC		NC	
Other	0.58 (0.25 - 1.34)	0.202	0.91 (0.30 - 2.76)	0.871

OR, odds ratio; CI, confidence interval; antiHTA ttt, antihypertensive drug treatment; NC, not computable. Results are expressed as odds ratio and (95% confidence interval). Statistical analysis

For peer review only

For peer review only - http://bmjopen.bmj.com/site/about/guidelines.xhtml

Supplemental table 15: multivariable analysis of the factors associated with dyslipidemia control as per the Swiss Group for Lipids and Atherosclerosis criteria. Data from the first (2009-2012) and second (2014-2017) follow-ups of the CoLaus|PsyCoLaus study, Lausanne, Switzerland. Analysis taking into account statin potency and posology

	First follow-up		Second follow-up	
	**OR (95% CI)**	**p-value**	**OR (95% CI)**	**p-value**
Age (per 10 years increase)	1.25 (0.96 - 1.63)	0.102	1.10 (0.76 - 1.60)	0.619
Man vs. woman	1.61 (1.04 - 2.51)	0.034	1.82 (0.97 - 3.42)	0.062
Swiss vs. Non-Swiss	1.25 (0.80 - 1.94)	0.321	0.81 (0.42 - 1.54)	0.515
Education				
High	1 (ref.)		1 (ref.)	
Middle	1.08 (0.53 - 2.19)	0.839	0.47 (0.16 - 1.40)	0.175
Low	0.80 (0.43 - 1.46)	0.465	0.65 (0.23 - 1.79)	0.402
p-value for trend	0.465		0.402	
Married vs. not married	1.33 (0.89 - 2.00)	0.163	1.06 (0.59 - 1.92)	0.842
Body mass index categories				
Normal	1 (ref.)		1 (ref.)	
Overweight	0.86 (0.51 - 1.43)	0.554	0.66 (0.31 - 1.40)	0.283
Obese	0.87 (0.49 - 1.55)	0.641	0.65 (0.28 - 1.48)	0.302
p-value for trend	0.641		0.302	
Smoking categories				
Never	1 (ref.)		1 (ref.)	
Former	0.95 (0.60 - 1.52)	0.836	1.72 (0.89 - 3.34)	0.109
Current	1.05 (0.58 - 1.89)	0.868	1.20 (0.52 - 2.78)	0.672
p-value for trend	0.868		0.672	
Alcohol drinker (yes vs. no)	0.67 (0.41 - 1.10)	0.113	0.93 (0.48 - 1.8)	0.831
AntiHTA ttt (yes vs. no)	1.35 (0.86 - 2.11)	0.187	1.20 (0.63 - 2.28)	0.575
Parental history (yes vs. no)	0.94 (0.58 - 1.52)	0.805	0.67 (0.34 - 1.33)	0.251
CVD risk				
Low	1 (ref.)		1 (ref.)	
Intermediate	0.19 (0.10 - 0.34)	<0.001	0.23 (0.09 - 0.59)	0.002
High	0.06 (0.03 - 0.11)	<0.001	0.07 (0.03 - 0.18)	<0.001
p-value for trend	<0.001		<0.001	
LDL genetic score quartiles				
First	1 (ref.)		1 (ref.)	
Second	1.07 (0.62 - 1.85)	0.800	1.89 (0.81 - 4.39)	0.141
Third	1.34 (0.77 - 2.34)	0.299	0.83 (0.38 - 1.79)	0.634
Fourth	1.25 (0.72 - 2.17)	0.422	1.00 (0.46 - 2.17)	0.997
p-value for trend	0.313		0.514	
Number of drugs (per 1 unit)	1.11 (1.01 - 1.21)	0.028	1.16 (1.02 - 1.30)	0.019
Statins potency, posology				
Low	1 (ref.)		1 (ref.)	
Intermediate	1.36 (0.91 - 2.06)	0.137	1.66 (0.88 - 3.11)	0.115
High	1.67 (0.81 - 3.45)	0.168	2.07 (0.89 - 4.82)	0.092

For peer review only - http://bmjopen.bmj.com/site/about/guidelines.xhtml

p-value for trend	0.168		0.092	
Fibrates	NC		NC	
Other	0.75 (0.31 - 1.84)	0.529	1.64 (0.43 - 6.28)	0.473

OR, odds ratio; CI, confidence interval; antiHTA ttt, antihypertensive drug treatment; NC, not computable. Results are expressed as odds ratio and (95% confidence interval). Statistical analysis by logistic regression.

For peer review only

Supplemental table 16: p-values for the different SNPs associated with statin effect regarding dyslipidemia control, according to the European Society of Cardiology / European Atherosclerosis Society criteria, and the Swiss Group for Lipids and Atherosclerosis strict and near control criteria. Data from the baseline (2003-2006), first (2009-2012) and second (2014-2017) follow-ups of the CoLaus|PsyCoLaus study, Lausanne, Switzerland.

		ESC/EAS			GSLA strict control			GSLA near control		
**Gene**	**SNP**	**Baseline**	**1st FU**	**2nd FU**	**Baseline**	**First FU**	**2nd FU**	**Baseline**	**First FU**	**2nd FU**
ABCB1	rs1128503	0.728	0.896	**0.040**	0.969	0.794	0.167	0.481	0.539	0.513
ABCB1	rs1922242	0.910	0.369	0.147	0.648	0.360	**0.026**	0.902	0.598	0.474
ABCB1	rs2032582	0.835	0.492	0.156	0.887	0.673	0.150	0.387	0.553	0.454
ABCB1	rs3789244	0.728	0.901	**0.040**	0.985	0.779	0.167	0.481	0.539	0.513
ABCG2/BCRP	rs2199936	0.485	0.861	0.444	0.649	0.381	0.141	0.452	0.295	0.507
ABCG2/BCRP	rs2231137	0.094	0.966	0.292	1.000	0.817	0.906	0.671	0.897	0.584
ABCG2/BCRP	rs2231142	0.411	0.894	0.444	0.592	0.310	0.141	0.452	0.329	0.507
ABCG8	rs11887534	0.541	0.671	**0.002**	0.945	0.210	0.196	1.000	**0.031**	0.603
APOA1	rs5069	0.232	0.858	1.000	0.072	0.245	1.000	0.501	0.219	0.680
APOA1	rs670	0.793	0.314	0.125	0.735	0.378	0.386	0.456	0.372	0.833
APOA5	rs662799	0.238	0.052	0.092	0.712	0.792	0.125	0.396	0.437	1.000
APOC1	rs4420638	0.847	0.089	0.567	0.514	0.697	0.085	0.092	0.208	0.503
APOE	rs429358	1.000	0.077	0.502	0.308	0.576	0.784	0.105	1.000	1.000
APOE	rs445925	0.718	0.623	0.788	1.000	0.141	0.856	0.414	0.606	1.000
APOE	rs449647	0.461	0.888	0.207	0.919	0.870	0.617	0.708	0.642	1.000
APOE	rs71352238	0.856	0.269	0.489	0.358	0.952	0.642	0.056	0.761	0.791
APOE	rs7412	0.246	0.154	0.145	0.792	0.694	0.803	0.702	0.347	1.000
CETP	rs5882	0.683	0.749	0.932	0.446	0.773	0.211	0.371	0.105	0.319
CETP	rs708272	0.088	0.841	0.459	0.593	0.580	0.247	0.785	0.182	0.453
CLMN	rs8014194	0.304	0.477	0.382	0.339	0.543	0.636	0.138	0.154	0.527
CYP2C9	rs1057910	0.798	0.064	0.060	0.392	0.060	0.163	0.393	0.064	0.302
CYP2D6	rs3892097	0.781	0.540	0.800	0.328	0.641	0.601	0.619	0.798	0.242
CYP3A4	rs2740574	0.394	0.305	0.430	0.805	0.729	0.220	0.165	0.473	0.703

For peer review only

CYP3A4	rs55951658	NC	NC	NC	NC	NC	NC	NC	NC	NC
CYP7A1	rs3808607	**0.043**	0.573	0.267	**0.017**	0.153	0.601	0.647	0.149	0.766
ESR1	rs2234693	**0.026**	0.938	1.000	0.613	0.868	0.119	0.816	0.797	0.757
ESR1	rs9340799	0.139	0.899	0.569	0.420	0.778	0.156	**0.036**	0.390	0.619
HMGCR	rs12916	0.598	0.406	0.626	0.584	0.912	0.651	0.802	0.139	0.413
HMGCR	rs17238540	0.833	0.552	0.713	0.153	0.901	0.537	1.000	0.450	0.430
HMGCR	rs17244841	0.666	0.498	0.705	0.151	1.000	0.536	1.000	0.442	0.424
HMGCR	rs17671591	0.171	0.947	0.158	0.337	0.388	0.634	0.590	0.124	0.737
HMGCR	rs5908	0.102	0.507	0.632	1.000	1.000	0.466	0.271	1.000	1.000
KIF6	rs20455	0.307	**0.029**	0.209	0.602	0.323	0.609	0.094	0.124	0.058
LDLR	rs5925	0.408	0.311	**0.015**	0.206	0.281	0.348	0.553	0.889	0.935
LPA	rs10455872	0.266	0.659	0.332	**0.021**	0.340	1.000	1.000	0.461	0.452
LPL	rs328	0.372	0.397	0.674	0.186	0.654	0.835	0.087	1.000	0.878
PCSK9	rs17111584	0.462	0.359	0.054	1.000	0.197	1.000	0.380	**0.027**	1.000
PON1	rs662	0.069	0.637	0.911	0.130	0.407	0.684	0.730	1.000	0.749
SCAP	rs12487736	**0.031**	0.145	0.312	0.226	0.525	0.840	**0.032**	0.711	0.413
SCCO1B1	rs2306283	0.076	0.429	0.384	0.533	0.404	0.902	0.719	0.557	0.126
SLCO1B1	rs11045819	0.914	0.768	0.068	**0.020**	0.841	0.250	0.466	**0.044**	0.728
SLCO1B1	rs4149015	0.572	0.819	0.808	0.895	0.084	0.303	0.758	0.438	**0.039**
SLCO1B1	rs4149056	0.112	0.888	**0.011**	0.675	0.574	**0.027**	0.856	**0.045**	0.356
SOD2	rs4880	0.255	0.611	0.460	0.182	0.740	0.837	0.228	0.925	0.214
TLR4	rs4986790	0.654	0.873	**0.013**	0.727	0.124	0.662	1.000	0.166	1.000
ABCB1	rs1045642	0.789	0.500	0.619	0.877	0.160	0.350	0.461	0.578	0.269

NC, not computable as all participants were homozygous. FU, follow-up. Statistical analysis performed using Fisher's exact test. Values <0.05 are indicated in bold.

For peer review only - http://bmjopen.bmj.com/site/about/guidelines.xhtml

References

1. Maji D, Shaikh S, Solanki D, et al. Safety of statins. *Indian J Endocrinol Metab* 2013; 17: 636-646. 2013/08/21. DOI: 10.4103/2230-8210.113754.
2. Stone NJ, Robinson JG, Lichtenstein AH, et al. 2013 ACC/AHA guideline on the treatment of blood cholesterol to reduce atherosclerotic cardiovascular risk in adults: a report of the American College of Cardiology/American Heart Association Task Force on Practice Guidelines. *J Am Coll Cardiol* 2014; 63: 2889-2934. 2013/11/19. DOI: 10.1016/j.jacc.2013.11.002.
3. Catapano AL, Graham I, De Backer G, et al. 2016 ESC/EAS Guidelines for the Management of Dyslipidaemias. *Eur Heart J* 2016; 37: 2999-3058. 2016/08/28. DOI: 10.1093/eurheartj/ehw272.
4. Marques-Vidal P, Rodondi N, Bochud M, et al. Predictive accuracy and usefulness of calibration of the ESC SCORE in Switzerland. *Eur J Cardiovasc Prev Rehabil* 2008; 15: 402-408. 2008/08/05. DOI: 10.1097/HJR.0b013e3282fb040f.
5. Firmann M, Marques-Vidal P, Paccaud F, et al. Prevalence, treatment and control of dyslipidaemia in Switzerland: still a long way to go. *Eur J Cardiovasc Prev Rehabil* 2010; 17: 682-687. 2010/08/12. DOI: 10.1097/HJR.0b013e32833a09ab.
6. Maxwell WD, Ramsey LB, Johnson SG, et al. Impact of pharmacogenetics on efficacy and safety of statin therapy for dyslipidemia. *Pharmacotherapy* 2017; 37: 1172-1190. 2017/07/04. DOI: 10.1002/phar.1981.
7. Spracklen CN, Saftlas AF, Triche EW, et al. Genetic Predisposition to Dyslipidemia and Risk of Preeclampsia. *Am J Hypertens* 2015; 28: 915-923. 2014/12/20. DOI: 10.1093/ajh/hpu242.

For peer review only

For peer review only - http://bmjopen.bmj.com/site/about/guidelines.xhtml

STROBE Statement—Checklist of items that should be included in reports of ***cross-sectional studies***

	Item No	Recommendation	Page No
**Title and abstract**	1	(*a*) Indicate the study's design with a commonly used term in the title or the abstract	1
		(*b*) Provide in the abstract an informative and balanced summary of what was done and what was found	2
**Introduction**			
Background/rationale	2	Explain the scientific background and rationale for the investigation being reported	4
Objectives	3	State specific objectives, including any prespecified hypotheses	4
**Methods**			
Study design	4	Present key elements of study design early in the paper	4
Setting	5	Describe the setting, locations, and relevant dates, including periods of recruitment, exposure, follow-up, and data collection	4
Participants	6	(*a*) Give the eligibility criteria, and the sources and methods of selection of participants	4-5
Variables	7	Clearly define all outcomes, exposures, predictors, potential confounders, and effect modifiers. Give diagnostic criteria, if applicable	5
Data sources/ measurement	8*	For each variable of interest, give sources of data and details of methods of assessment (measurement). Describe comparability of assessment methods if there is more than one group	5-6
Bias	9	Describe any efforts to address potential sources of bias	7
Study size	10	Explain how the study size was arrived at	7
Quantitative variables	11	Explain how quantitative variables were handled in the analyses. If applicable, describe which groupings were chosen and why	7
Statistical methods	12	(*a*) Describe all statistical methods, including those used to control for confounding	7
		(*b*) Describe any methods used to examine subgroups and interactions	NR
		(*c*) Explain how missing data were addressed	NR
		(*d*) If applicable, describe analytical methods taking account of sampling strategy	NR
		(*e*) Describe any sensitivity analyses	7
**Results**			
Participants	13*	(a) Report numbers of individuals at each stage of study—eg numbers potentially eligible, examined for eligibility, confirmed eligible, included in the study, completing follow-up, and analysed	Sup fig 1
		(b) Give reasons for non-participation at each stage	Sup fig 1
		(c) Consider use of a flow diagram	Sup fig 1
Descriptive data	14*	(a) Give characteristics of study participants (eg demographic, clinical, social) and information on exposures and potential confounders	Sup table 6
		(b) Indicate number of participants with missing data for each variable of interest	NR
Outcome data	15*	Report numbers of outcome events or summary measures	

For peer review only

Main results	16	(*a*) Give unadjusted estimates and, if applicable, confounder-adjusted estimates and their precision (eg, 95% confidence interval). Make clear which confounders were adjusted for and why they were included	Tables 1 and 2
		(*b*) Report category boundaries when continuous variables were categorized	NR
		(*c*) If relevant, consider translating estimates of relative risk into absolute risk for a meaningful time period	NR
Other analyses	17	Report other analyses done—eg analyses of subgroups and interactions, and sensitivity analyses	Sup tables 7-13
**Discussion**			
Key results	18	Summarise key results with reference to study objectives	10
Limitations	19	Discuss limitations of the study, taking into account sources of potential bias or imprecision. Discuss both direction and magnitude of any potential bias	12-13
Interpretation	20	Give a cautious overall interpretation of results considering objectives, limitations, multiplicity of analyses, results from similar studies, and other relevant evidence	10-12
Generalisability	21	Discuss the generalisability (external validity) of the study results	12
**Other information**			
Funding	22	Give the source of funding and the role of the funders for the present study and, if applicable, for the original study on which the present article is based	14

*Give information separately for exposed and unexposed groups.

Note: An Explanation and Elaboration article discusses each checklist item and gives methodological background and published examples of transparent reporting. The STROBE checklist is best used in conjunction with this article (freely available on the Web sites of PLoS Medicine at http://www.plosmedicine.org/, Annals of Internal Medicine at http://www.annals.org/, and Epidemiology at http://www.epidem.com/). Information on the STROBE Initiative is available at www.strobe-statement.org.

For peer review only
